# Supplementary material for: Slowest possible replicative life at frigid temperatures for yeast
Source: Nat Commun. 2022 Dec 6;13:7518. doi: 10.1038/s41467-022-35151-2 (PMC9726825; doi:10.1038/s41467-022-35151-2)
Supplement: Supplementary file 1 — Supplementary Information [file 41467_2022_35151_MOESM1_ESM.pdf]

Supplementary Information

Slowest possible replicative life at frigid temperatures for yeast

Diederik S. Laman Trip[1,2], Théo Maire[1,2], and Hyun Youk[2,3*]

[1]Kavli Institute of Nanoscience, Lorentzweg 1, 2628CJ Delft, The Netherlands

[2]Department of Systems Biology, University of Massachusetts Chan Medical School, 368 Plantation Street, Worcester MA 01605, USA

[3]CIFAR Azrieli Global Scholars Program, CIFAR, 661 University Ave. Suite 505, Toronto ON M5G 1M1, Canada

[*]Corresponding author: hyun.youk@umassmed.edu

This document contains:

-Supplementary Figures 1 - 47

-Supplementary Notes (starts on Pg. 77)

-Supplementary Discussion (starts on Pg. 86)

-Supplementary References (starts on Pg. 88)

Supplementary Figures

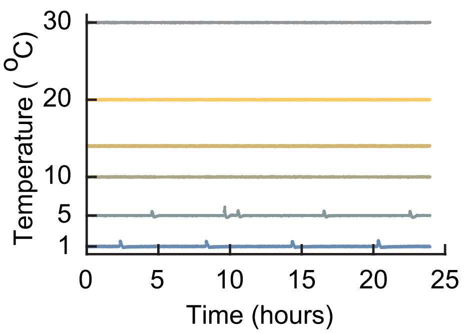

Supplementary Fig. 1: Temperature remains stable during all our growth experiments (Related to Figure 1a). All growth experiments were performed with liquid cultures of cells incubated in compressor-cooled, high-precision thermostatic incubators (Memmert ICPs). The ICPs had a precise temperature-recording device whose temperature readings we additionally verified using a separate thermocouple device and aligned with the temperatures of several other incubators. The temperature was monitored over the entire course of our experiments. As examples, shown here are the temperatures as recorded by the incubator's temperature sensor for 24 hours during six different growth experiments. Starting from the top, the curves show the temperature of the incubator set at 30.0 °C, 20.0 °C, 14.0 °C, 10.0 °C, 5.0 °C and 1.0 °C. Throughout our experiments, the incubators had a typical standard deviation of 0.033 °C when the incubation temperature was above 10.0 °C. The standard deviation was 0.09 °C when the incubation temperature was below 10.0 °C. This slightly larger standard deviation was due the incubator undergoing short thaw-cycles to prevent freezing of its components (deviation measured over several days).

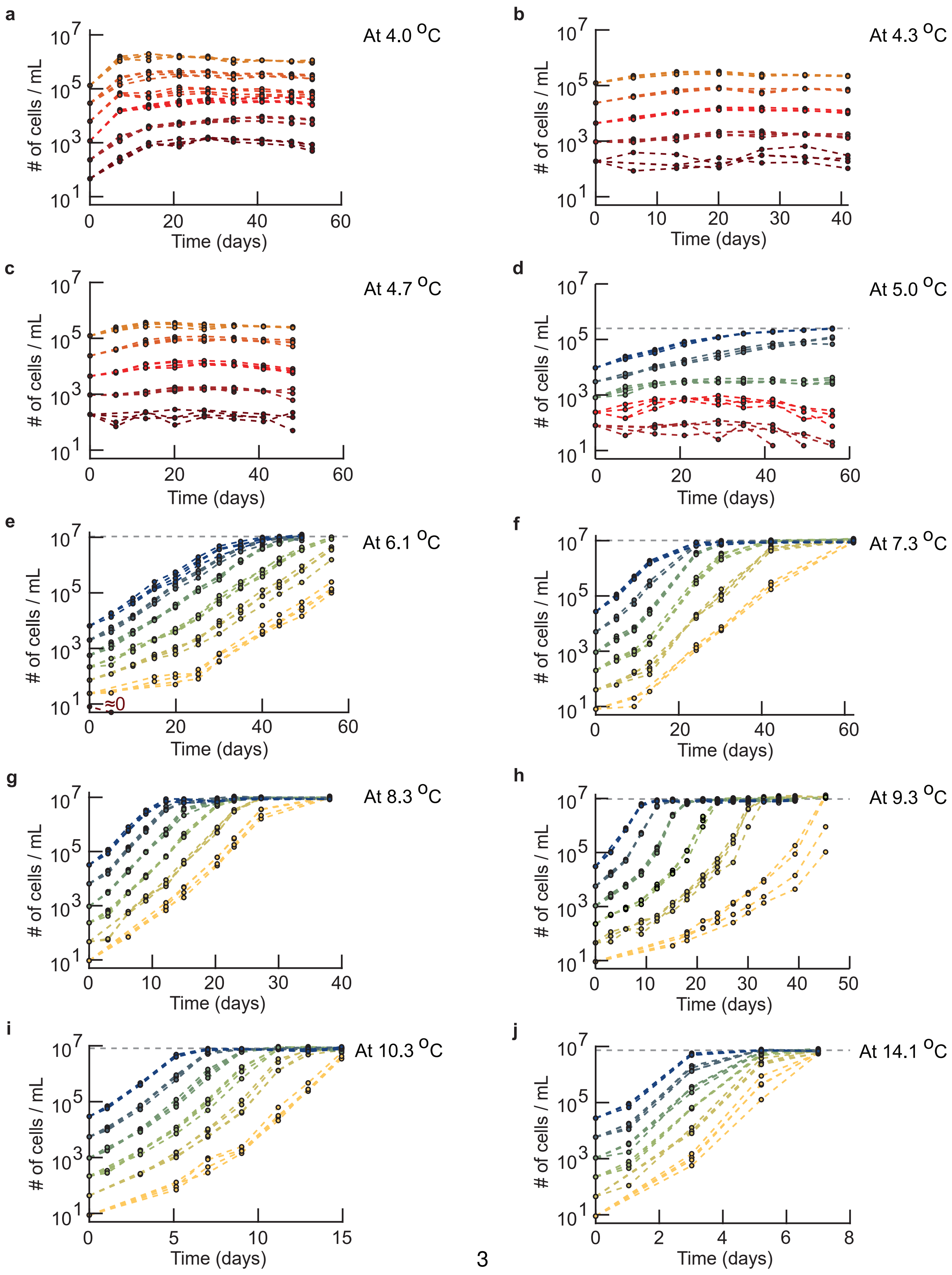
a
At 4.0 °C
b
At 4.3 °C
c
At 4.7 °C
d
At 5.0 °C
e
At 6.1 °C
≈0
f
At 7.3 °C
g
At 8.3 °C
h
At 9.3 °C
i
At 10.3 °C
j
At 14.1 °C
# of cells / mL
Time (days)

Supplementary Fig. 2: Growth curves for widely varying initial population densities and temperatures (Related to Figure 1a-b). Population density (number of cells / mL) measured over time with a flow cytometer. Shown are populations of wild-type yeast with differing initial densities incubated at 4.0 °C **(a)**, 4.3 °C **(b)**, 4.7 °C **(c)**, 5.0 °C **(d)**, 6.1 °C **(e)**, 7.3 °C **(f)**, 8.3 °C **(g)**, 9.3 °C **(h)**, 10.3 °C **(i)** and 14.1 °C **(j)**. Different colors represent different initial population-densities. The grey dotted lines show the carrying capacity that we estimated from the final densities of the populations. To construct the phase diagram (Figure 1b), we used the growth-kinetics data as shown here to determine whether a population with a given initial population-density should be characterized as growing ("growth" phase) or non-growing ("no growth" phase). An initial population-density was characterized as being in the growth phase (Fig. 1b – blue region) if all replicate populations that started with that density exponentially grow over time and reach the carrying capacity for that temperature. An initial population-density was characterized as being in the no-growth phase (Fig. 1b – red region) if all replicate populations that started with that density did not grow during several weeks of incubation, except for some initial transient growth that results from the cells having been transferred from 30.0 °C. This transient growth typically lasted a few days. For example, every population in (c) belongs to the no-growth phase because no population grew during the ∼6 weeks of incubation at 4.7 °C as can be seen by every population density barely increasing over time. As another example, after some lag-time, every population in (g) grew exponentially and identically over time to reach the carrying capacity at 8.0 °C. To draw the phase boundary that separates the growth phase from the no-growth phase in the phase diagram (Fig. 1b), we connected the maximum initial density for which a population does not grow for each temperature. This curve almost overlaps with the minimum initial density that leads to population growth for each temperature because we sampled the initial densities close to each other for each temperature. Similarly, we drew the boundary curve that separates the growth phase from the region of the phase diagram where nutrients are lacking by connecting the data points that represent, for each temperature, the measured carrying capacity (grey lines in d-j). Finally, we determined the temperature below which no population growth is possible – and thus only the no-growth phase exists below this temperature – by identifying the highest temperature (i.e., 4.7 °C (c)) where populations with different starting densities always reach differing final densities (as opposed to the common carrying capacity). In fact, these populations do not grow at all beyond the initial, transient growths (a-c). All panels show n = 4 replicate populations for each initial population-density (color).

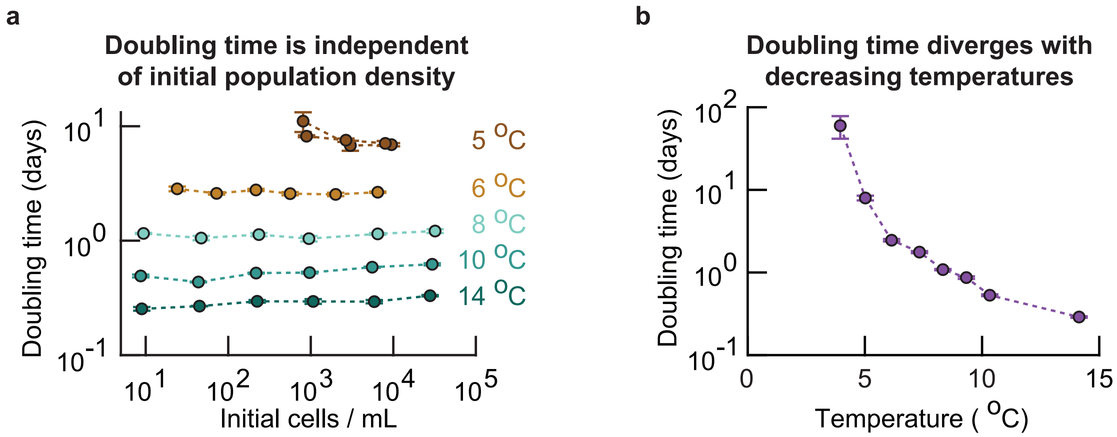

Supplementary Fig. 3: Population's doubling time is independent of its initial density at near-freezing temperatures (Related to Figure 1b). Summarizing Supplementary Fig. 2. From the growth curves of populations at various temperatures, we determined each population's doubling time. To obtain the doubling time of the populations that grew (i.e., populations in the "growth" phase of the phase diagram, Fig. 1b), we excluded the initial transient growth that typically lasted from a few days to one week. For non-growing populations (e.g., populations in the "no-growth" phase at 4.0 °C) we only took the growth rates that were positive and excluded the initial transient growth (i.e., transient growth in the first week of incubation). **(a)** Population doubling time as a function of initial density and temperature for growing populations. The doubling time does not depend on initial density. Data shown as mean ± s.e.m. For each temperature, every data point represents mean from n = 4 biological replicate populations. **(b)** Since the doubling time does not depend on initial density, we pooled all observed doubling times for each temperature. The population doubling time diverges as the temperature decreases. Data are shown as mean ± s.e.m. for *n* = 16 biological replicate populations (at 4 °C), *n* = 21 biological replicate populations (at 5 °Cand 7 °C), *n* = 40 biological replicate populations (at 6 °Cand 8 °C), or *n* = 24 biological replicate populations (at 9 °C, 10 °C, and 14 °C).

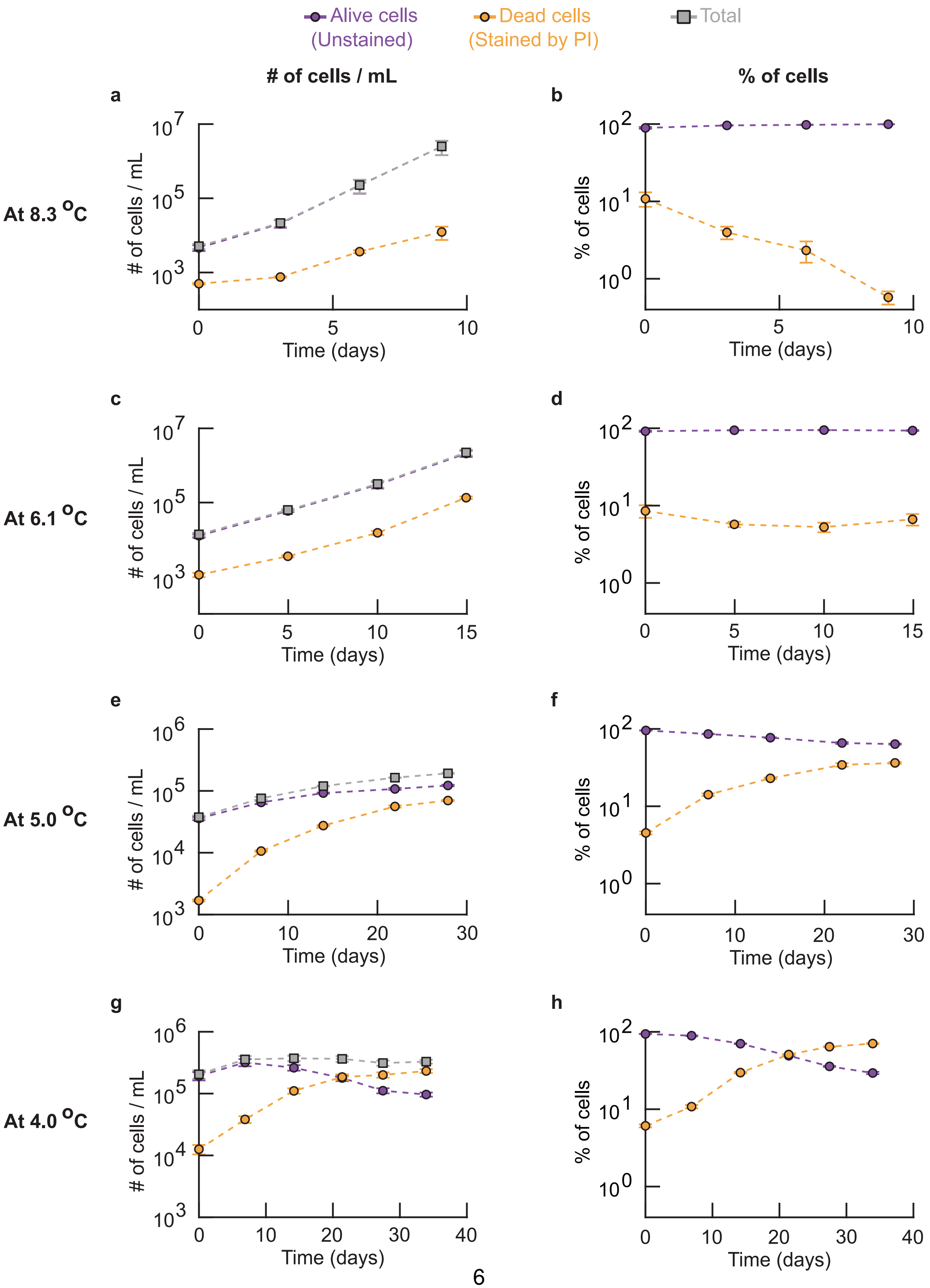

Alive cells (Unstained)
Dead cells (Stained by PI)
Total
# of cells / mL
% of cells
a
b
c
d
e
f
g
h
At 8.3 °C
At 6.1 °C
At 5.0 °C
At 4.0 °C
# of cells / mL
% of cells
Time (days)

Supplementary Fig. 4: Population extinction occurs below 5 °C because as the temperature decreases, the average doubling time of a cell increases and eventually, just below ∼5 °C, becomes larger than the average cell's lifespan (Related to Fig. 1b). Left column **(a, c, e, g)**: number of dead cells (yellow), number of alive cells (purple) and total number of cells (grey) in a population. Right column **(b, d, f, h)**: percentages of dead (yellow) and alive (purple) cells in a population. For each population, we took an aliquot of the liquid culture at various times and incubated it with 1 μg / mL of propidium iodide (PI) for 20 minutes at room temperature. We then flowed this aliquot through a flow cytometer to measure the total number of cells and the number of cells that were stained (red) and unstained (yellow) by PI. PI does not stain cells with an intact membrane [1]. In contrast, PI enters cells with a damaged membrane and stains their DNA. Thus, propidium iodide stains a cell if and only if it's membrane is permeable. Hence, alive cells are commonly assumed to be impermeable to PI. Shown here are populations of wild-type yeast incubated at 8.3 °C **(a-b)**, 6.1 °C **(c-d)**, 5.0 °C **(e-f)** and 4.0 °C **(g-h)**. The curves show the total population-density and the density of stained and unstained cells over time **(a, c, e** and **f)**, together with the percentage of cells that were stained and unstained in the population **(b, d, f** and **h)**. All populations had already been incubated for at least 6 days at the respective temperature before the PI staining on day 0 in each graph (i.e., the populations had already spent ∼6 days at 8.3 °C (a-b), ∼10 days at 6.1 °C (c-d), and ∼14 days at 5.0 °C (e-f) or 4.0 °C (g-h)). Error bars show the mean with s.e.m., with n = 4 replicate populations per data point. **(a-b)** Alive and dead cells over time at 8.3 °C. All populations grow exponentially over time. Both the density of alive and dead cells increase exponentially over time, with the density of alive cells increasing faster than the density of dead cells. Specifically, the percentage of dead cells in the population decreases exponentially over time. Thus, at 8.3 °C, alive cells overtake the population as cell replication occurs more frequently than cell death (purple curve in (b) stays at ∼100%). **(c-d)** Alive and dead cells over time at 6.1 °C. Similar to the results at 8.3 °C. The main difference here is that the percentage of dead cells in the population remained constant over time (yellow curve in (d)), whereas the percentage of dead cells decreased over time at 8.3 °C (see (a)). Like in 8.3 °C, nearly 100% of the population consists of alive cells at 6.1 °C. **(e-f)** Alive and dead cells over time at 5.0 °C. The density of alive cells increases over time. However, the percentage of dead cells in the population increases while the percentage of alive cells decreases over time. Still, the populations consists of more alive cells than dead cells after ∼6 weeks of incubation at 5.0 °C (see (f)). **(g-h)** Alive and dead cells over time at 4.0 °C. The population does not grow and the density of alive cells decreases over time (g) (i.e., less than one cells divides per cell that dies). The percentage of dead cells increases exponentially over time while the percentage of alive cells decreases, leading to the population eventually consisting mostly of dead cells (the population becomes extinct as this trend continues over time) (see (h)). ***(caption continues)***

Supplementary Fig. 4 ***(continued)***: Together, (a-h) show that as the temperature decreases, the average population doubling time increases and eventually exceeds the average lifespan of a cell. Having a doubling time that is larger than the average lifespan means that the average cell does not have time to replicate before it dies. This leads to a population extinction. In other words, populations grow more slowly as temperature decreases and eventually, at around 5.0 $^\circ$C, populations cannot exponentially grow because the average doubling time nearly matches the average lifespan of cells. Finally, at 4.0 $^\circ$C, the doubling time exceeds the lifespan of cells and thus populations cannot grow.

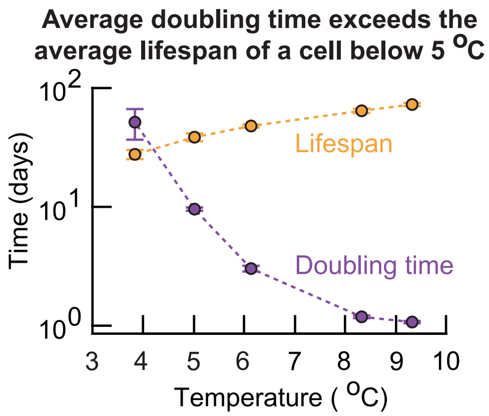

Supplementary Fig. 5: Average doubling time of a cell increases and eventually becomes larger than the average lifespan of a cell as the temperature decreases below 5 °C (Related to Figure 1b). Summarizing Supplementary Fig. 4. We used our measurements of the number of alive and dead cells in populations at each temperature to extract the average doubling time and the average lifespan of cells. Specifically, we fitted a growth model to the data that gives the expected lifespan and doubling time of cells at the population level (Supplementary Theory). Shown is the average doubling time (purple) and average lifespan (orange) at various temperatures. The average doubling time exceeds the average lifespan when the temperature is below 5.0 °C. Consequently, below 5.0 °C, the average cell dies before it has a chance to replicate and hence the population approaches extinction. Data shown as mean ± s.e.m., for n = 16 biological replicate populations (at 4 °C), n = 8 biological replicate populations (at 5 °C), n = 15 biological replicate populations (at 6 °C), n = 16 biological replicate populations (at 8 °C), or n = 12 biological replicate populations (at 9 °C).

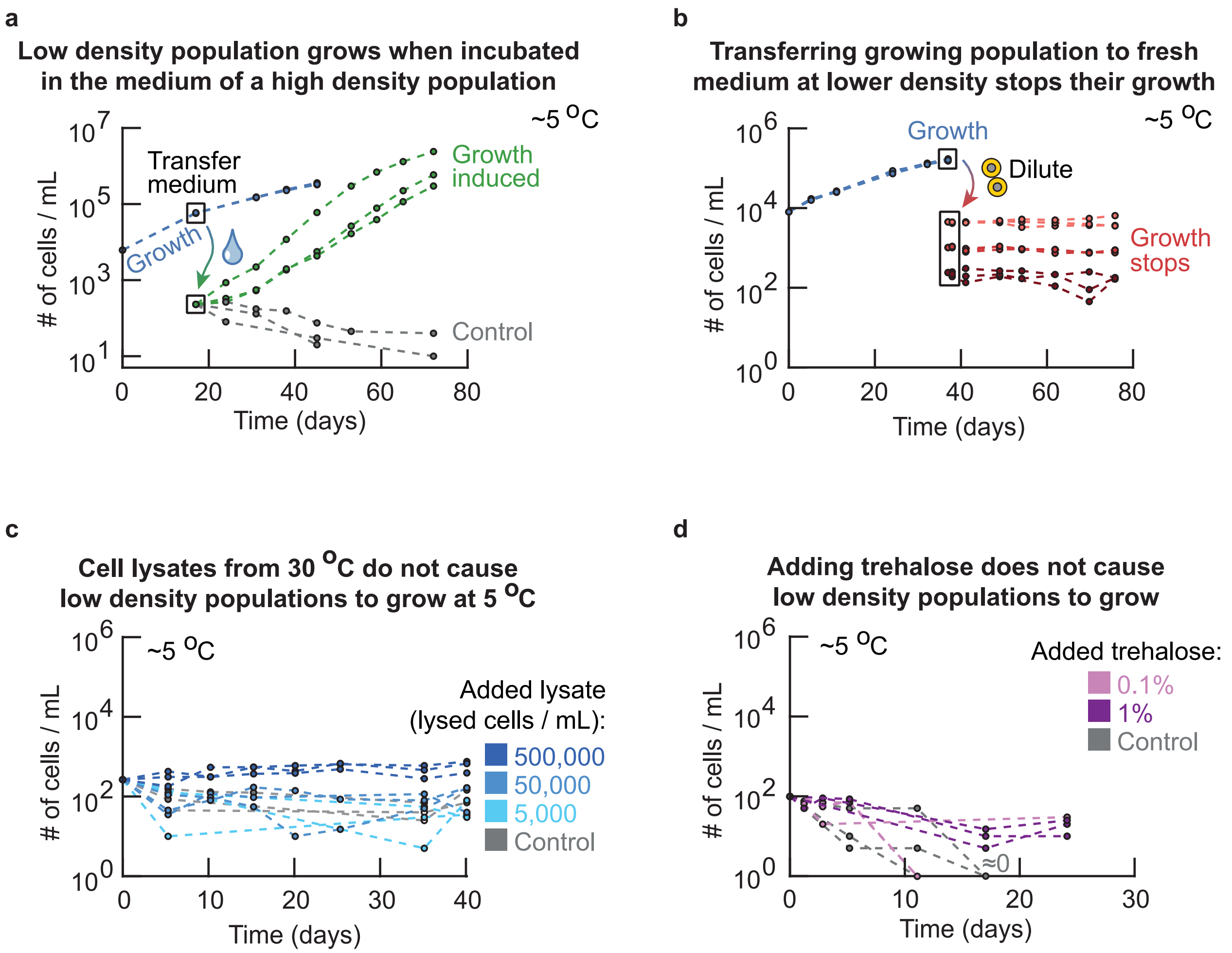

Supplementary Fig. 6: Secreted factor in extracellular medium determines whether a population grows or not at 5 °C (Related to Figure 2a). Testing why high-density populations grow whereas low-density populations do not grow at the same near-freezing temperature (5.0 °C). **(a)** To test whether the cell's ability to replicate at 5.0 °C is dictated by secreted factor(s) in the extracellular medium, we took the growth medium of a high-density (growing) population (blue curves, initially ∼6,250 cells / mL) after several weeks of growth at 5.0 °C. Boxed data points on the blue curves show when the growth medium was taken. We flowed the high-density culture through a membrane filter with 0.2 μm pores so that growth medium taken from this culture was free of cells, which we confirmed with a flow cytometer (i.e., no cells were detected). Next, into this filtered medium, we incubated a fresh, low-density population of cells that were growing in 30 °C (green curves, initially ∼250 cells / mL). As a control, we also incubated the same, low-density of cells in a fresh growth medium (grey curves, initially ∼250 cells / mL). We incubated the two low-density cultures at 5.0 °C and measured their cell numbers over time. The populations of fresh cells grew in the filtered growth medium of the high-density population at 5.0 °C (green curves). But the low-density (control) populations in the fresh medium did not grow (grey curves). ***(caption continues)***

Supplementary Fig. 6 ***(continued)*****:** This experiment shows that cells change their growth medium (e.g., through secreted factor(s)) such that they can grow at 5.0 °C, and that a sufficiently high density of cells is required for the change to be sufficient for a population growth. **(b)** To test whether the ability to replicate at 5.0 °C is determined by intracellular factor(s) (e.g., a heritable trait), we took an aliquot of cells from a high-density (growing) population at 5.0 °C and diluted it by various amounts into fresh, pre-cooled media at 5.0 °C to test whether the resulting low-density population could grow. The high-density population of wild-type cells (blue curves, initially ∼10, 000 cells / mL) grew at 5.0 °C. After ∼35 days of growth, we took an aliquot of cells from this culture and diluted it by ∼50x (light red curves, initially ∼10, 000 cells / mL), ∼250x (red curves) or ∼1250x (dark red curves). Ordinarily, populations that start at these "low densities" do not grow at 5.0 °C (Fig. 1b). We incubated the diluted, low-density populations in fresh medium at 5.0 °C and measured their density over time (red curves). None of these populations grew at all during more than one month of incubation at 5.0 °C. This experiment shows that a population's ability to grow at 5 °C is not solely determined by intracellular factor(s). **(c)** To test whether the extracellular factors that enable population growth come from dying cells that may be bursting (lysing), we incubated populations in media with cell lysates. We obtained the cell lysates from a population of wild-type cells growing in log-phase at 30.0 °C (∼5, 000, 000 cells / mL). We added glass beads to this liquid culture and placed it on a vortex for 20 min to mechanically lyse the cells. After this, we flowed the liquid media containing the lysed cells with the beads through a 0.45 μm pore filter to remove the glass beads and any intact cells that remained. We then added the filtered cell lysate to a fresh medium in amounts dictated by the cell densities before lysis (e.g., fresh medium having 500, 000 lysed cells / mL has a 90% volume of fresh medium and ∼10% cell lysate that had ∼5, 000, 000 cells / mL before the lysis). Finally, we incubated fresh cell populations in these growth media at a low density (∼250 cells / mL) and at 5.0 °C. The growth curves show populations with ∼500, 000 lysed cells / mL (dark blue curves), ∼50, 000 lysed cells / mL (blue curves), ∼5, 000 lysed cells / mL (light blue curves) and, as control, fresh cells in a fresh medium without any cell lysate (grey curves). None of these populations grew at 5.0 °C. This experiment shows dying cells that may be bursting are not responsible for inducing population growth at 5.0 °C. **(d)** Trehalose is a common cryoprotectant that is abundant in yeasts at low temperatures and is thought to protect the cells against freezing [2]. Its precise mechanism is incompletely understood. We reasoned that yeast populations may be secreting trahalose to help them grow at 5.0 °C. To test this idea, we incubated a low-density population at 5.0 °C (initially ∼100 cells / mL) with 1% trehalose (dark purple curves, percentage in weight per volume), 0.1% trehalose (light purple curves) or without trehalose (grey curves). None of the populations grew, indicating that trehalose is not one of the extracellular factor(s) that induce growth at 5.0 °C. All panels show at least n = 3 biological replicates per condition (color).

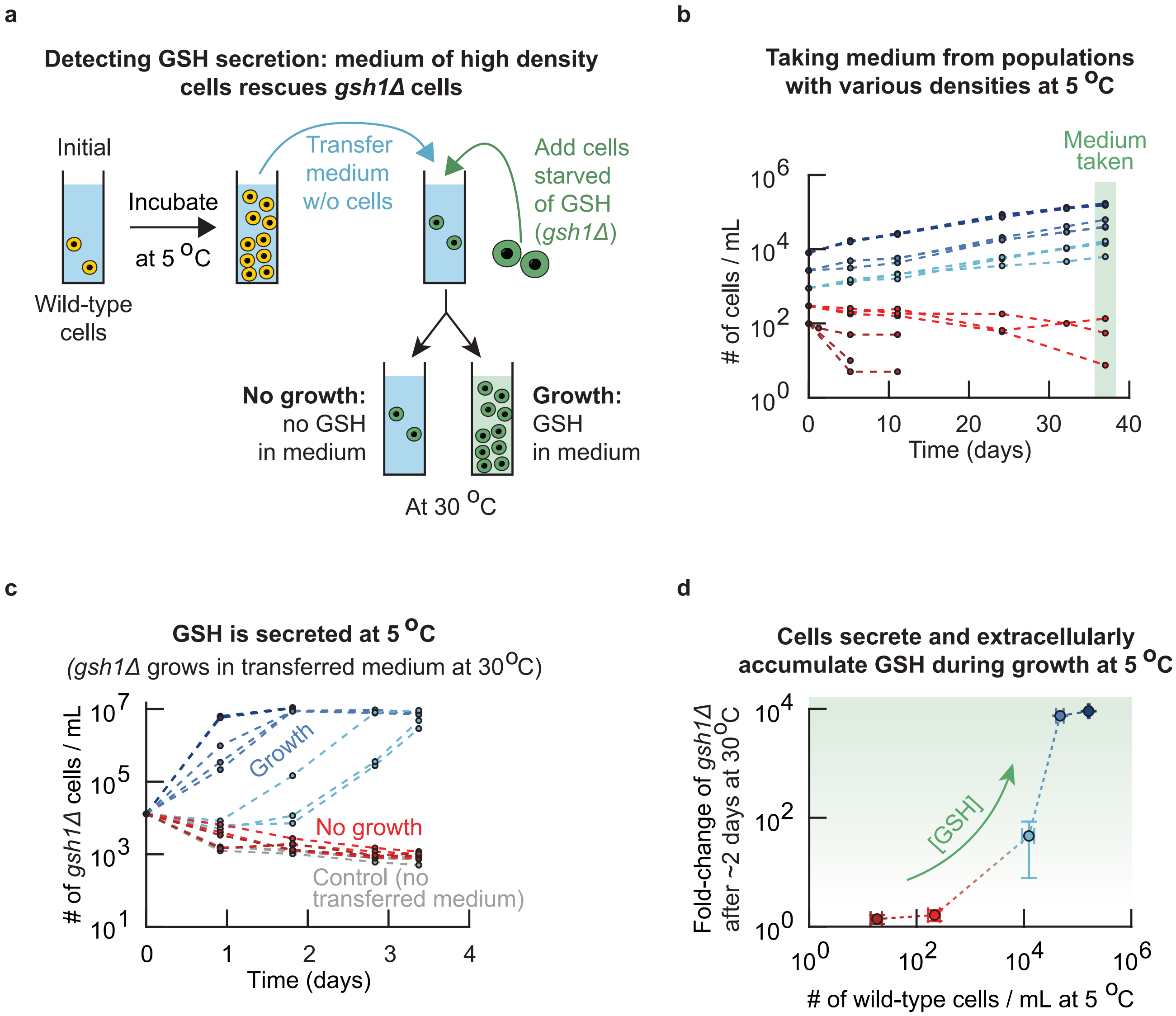

Supplementary Fig. 7: A non-viable mutant that cannot synthesize glutathione (*gsh1*■) is rescued by medium from high-density populations, which confirms that cells in high-density populations secrete and extracellularly accumulate glutathione at 5 °C (Related to Figure 2a). (a) Schematics of experiments in (b-d). A mutant that cannot synthesize glutathione (*gsh1*Δ-strain) cannot live without supplemented GSH even at 30 °C, because GSH is essential for life (e.g., GSH is essential for iron metabolism [3]). The only way for the mutant to survive and grow at any temperature is by supplementing GSH into its growth medium [4, 5]. To test whether cells at 5.0 °C secrete glutathione, we incubated wild-type yeast populations at 5.0 °C at different initial population-densities. After several weeks of incubation, we isolated the growth medium from the wild-type populations. Specifically, we took aliquots of the cultures at 5.0 °C and removed the cells from the growth media by spinning down the aliquot and passing the supernatant through a 0.2 μm pore filter to remove all yeasts. ***(caption continues)***

Supplementary Fig. 7 ***(continued)*****:** We checked that no cells remained in the filtered media by flowing them through a flow cytometer (no events detected). In the filtered media, we then incubated a population of *gsh1*Δ cells that we had starved of glutathione by incubating them overnight in fresh medium without GSH at 30.0 °C (these cells therefore did not grow during the overnight). If the filtered media did not contain any glutathione, then the starved *gsh1*Δ cells would not grow. If the filtered media did contain glutathione, secreted by the wild-type cells, then the *gsh1*Δ cells would be able to import the glutathione and grow. **(b)** As a first part of the experiment described in (a), we incubated wild-type yeast populations at 5.0 °C and took aliquots of their growth media after ∼5 weeks of incubation. Shown is the density of wild-type populations over time for different starting densities (∼300 cells / mL (red curves), ∼900 cells / mL (light blue curves), ∼2, 700 cells / mL (blue curves) and ∼8, 000 cells / mL (dark blue curves)). Each color shows n = 3 replicate populations. **(c)** To detect extracellular glutathione, we isolated and filtered the growth media from the wild-type populations in (b). Shown is the population density over time at 30.0 °C of glutathione-starved *gsh1*Δ cells that received the filtered media (initially ∼13, 000 cells / mL). The colors of the curves here match the colors used for the various densities of wild-type populations in (b) (e.g., the red populations received the filtered media from the wild-type populations whose growth curves are shown in red in (b)). As a control, we also incubated *gsh1*Δ cells in fresh medium without any glutathione (grey curves). The *gsh1*Δ cells did not grow in the medium transferred from low-density (non-growing) populations at 5.0 °C. The *gsh1*Δ cells always grew to the carrying capacity in the medium from the high-density (growing) populations at 5.0 °C. Each color shows n = 3 replicate populations. **(d)** Summary of the data from (b) and (c). The x-axis shows the population density of wild-type cells after ∼5 weeks of incubation at 5.0 °C. The y-axis shows, after two days of incubation at 30 °C, the number of *gsh1*Δ cells in transferred media relative to the number of *gsh1*Δ cells in a fresh medium without glutathione. Populations of *gsh1*Δ cells that grew more had more glutathione in their medium (glutathione is the growth limiting factor in the transferred media, see control in (c)). Error bars represent the mean with s.e.m., having n = 3 biological replicates per data point. Together, (a-d) show that the amount of extracellular glutathione increases with population density for growing populations at 5.0 °C, and that barely any extracellular glutathione accumulates for non-growing populations at 5.0 °C. In summary, cells in growing populations secrete and extracellularly accumulate glutathione at near-freezing temperatures.

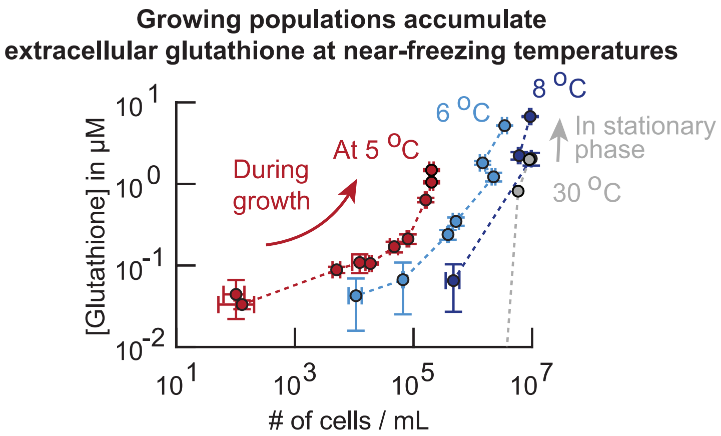

Supplementary Fig. 8: Cells secrete and accumulate glutathione during growth at near-freezing temperatures (Related to Figure 2a). With an enzymatic assay kit, we quantified the total glutathione concentration in the growth media of wild-type yeast populations at two different time points after at least 2 weeks of incubation at various temperatures (Methods). Shown here is the total extracellular glutathione concentration as function of population density at ∼5.0 °C (red curve, one week between sampling), ∼6.1 °C (light blue curve), ∼8.3 °C (dark blue curve) and ∼30.0 °C (grey curve). The extracellular glutathione concentration increases during population growth at near-freezing temperatures (i.e., 5.0 °C - 8.3 °C), whereas glutathione only accumulates in stationary phase at 30.0 °C. Error bars show the mean with s.e.m., having n = 3 biological replicates per data point.

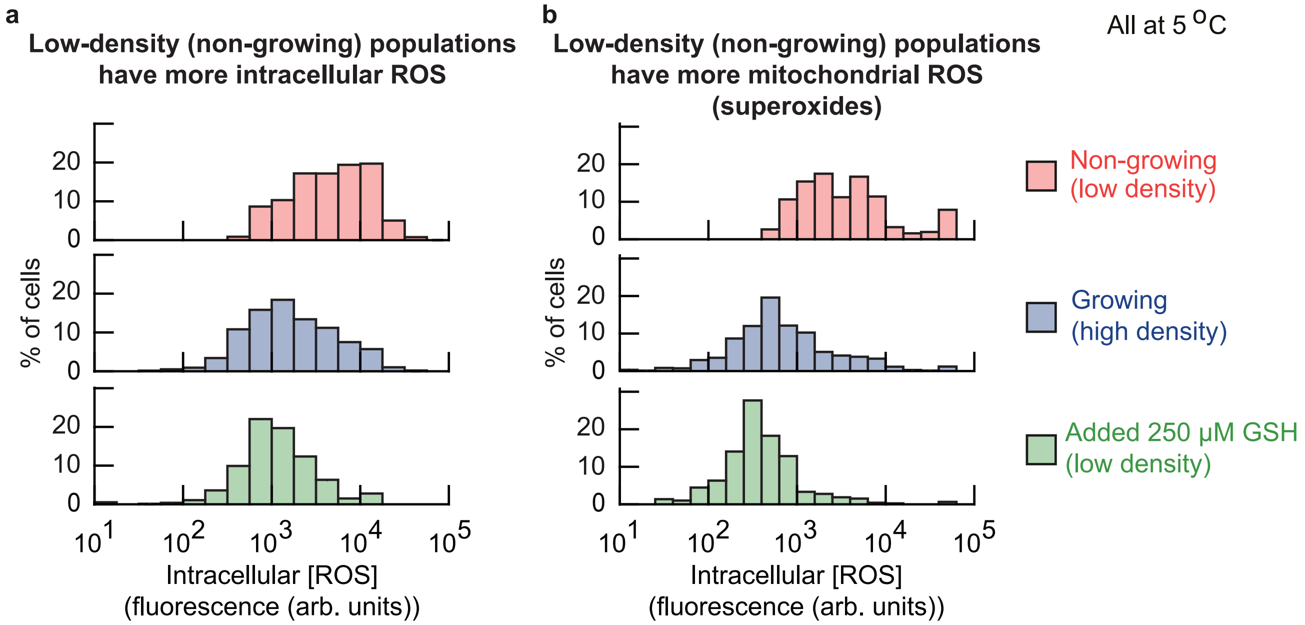

Supplementary Fig. 9: Cells of low-density (non-growing) populations at 5 °C have more intracellular Reactive Oxygen Species (ROS), including superoxides in their mitochondria, which can be removed by supplementing extracellular reduced glutathione (GSH) (Related to Figure 2d). (a-b) Measured intracellular ROS concentrations in single cells. We stained intracellular ROS in live, single cells with two different dyes. One dye (cellROX) measured the amount of general, cytoplasmic ROS while another dye (mitoSOX) measured the amount of superoxides in mitochondria. We incubated populations of cells at 5.0 °C for two weeks and then stained the cells with the ROS dyes (Methods). We then used fluorescence microscopy to determine the amounts of intracellular ROS in single cells, represented by the average intracellular fluorescence of the dye in each cell (after subtracting background fluorescence). **(a)** Intracellular ROS concentration for general ROS (with cellROX). **(b)** Superoxide concentrations in mitochondria (with mitoSOX). Histograms in (a-b) show cells of low-density (non-growing) populations (red bars, initially ∼250 cells per ml), growing populations (blue bars, initially ∼6, 250 cells / mL) and low-density populations that had 250 μM GSH added to their growth media (green bars, initially ∼250 cells per ml). These histograms show that the low-density (non-growing) populations have higher intracellular ROS concentrations than high-density (growing) populations. Adding extracellular GSH decreased the intracellular ROS concentrations of the low-density populations to concentrations similar to the high-density populations. Each histogram is an average histogram and is representative of $n = 3$ biological replicate populations.

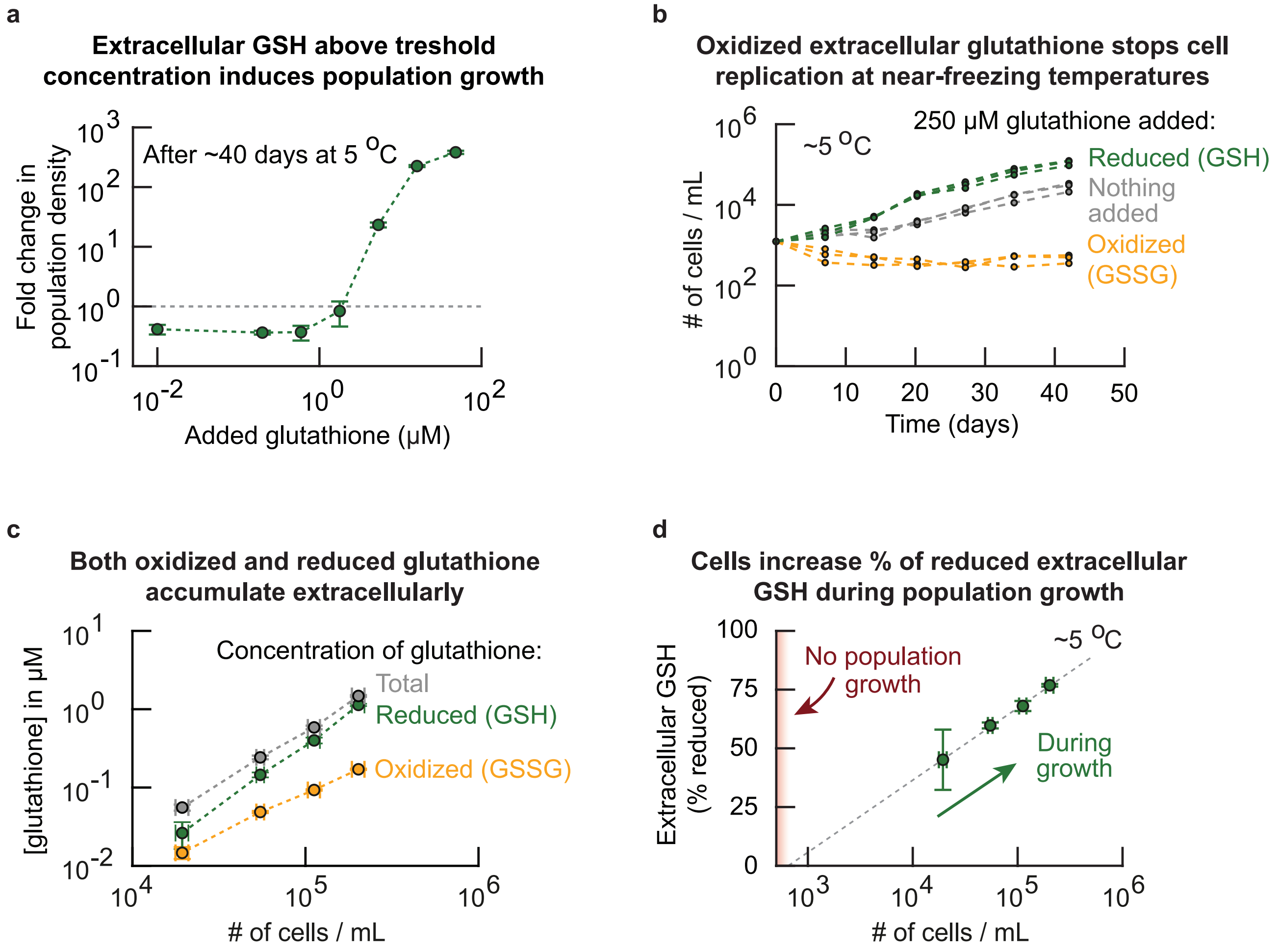

Supplementary Fig. 10: Reduced form of glutathione, not the oxidized form of glutathione, enables and accelerates replications at 5 °C above threshold concentration of ∼1 μM (Related to Figure 2d). Unless stated otherwise, our experiments added the reduced form of glutathione (GSH). But for the experiments in this figure, we used both reduced glutathione (GSH) and oxidized glutathione (GSSG) to determine that GSSG does not induce growth at 5.0 °C. **(a)** We incubated a low-density wild-type population (initially ∼260 cells / mL) at 5.0 °C with various amounts of added GSH. The GSH concentration spanned almost four orders of magnitude. Shown here is the fold-change of the population density after ∼40 days of incubation. Populations expanded when the extracellular GSH concentration exceeded ∼1 μM, while populations shrank if the extracellular GSH concentration was less than ∼1 μM. Thus, low-density populations require at least 1 μM of GSH to grow at 5.0 °C. This threshold concentration is high compared to the ∼0.1 μM of GSH that the high-density populations accumulated themselves (Fig. 2a). This result suggests that there are other extracellular factors that induce population growths at 5.0 °C. ***(caption continues)***

Supplementary Fig. 10 ***(continued)*****:** There are two results that support this: (1) removing extracellular GSH does not completely stop growth of high-density populations (Supplementary Fig. 11); and (2) the concentration of secreted GSH by high-density (growing) populations is an order of magnitude below the concentration of the GSH that must be added to cause a low-density population to grow at 5.0 °C. Error bars show the mean with s.e.m., having n = 3 replicate populations per data point. **(b)** So far, we established that extracellular GSH can induce population growths (a), and it is the antioxidant action of GSH that induces growth (Supplementary Fig. 11). Next, we wondered whether cells at 5.0 °C specifically require reduced glutathione to induce growth. The reduced form of glutathione, GSH, functions as an antioxidant whereas the antioxidant function does not exist in the oxidized form, GSSG (two oxidized glutathione molecules). We incubated cells at 5.0 °C with either oxidized or reduced glutathione in their growth medium. Shown here are the population densities over time for 250 μM reduced glutathione (GSH, green curves), 250 μM oxidized glutathione (GSSG, yellow curves) and without any glutathione added to the medium (grey curves, all initially at $\sim$1,240 cells / mL). All populations grew when either GSH or nothing was added to the medium, with populations having added GSH growing faster. In contrast, the populations that had GSSG (oxidized glutathione) added to their medium did not grow at all. These results show that populations require GSH (reduced form of glutathione) to grow at 5.0 °C. This finding is supported by the fact that it is glutathione's antioxidant action that induces population growth (Supplementary Fig. 11). Each condition (color) shows n = 3 replicate populations. **(c)** We next measured the concentrations of both oxidized and reduced extracellular glutathione that accumulated in the medium of high-density (growing) populations (Methods). Shown are the total (grey curve), reduced (green curve) and oxidized (yellow curve) extracellular glutathione concentrations in the same medium after 28 days at 5.0 °C for various population densities. The total extracellular glutathione concentration increases as the initial population density increases (also see Fig. 2a). Both the concentrations of reduced and oxidized glutathione increase with density, with the concentration of reduced glutathione increasing faster. Error bars show the mean with s.e.m., having n = 3 biological replicates per density. **(d)** Using the data from (c), we plotted the percentage of extracellular GSH (the percentage of all glutathione that is reduced) as a function of the population density. Grey dotted line shows a linear fit as a visual guide. The percentage of GSH increases with population density. Extrapolating the linear fit suggests that all extracellular glutathione would be oxidized in the medium of populations with a density below $\sim$1,000 cells / mL (i.e., non-growing populations, Fig. 1a). Together, our data ((c-d) and Fig. 2a) establish that cells in high-density populations secrete and accumulate a pool of extracellular glutathione, with higher density populations having higher concentrations of glutathione that are increasingly reduced. Moreover, these results show that populations cannot grow when the entire pool of extracellular glutathione becomes oxidized (b). Error bars show the mean with s.e.m, having n = 3 biological replicates per data point.

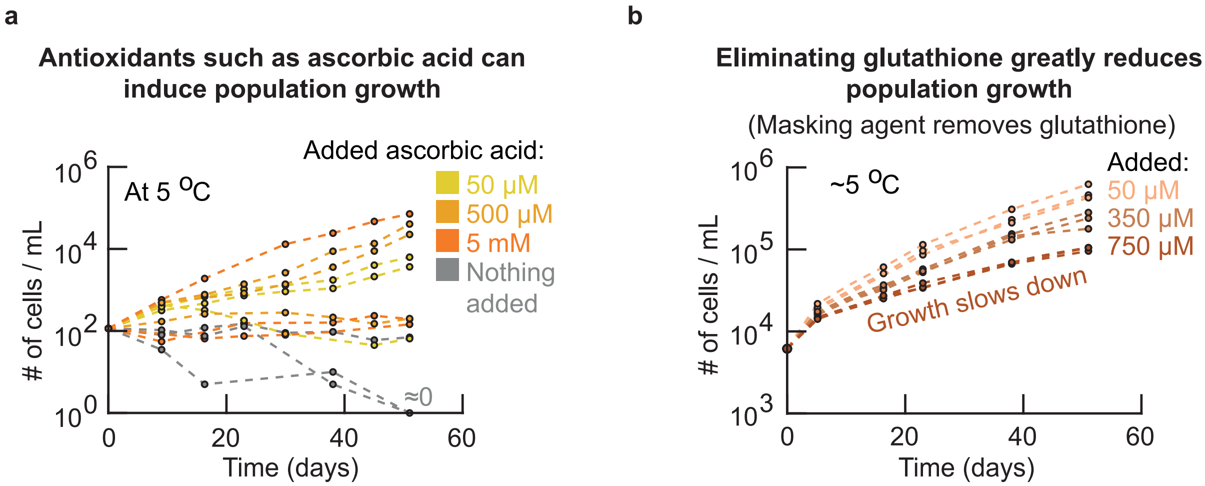

Supplementary Fig. 11: Supplementing the growth medium with an extracellular antioxidant (ascorbic acid or glutathione) is sufficient for inducing growth at 5 °C (Related to Figure 2d). So far, we have shown that an ample extracellular GSH can induce population growths at 5.0 °C (Fig. 2c) and that cells secrete and extracellularly accumulate glutathione, with a sufficient amount for growth being secreted only by sufficiently high-density populations (Fig. 2a, Supplementary Fig. 7). **(a)** To show that it is glutathione's role as an antioxidant, as opposed to its other roles (e.g., regulating iron metabolism [3]), that causes the population growths, we incubated wild-type yeast populations at 5.0 °C in medium supplemented with ascorbic acid, an antioxidant that yeasts do not synthesize [6]. Shown here is the population density measured over time for low-density populations (∼100 cells / mL) incubated with 50 μM ascorbic acid (yellow curves), 500 μM ascorbic acid (orange curves), 5 mM ascorbic acid (red curves) or without any ascorbic acid (grey curves). More than half of the populations that had extracellular ascorbic acid grew at 5.0 °C, whereas the control populations without ascorbic acid did not grow at all (because the initial population-density was too low for growth according to our phase diagram, Fig. 1b). This result supports the idea that the antioxidant action of glutathione alone is sufficient to induce population growths since other antioxidants such as ascorbic acid can also induces population growths. **(b)** Knowing that glutathione's antioxidant role is sufficient to induce population growths, we next tested whether glutathione is absolutely necessary for growth at 5.0 °C. We used a masking agent (1M2VP) that specifically and functionally blocks extracellular glutathione in the growth medium [7, 8]. We incubated wild-type cell populations at 5.0 °C (initially ∼6,250 cells / mL) with 50 μM of masking agent (beige curves), 350 μM of masking agent (light brown curves) or 750 μM of masking agent (dark brown curves). We found that the masking agent reduces population growth, with more masking agent reducing growth further. The masking agent does not fully prevent population growth at any concentration. Extracellular GSH is therefore sufficient for inducing growth and is important for accelerating duplications at 5.0 °C, but does not seem to be absolutely necessary for populations to grow at 5.0 °C. Together, our results suggest that there is (are) likely additional extracellular factor(s) that control population growth. ***(caption continues)***

Supplementary Fig. 11 ***(continued)*****:** This is because we established that secreted factor(s) determine whether populations grow (Supplementary Fig. 6), high-density populations continue to grow without extracellular glutathione (b), and low-density populations are not able to grow at 5.0 °C (Fig. 1a). Each color shows n = 3 replicate populations in both panels.

GSH increases the percentage of cells that replicate and decreases the single-cell doubling time at 5 °C

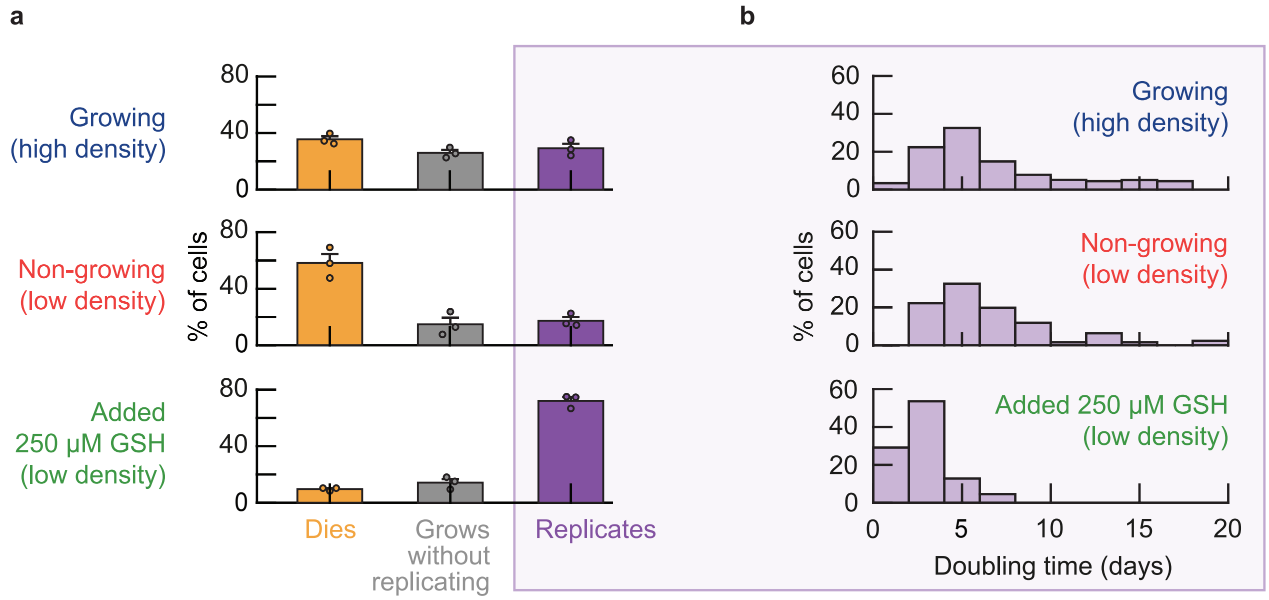

Supplementary Fig. 12: Glutathione increases the percentage of cells that replicate and decreases their doubling time at 5 °C (Related to Figure 2e). Comparing the behavior of single cells at near-freezing temperatures with and without reduced glutathione ("GSH") added to the growth medium. **(a)** We incubated wild-type cell populations at 5.0 °C with or without adding GSH to their growth medium. After two weeks of incubation, we followed whether individual cells replicated, grew without replicating or died with a microscopy time-lapse (Methods). Shown here are the percentages of cells that replicated (purple bar), died (orange bar) and grew without replicating (grey bar) during the ∼20 days of imaging. We did these measurements for high-density (growing) populations (initially ∼6, 250 cells / mL), low-density (non-growing) populations (initially ∼250 cells / mL), and low-density (growing) populations with 250 µM of added GSH (initially ∼250 cells / mL). Note that 250 µM GSH is higher than the ∼1 µM of glutathione that the high-density populations accumulate by themselves (Fig. 2a). The added GSH decreases the percentage of cells that die in the low-density population to 9.7 ± 0.7% (compared to ∼58% in populations of the same density without added GSH). Similarly, the added GSH increases the percentage of cells that replicates in the low-density population to 72.1 ± 2.7% (compared to ∼17% in the low-density populations without added GSH). Thus, extracellular GSH decreases cell deaths and increases cell replications. Bars show the mean with s.e.m., having n = 3 biological replicates per condition. Dots show raw data. **(b)** Doubling time for single cells in each population. Histogram shows the percentage of cells with a given doubling time. The average doubling time is 6.5 days for the high-density populations (n = 451 cells) and 7.1 days for the low-density populations (n = 42 cells). Thus, the doubling time of replicating cells is similar regardless of population density. ***(caption continues)***

Supplementary Fig. 12 *(continued)*: In contrast, the average doubling time is 2.9 days for the low-density populations with added GSH (n = 444 cells). GSH therefore reduces the time it takes to replicate by more than half. Each histogram is an average histogram and is representative of n = 3 biological replicate populations. Together (a-b) show that both high-density (growing) and low-density (non-growing) populations contain replicating cells, with nearly identical doubling time distributions. The main differences between the two populations are the percentage of cells in each population that replicate and the percentage of cells that die. Adding extracellular GSH increases the percentage of replicating cells (a) and decreases the doubling time of these cells (b). Data for populations without added GSH is reproduced here from Fig. 1c as a comparison.

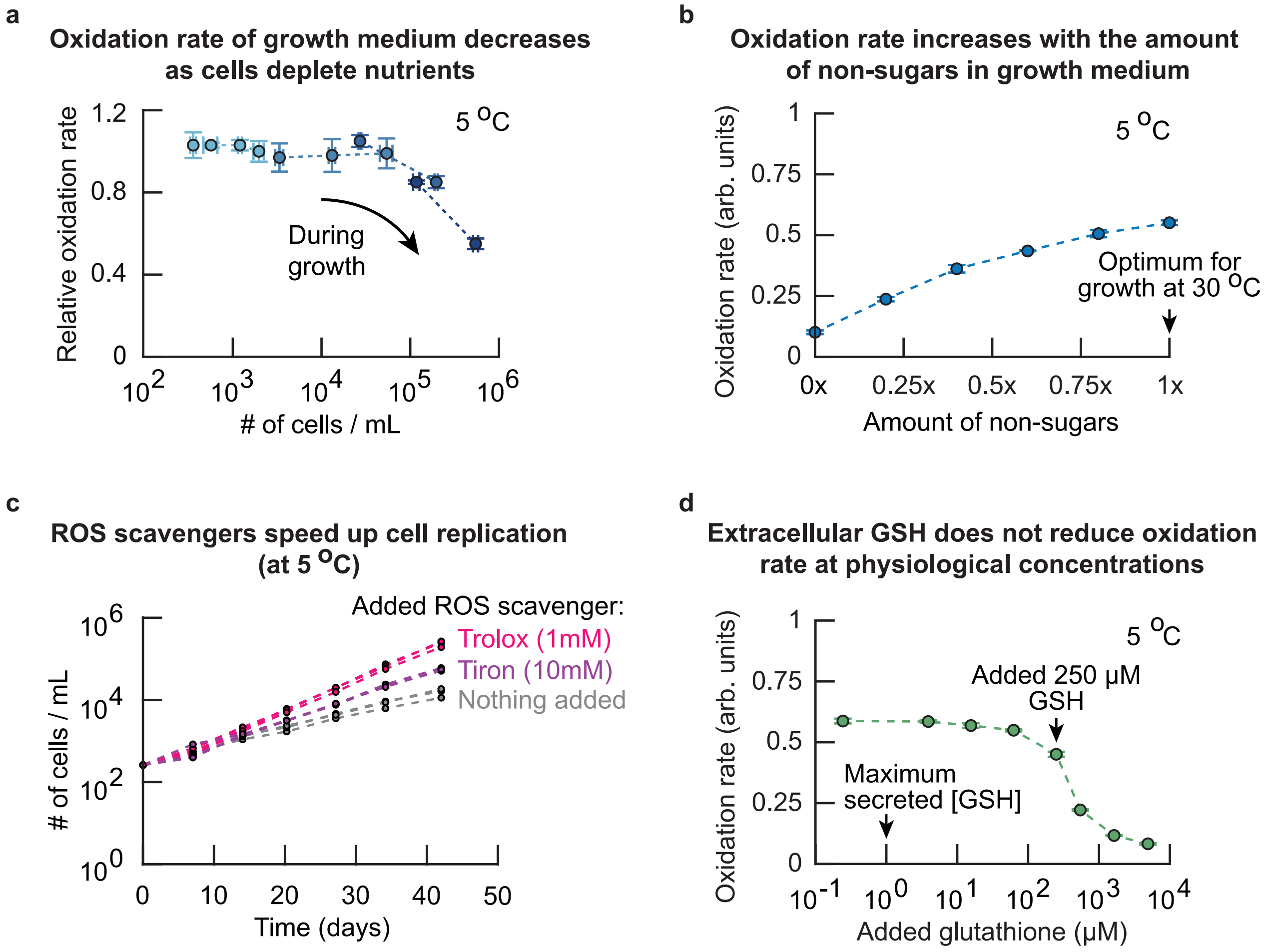

Supplementary Fig. 13: Non-sugar components of the yeast growth medium (e.g., vitamins, amino acids) and oxygen create Reactive Oxygen Species without any cells at 5 °C (Related to Figure 2f). Together, our results suggest that more extracellular factor(s) – besides reduced glutathione – dictate population growth at near-freezing temperatures (Supplementary Figs. 6 and 11-10). Knowing that extracellular factor(s) dictate growth at near-freezing temperatures (Fig. 6), and that cells are stressed by ROS (Fig. 2a, Supplementary Fig. 9) and that it is the antioxidant action of GSH that induces population growth (Supplementary Figs. 11-10), we hypothesized that the extracellular environment may also be a source of ROS. **(a)** To test whether ROS are present in the extracellular environment, we used a dye called dihydroethidium (DHE) that becomes fluorescent upon oxidation by ROS (mainly superoxides). We grew wild-type yeast populations with various starting densities at 5.0 °C, and measured the oxidation rate of their growth media relative to the oxidation rate of fresh media. Here, the oxidation rate is given by the rate at which the oxidation-responsive dye becomes fluorescent. To do so, at every time-point, we took aliquots of our liquid cultures and flowed the aliquot through a 0.2 μm pore filter. We then measured the oxidation rate in the flow-through – the growth medium without any cells – and compared with the oxidation rate in fresh medium. ***(caption continues)***

Supplementary Fig. 13 *(continued)*: Shown is the relative oxidation rate as function of population density. The oxidation rate decreases when the population density exceeds $\sim 10,000$ cells / mL. This result shows that ROS are present in the extracellular environment of cells and that the oxidation rate of the medium decreases with population density. **(b)** Given that the oxidation rate decreases with population density, we further hypothesized that the components of the growth medium – which the cells consume and deplete – may be a source of ROS. We therefore tested whether the presence of any of the nutrients in medium affects the ROS production in the cell's environment. Specifically, without cells, we measured the oxidation rate of DHE at 5.0 °C in growth media having various compositions. We diluted the growth medium with water by various amounts and then supplemented these diluted media with 2% glucose (so a "0.5x-medium" contains 0.5x SC + 0.5x water, supplemented with 2% glucose). Shown is the oxidation rate in diluted media at 5.0 °C (blue curve, in steps of 0.2x non-sugars). We found that the oxidation rate increased roughly linearly with the amount of non-sugar nutrients in the medium (i.e., the concentrations of amino acids, vitamins, trace elements, salts and the nitrogen source). **(c)** Knowing that the extracellular environment may be a source of ROS through the non-sugar nutrients in the growth medium, we added chemicals to the growth medium that remove (i.e., have scavenging activity against) ROS. Specifically, we used scavengers of superoxide (10 mM tiron) or peroxyl radicals (1 mM trolox) and tested whether the scavenging agents could be beneficial for yeast growth. To do so, we grew populations of cells at 5.0 °C in the presence of ROS scavengers (initially $\sim$250 cells / mL). Shown is the population density over time for populations with trolox (pink curves), tiron (purple curves) or nothing (grey curves) added to the growth medium. Both trolox and tiron increase the growth rate of populations. Thus, scavenging and removing extracellular ROS (such as superoxide and peroxyl radicals) is beneficial for population growth at near-freezing temperatures. **(d)** Finally, we tested the effect of GSH on the oxidation rate of the growth medium. Shown is the oxidation rate as a function of the GSH concentration in minimal medium without cells at 5.0 °C. Increasing the GSH concentration above a threshold concentration – at $\sim$250 μM GSH – decreases the oxidation rate. For all panels the error bars are mean with s.e.m., with $n = 3$ replicates per condition.

In summary (a-d) show that non-sugar nutrients in the growth medium increase the oxidation rate of the environment by facilitating the generation of ROS. The oxidation rate decreases when these non-sugar nutrients are removed from the growth medium. Potential sources of ROS in the environment are oxygen (superoxide) or non-sugar nutrients themselves (peroxyl radicals), as demonstrated by scavenging of these ROS being beneficial for yeast growth at near-freezing temperatures. Finally, we found that GSH can decrease the extracellular oxidation rate at high concentrations.

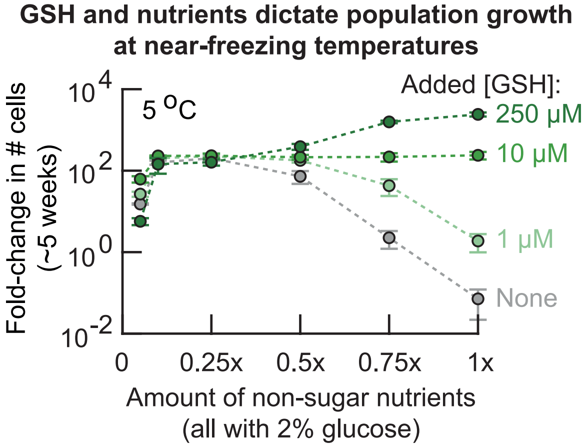

Supplementary Fig. 14: Population growth is a balance between extracellular GSH and availability of non-sugar nutrients at 5 °C (Related to Figure 2i). Studying the combined effect of non-sugar nutrients and extracellular GSH on population growth. We incubated cells in media that had precise amounts of non-sugar nutrients and added GSH. Specifically, for varying the amount of non-sugar nutrients, we formulated the media to contain 0.05x, 0.1x, 0.25x, 0.5x, 0.75x or 1x non-sugar nutrients. Here, 1x equals the amount of non-sugar nutrients that is in the medium for yeast grown under standard conditions (Methods). Each of these media had 2% glucose. Then, for each of these medium compositions, we added either 0 μM, 1 μM, 10 μM or 250 μM of GSH. This procedure created 24 different media (6 different amounts of non-sugar nutrients each with 4 different added GSH concentrations). In these media we incubated low-density populations of cells (initially ∼210 cells / mL) at 5.0 °C. These low-density populations should not grow according to our phase diagram (Fig. 1b). Shown is the fold-change in population density as function of the amount of non-sugar nutrients after ∼5 weeks of incubation at 5.0 °C. The curves represent 1 μM (light green curve), 10 μM (green curve), 250 μM (dark green curve) or without (grey curve) added GSH. The populations in 1x non-sugar nutrients and without added GSH did not grow at all during the 5 weeks of incubation at 5.0 °C, as expected from our phase diagram (Fig. 1b). Further corroborating our other data we find that at 1x non-sugar nutrients, the fold-change in population density increased with the concentration of added GSH (also see Fig. 2e) and that without added GSH, the fold-change in population density increased when the amount of non-sugar nutrients decreased (grey curve, also see Fig. 2g). Then, starting at 1x non-sugar nutrients, all curves converge to a ∼100-fold change in population density as the amount of non-sugar nutrients decreased from 1x to 0.25x. Thus, adding more than 10 μM GSH is only beneficial for population growth when sufficient non-sugar nutrients are available (i.e., more than 0.5x non-sugar nutrients). Finally, populations stop growing when the amount of non-sugar nutrients decreased below 0.1x. ***(caption continues)***

Supplementary Fig. 14 *(continued)*: In summary, this growth experiment shows that either sufficient extracellular GSH (more than 1 μM, Supplementary Fig. 10) or low enough amounts of non-sugar nutrients are required for population growth at 5.0 °C. Adding glutathione or removing non-sugar nutrients induces growth by either removing ROS (Supplementary Figs. 9-11) or decreasing the oxidation rate (Supplementary Fig. 13). Populations having less non-sugar nutrients require less extracellular GSH for growth, and populations having more extracellular GSH can grow with more non-sugar nutrients. The media with more than 0.75x non-sugar nutrients and 250 μM added GSH are special because they permit growths that exceed the (∼100-fold) growth that a population can achieve by secreting glutathione by itself (light green curve; also see Fig. 2a). Error bars show the mean with s.e.m., having n = 3 biological replicates per condition.

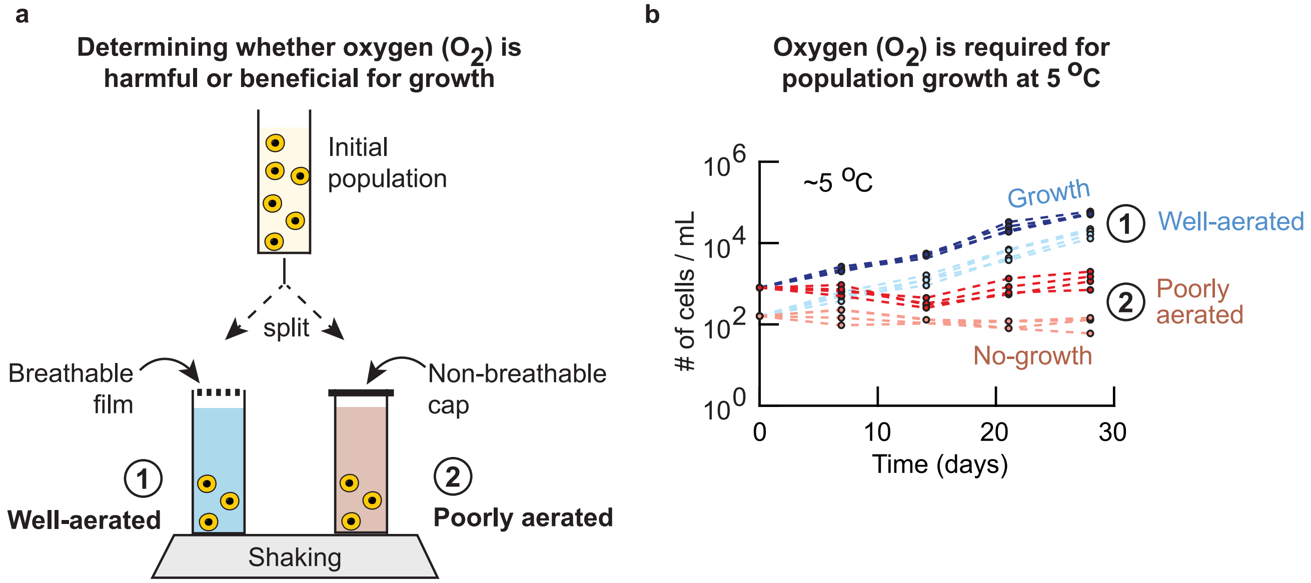

Supplementary Fig. 15: Oxygen (O_2) is required for population growth at 5 °C (Related to Figure 2i). Testing whether oxygen (O_2) is required or harmful for population growths at 5.0 °C. **(a)** Schematic of the experiment in (b). We incubated 5 mL of cell populations that were either well aerated or poorly aerated at 5.0 °C. For well-aerated conditions, cells were grown in tubes with a breathable film sealing the tube tops (Methods). For poorly aerated conditions, the cells were in 5 mL Eppendorf tubes that were sealed off by a non-breathable cap and parafilm. This created two populations of cells that started with the same density but one that was well-aereated (oxygen available through the air) and one that was poorly aerated (limited oxygen). Both populations were incubated at 5.0 °C and continuously shaken at 125 rpm. **(b)** Results of the experiment described in (a). Shown here are the population densities over time for populations that were well-aerated (blue curves, labelled ”1”) or poorly aerated (brown curves, labelled ”2”), each condition having different initial densities (∼350 cells / mL or ∼100 cells / mL). All well-aerated populations grew exponentially over time. Poorly aerated populations barely increased in density during the experiment. These results show that aeration (sufficient availability of oxygen in the growth medium) is necessary for population growth at 5.0 °C. Thus, oxygen is both beneficial (required for cell replications) and harmful (as source of ROS, Supplementary Fig. 13) at 5.0 °C. This result complements our findings that non-sugar nutrients the growth medium are sources of ROS (Supplementary Fig. 13). Each condition (color) shows $n = 4$ biological replicates.

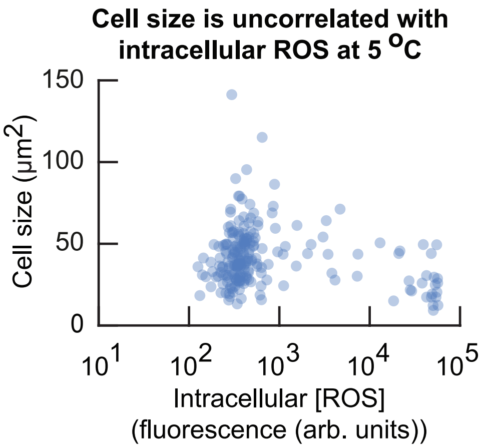

Supplementary Fig. 16: Cell size does not correlate with the intracellular ROS concentration at 5 °C (Related to Figure 3c). Cell size as a function of intracellular ROS concentration. We incubated populations of cells for two weeks at 5.0 °C (initially ∼6, 250 cells / mL). We then measured the size and intracellular ROS concentration in single cells (Methods). This result shows that the cell size is uncorrelated with intracellular ROS concentration. Dots show single-cell data aggregated from $n = 3$ biological replicates ($n = 214$ cells).

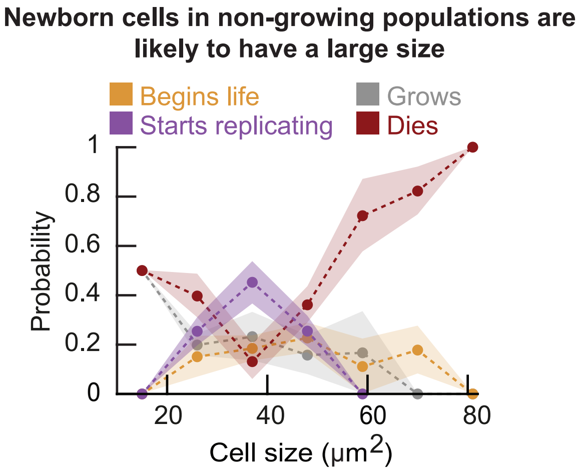

Supplementary Fig. 17: Newborn cells in low-density (non-growing) populations are larger than those in high-density (growing) populations at 5 °C (Related to Figure 3c). We incubated populations of wild-type cells at a low density (initially ∼500 cells / mL) for two weeks at 5.0 °C and then imaged these cells in our wide-field microscope for the next several weeks (Methods). We classified individual cells in the resulting time-lapse movies as being in one of the following events (Supplementary Fig. 18): "begins life" (just-born daughter cell), "starts replicating", "grows" (without replicating), or "dies" (cell just before death). For every cell in each class, we measured its cell size. Specifically, for cells in the "begins life" class, we measured the daughter cell's size just after cytokinesis (when the mother's bud neck breaks). For cells in the "starts replicating" class, we measured the mother cell's size at the time the bud appears. For cells in the "grows" class, we measured their size in the first time point when the movie begins (i.e., at the first moment that they are growing without replicating). For cells in the "dies" class, we measured the size just before they die (i.e., the last time point before death). We binned the cells according to their sizes. For each bin, we determined the probability that a cell in that size bin would be in each of the four classes. Dots show average probability in each bin for the different events, shaded area represents the s.e.m. for n = 3 biological replicates. This graph shows that newborn cells in low-density (non-growing) populations are likely to have a large size, instead of being smallest cells in the population (Fig. 3c). Thus, newborn cells in low-density (non-growing) populations are less likely to replicate than the mother cells in the same population (see Supplementary Fig. 18).

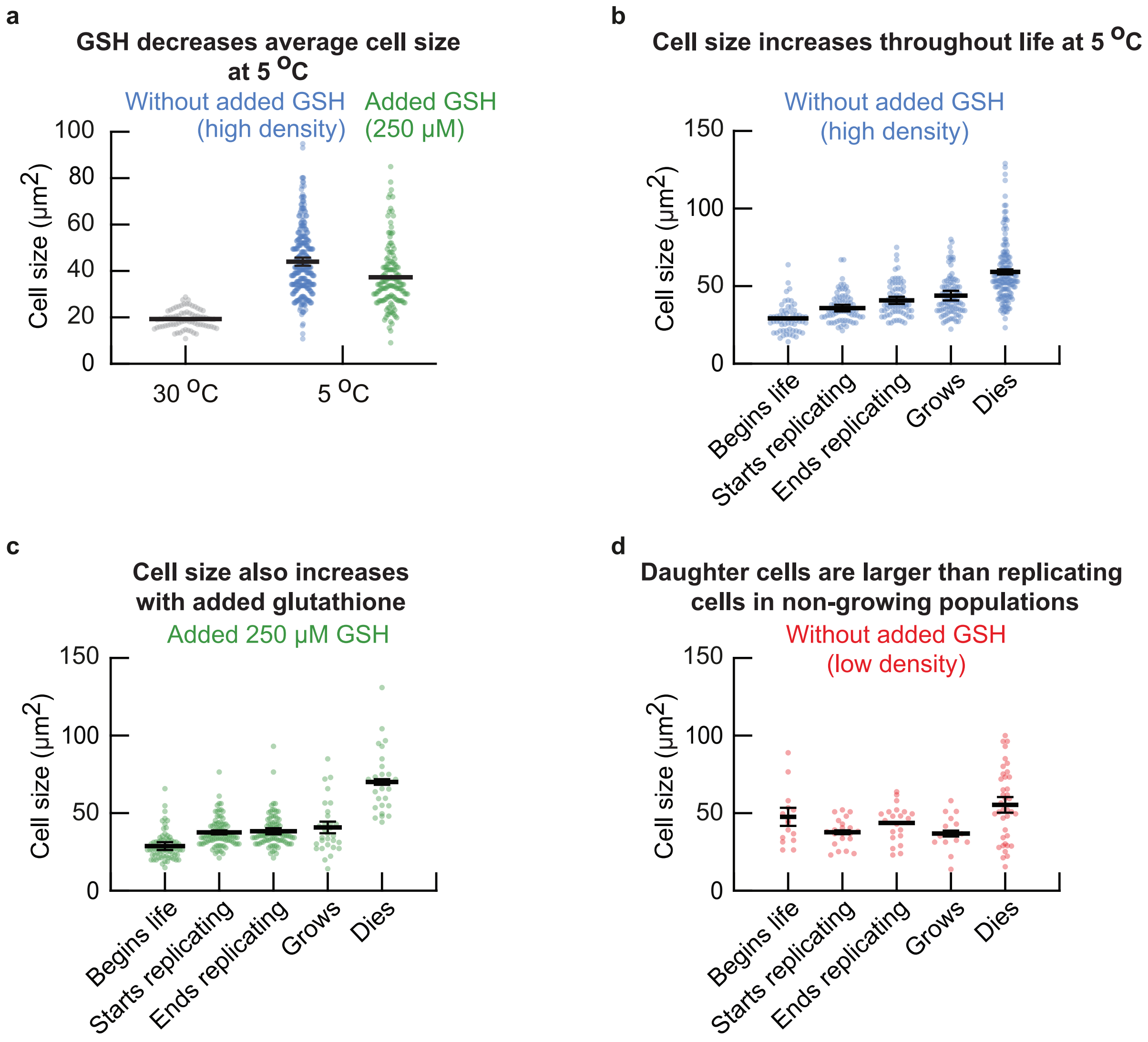

Supplementary Fig. 18: Cell size monotonically increases from birth to death at 5 °C (Related to Figure 3c). (a) We incubated wild-type cell populations for two weeks at 5.0 °C and then measured the cell sizes with a wide-field microsope. Shown here are the cell sizes of individual cells from high-density populations without any GSH added (blue dots, initially ∼8,000 cells / mL) and sizes of individual cells from low-density populations that were incubated with 250 μM GSH (green dots, initially ∼420 cells / mL). Grey dots show cells during growth at 30.0 °C as a comparison. Surprisingly, we find that cells are, on average, more than double the size (cross-sectional area) at 5.0 °C (44.0 ± 1.7 μm^2) compared to 30.0 °C (19.3 ± 0.2 μm^2). Moreover, adding GSH to the growth medium decreases the average cell size at 5.0 °C (to 37.3 ± 0.1 μm^2). Error bars represent the mean with s.e.m., having n = 3 biological replicates. Dots show data aggregated from the biological replicates. ***(caption continues)***

Supplementary Fig. 18 *(continued)*: (b-d) Next, we incubated populations of wild-type cells for two weeks at 5.0 °C. After two weeks we transferred aliquots of the liquid cultures to microscopy plates that we kept chilled at 5.0 °C before the transfer and took a snapshot of the cells with a microscope every day (Methods). The resulting snapshots form time-lapse movies at 5.0 °C. From these movies, we classified the cells into one of the following events: "begins life" (the daughter cell right after the mother cell finishes cytokinesis), "start replicating" (the mother cell right after it forms a bud), "end replicating" (the mother cell right after the it cell finishes cytokinesis), "grows" (a cell that grew in size without replicating for the duration of the time-lapse movies of ~17 days) and "dies" (cells that die during the time-lapse movies). We determined the cell size for all cells in each event. Shown are the cell sizes of individual cells from populations with high initial density (~8,000 cells / mL) **(b)**, low initial density (~420 cells / mL) with 250 µM added GSH **(c)**, and low initial density (~420 cells / mL) without any GSH added **(d)**. (b) At high density, newborn cells are the smallest cells in the population (29.2 ± 0.5 µm^2), with cells increasing in size during cell replication (from 35.8 ± 2.1 µm^2 at bud formation to 40.8 ± 2.3 µm^2 at cytokinesis). Growing cells are on average 43.8 ± 3.1 µm^2. Finally, cells that die are the largest cells in the population (59.1 ± 1.6 µm^2). (c) Similarly, in populations that were incubated in growth medium to which we added 250 µM GSH, we found that newborn cells are the smallest cells in the population (at 28.7 ± 2.5 µm^2), with replicating cells increasing in size during replication (starting at 37.6 ± 1.3 µm^2 and ending with 38.3 ± 2.0 µm^2 at the end of division). Growing cells are on average 40.8 ± 3.7 µm^2. Finally, with added GSH, cell deaths occur at larger sizes (70.1 ± 1.3 µm^2) compared to cells without added GSH. (d) In low-density (non-growing) populations, the newborn cells are on average larger (47.6 ± 5.8 µm^2) than the average size at which the cells start replicating (37.9 ± 1.2 µm^2), end replicating (43.7 ± 0.4 µm^2), or are growing (36.8 ± 1.9 µm^2). Finally, cells that die are smaller (55.3 ± 5.0 µm^2) than cells in the growing populations. For all panels, the error bar shows the mean with s.e.m., having n = 3 biological replicates. Dots are data aggregated from the biological replicates.

Together, these results show that, in growing populations, newborn cells have on average the smallest size of the population. In contrast, in low-density (non-growing) populations without added GSH, the newborn cells are on average larger than the replicating cells in the population. Moreover, these results show that the cell size monotonically increases from a cell's birth to its death, since the cell size increases for consecutive life events (i.e., a newborn cell either eventually replicates or will grow without replicating, and every cell eventually dies).

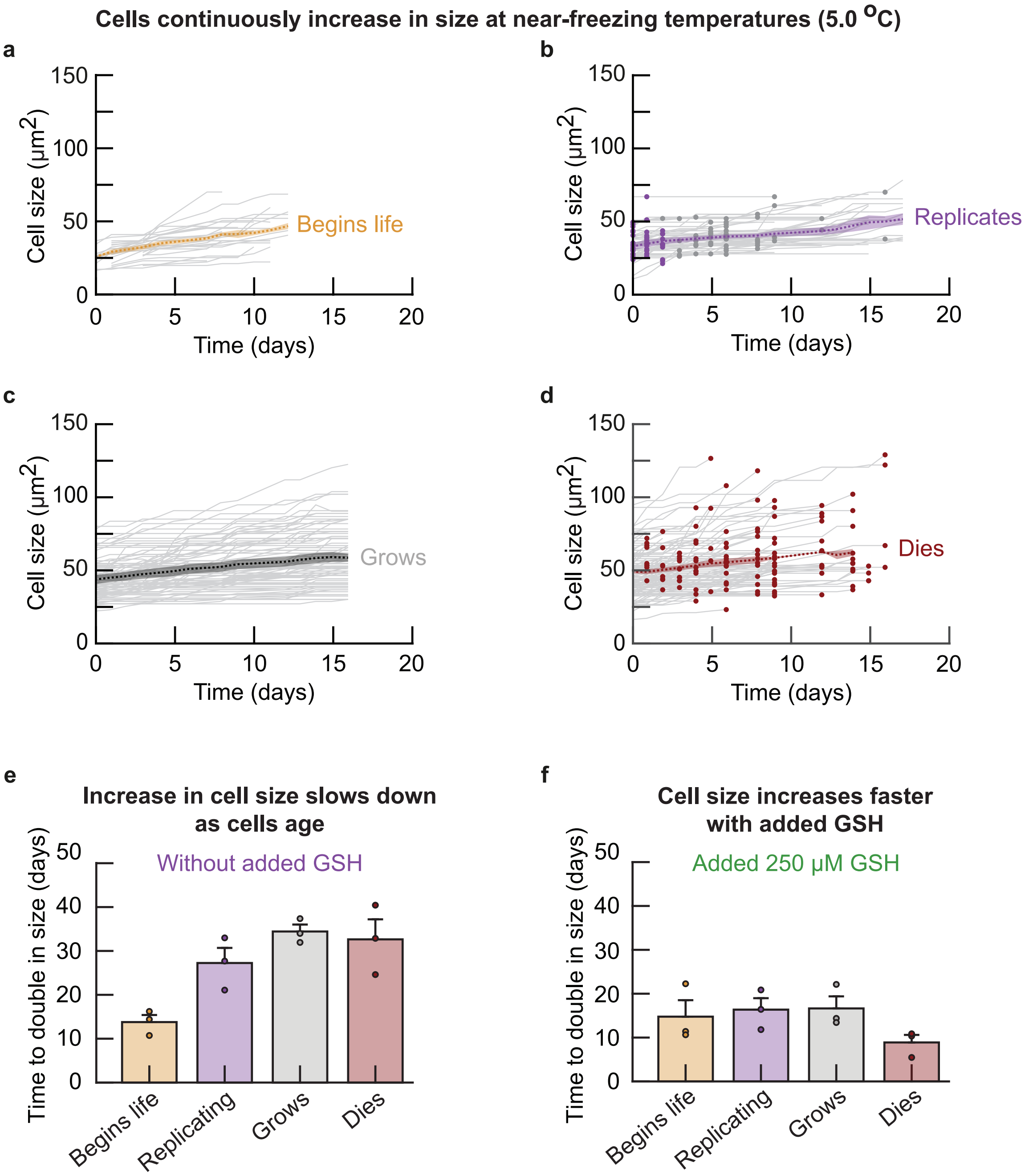

Supplementary Fig. 19: Cell size monotonically increases during all live events at 5 °C (Related to Figure 3c). Our data shows that the cell size monotonically increases at 5.0 °C starting with the smallest cell size at birth and ending with the largest cell size at death (including during cell replications, Supplementary Fig. 18). We used time-lapse microscopy to measure how the cell size changes over time in individual cells at 5.0 °C. **(a-d)** We used our time-lapse movies of cells in high-density populations (initially ∼8, 000 cells / mL) to track the cell size over time in single cells that were present at the start of the time-lapse movies. ***(caption continues)***

Supplementary Fig. 19 *(continued)*: To reconstruct cell size growth throughout every stage of a cell's life at near-freezing temperatures, we categorized all cells in the first time point of our movies (day "0") into one of four classes (Supplementary Fig. 18): "begins life" (newborn daughter cells just after cytokinesis), "replicates" (cells that replicated), "grows" (cells that grew without replicating for the entire duration of the time-lapse (~17 days,) and "dies" (cells that died). We assumed that the cells in the first time point of the movies were representative samples of populations at 5.0 °C, because the populations that we imaged were pre-incubated at 5.0 °C for two weeks before the first frame of the time-lapse. As an exception, since we did not know which cells were newborn cells when the time-lapse began, we used daughter cells that were newly born within a three-day period of our time-lapse movies. Shown here is the cell size over time for cells in each event, with newborn cells **(a)**, cells that replicated (i.e., when their bud began to grow) **(b)**, cells that grew without replicating **(c)**, and cells that died (almost always by bursting open **(d)**. Grey curves show the cell size over time for individual cells. Purple and grey dots in (b) indicate the time points at which bud formation (purple dots) and cytokinesis (grey dots) occurred respectively. Red dots in (d) indicate the cell size just before death. The dotted curves show the average size of cells each respective event for n = 3 biological replicates, and the shaded area represents the s.e.m. of the average cell size of each biological replicate. Together, (a-d) show that almost all cells continuously increase in size at 5.0 °C for the entire duration of the time-lapse movies. Given that a newborn cell will either eventually replicate or will grow without replicating and that every cell will eventually die, these data together show that cells continuously increase in their size throughout their life at 5.0 °C. **(e-f)** From the measurements in (a-d), we determined the time that cells take to double in size without added GSH **(e)** and with 250 μM added GSH **(f)**. Shown here is the time taken to double in size for cells right after cytokinesis (yellow bars), cells that begin to replicate (purple bars), cells that grow without replicating (grey bars), and cells just before they die (red bars). These measurements show that, without added GSH, cells take increasingly more time to double in size. This time is the shortest for a newborn cells (13.8 ± 1.6 days) and increases in the following order: cells that start to replicate (27.3 ± 3.4 days), cells that are growing without replicating (34.4 ± 1.6 days), and cells that are about to die (32.6 ± 4.6 days). In contrast, with added GSH, cells take approximately constant time to double in size for newborn cells (14.8 ± 3.8 days), cells that begin to replicate (16.4 ± 2.6 days), and cells that are growing without replicating (16.6 ± 2.8 days). Lastly, cells that die take the shortest time to double in size with 250 μM added GSH (8.9 ± 1.7 days). Dots show the average time for each biological replicate. Error bars show the mean with s.e.m. of n = 3 biological replicates. Together, (e-f) show that cells double in size more slowly as they (chronologically) age, and that adding GSH accelerates cell growth.

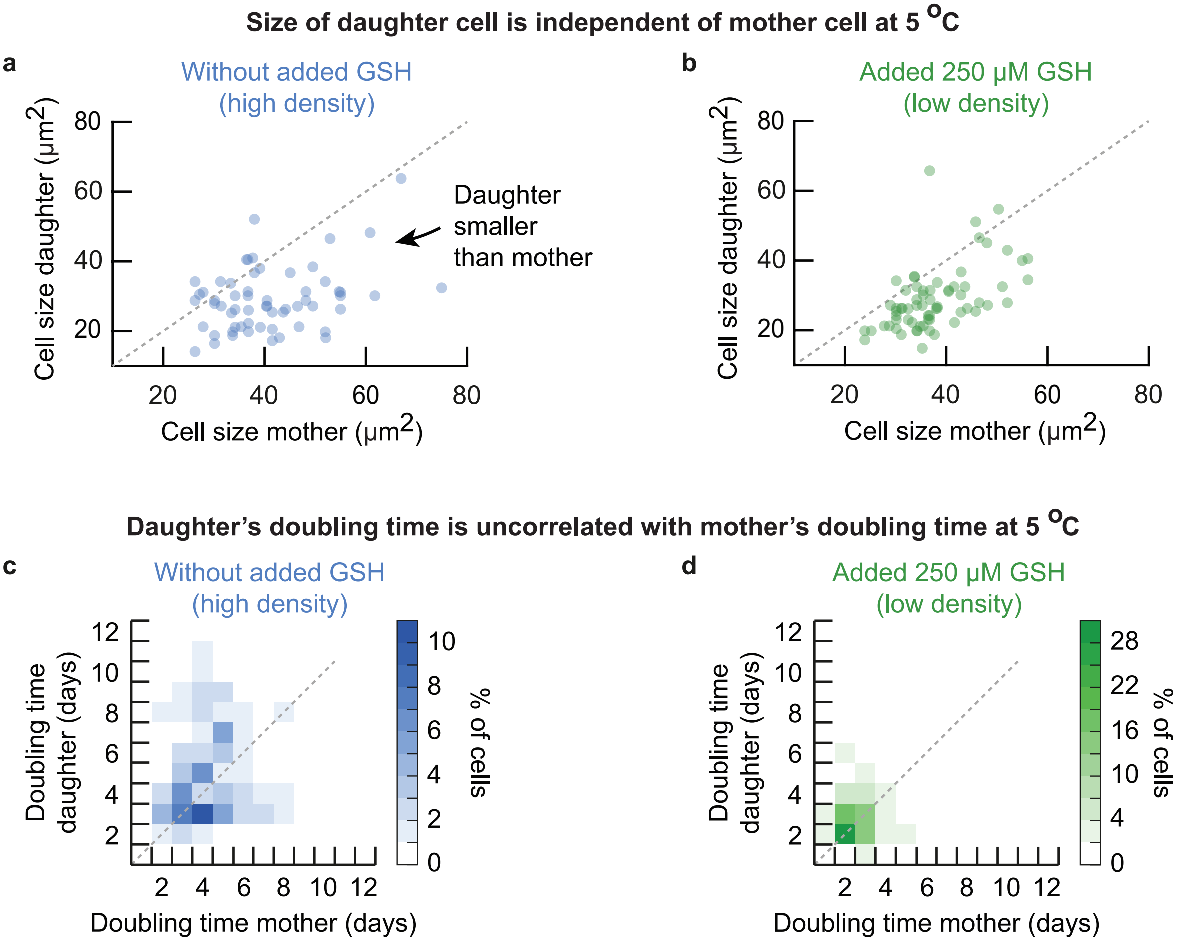

Supplementary Fig. 20: The size and doubling time of a newborn daughter cell are uncorrelated with its mother at 5 °C (Related to Figure 3d). Measuring how the cell size and doubling time of newborn cells depend on the size and doubling time of their mother cell. We grew populations of wild-type cells for two weeks at 5.0 °C and then began making time-lapse movies of these cells with our wide-field microscope (Methods). From these movies, we determined the cell size and doubling time of each mother-daughter pair. **(a-b)** Size of newborn daughter cells (directly after cytokinesis) compared to the cell size of the mother cell at the time she began to give birth to the daughter (i.e., when her bud started growing). Shown here are cells from populations without added GSH **(a)** or with 250 μM added GSH **(b)**. Grey diagonal line indicates locations where the mother and her newborn daughter having the same size. These graphs (a-b) show that the size of daughter cells poorly correlates with the size of their mother cells regardless of the presence of extracellular GSH. Moreover, newly born daughter cells are smaller than their mother cells as seen by the data points mostly lying below the diagonal line. **(c-d)** Doubling time of daughter cells as a function of the doubling time of their mothers in populations without added GSH **(c)** or with 250 μM of added GSH **(d)**. ***(caption continues)***

Supplementary Fig. 20 ***(continued)***: Heatmaps show doubling times binned into 1-day intervals. Data from the same time-lapse movies as in (a-b). Grey diagonal line indicates locations where the mother and daughter cells have the same doubling times. The doubling times of mother and daughter cells are poorly correlated. Without added GSH the doubling time of the daughter cell varies widely from two to over ten days, whereas the doubling time of mother and daughter cells are preserved at $\sim$2.5 days with 250 μM added GSH. Together, (a-d) show that both the cell size and doubling time of a mother cell are unrelated to the cell size and doubling time of its daughter cell, with the daughter cell usually being smaller than the mother cell (also see Supplementary Fig. 18). These results show that the size and doubling time of the daughter cell does not depend on the traits of her mother. All panels show data aggregated from n = 3 biological replicates.

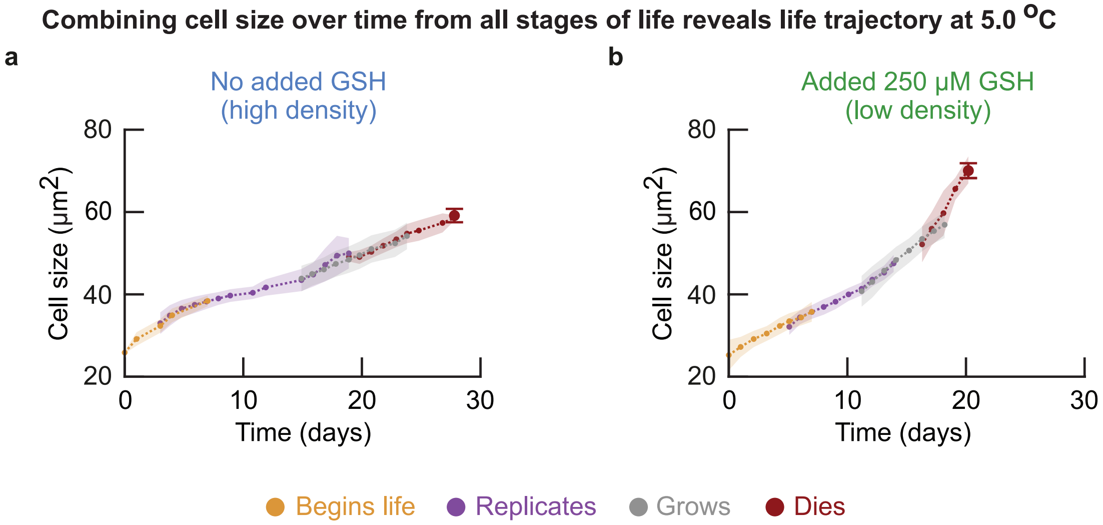

Supplementary Fig. 21: Continuous cell size growth during all life events reveals "life trajectory" of cells at 5 °C (Related to Figure 3d). We established that cells continuously increase in size throughout each life event at 5.0 °C (Supplementary Fig. 19). We also found that the cell size increases with consecutive life events (cells in "begins life" are smaller than cells in "replicates", followed by cells in "grows" and "dies", Supplementary Fig. 18). Moreover, since a newborn cell will either eventually replicate or will grow without replicating, and that every cell will eventually die, there exists a temporal order to these life events. We therefore used the cell size over time for each event to reconstruct the life of cells at 5.0 °C as function of cell size, by stitching together the cell size curves for each of the consecutive events based on the order of the average cell size (Supplementary Fig. 18): "begins life", "replicates", "grows" and "dies". Specifically, we connected each of the curves with its preceding curve at the time that the cell size at the respective endpoints overlapped most. Shown here are stitched curves for cell populations incubated **(a)** without any GSH supplemented (from n = 3 biological replicate populations; points represent means and shades represent s.e.m.); or **(b)** with 250 μM of GSH supplemented (from n = 3 biological replicate populations; points represent means and shades represent s.e.m.). Each curve overlaps with at least three data points with neighboring curves. The colors of each curve indicates the separate life events, showing a cell that begins life (yellow curve), replicates (purple curve), grows without replicating (grey curve), and dies (red curve). Large red dot shows average cell size just before cell death (from n = 3 biological replicate populations, mean with error bar as s.e.m.). With the life trajectories as described here, we constructed the cell's timelines. Specifically, the time window for replications (12 days without added GSH, 18 days with added GSH) transitions into the time window for growth without replicating at the cell size where the probability of replicating decreases below the probability of growing or dying (Fig. 3c). ***(caption continues)***

Supplementary Fig. 21 *(continued)*: The average replicative age (2 divisions without added GSH, 6 divisions with added GSH) is the time window for replications divided by the average single-cell doubling time (Supplementary Fig. 12). The average chronological age (28 days without added GSH, 20 days with added GSH) was determined from the life trajectory and the average cell size just before death (Supplementary Fig. 18).

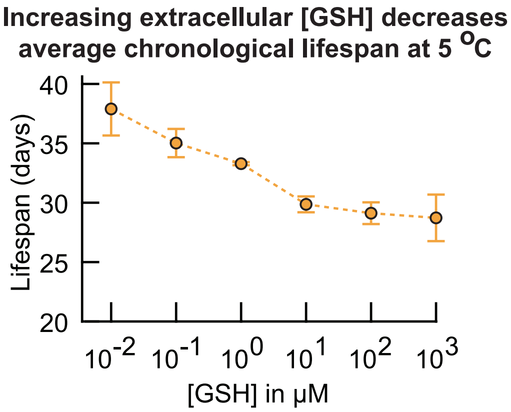

Supplementary Fig. 22: Average chronological lifespan decreases as the added GSH concentration increases at 5 °C (Related to Figure 3d). Cell's average chronological lifespan (average chronological age at death) as a function of added extracellular GSH concentration. We incubated wild-type cell populations at 5.0 °C with various amounts of GSH added to the growth medium (all initially at ∼5, 680 cells / mL). We took an aliquot of each liquid culture every week and measured the number of alive and dead cells using propidium iodide (Methods). We then fitted these measurements to the same growth model as before to determine the average chronological lifespan of cells (Supplementary Fig. 5; Supplementary Theory). The average chronological lifespan of cells at 5.0 °C decreases from ∼37 days without added GSH to ∼30 days with 100 μM added extracellular GSH. Extracellular GSH therefore decreases the chronological lifespan of cells at near-freezing temperatures. Error bars represent the mean with s.e.m., having $n = 4$ biological replicates per data point.

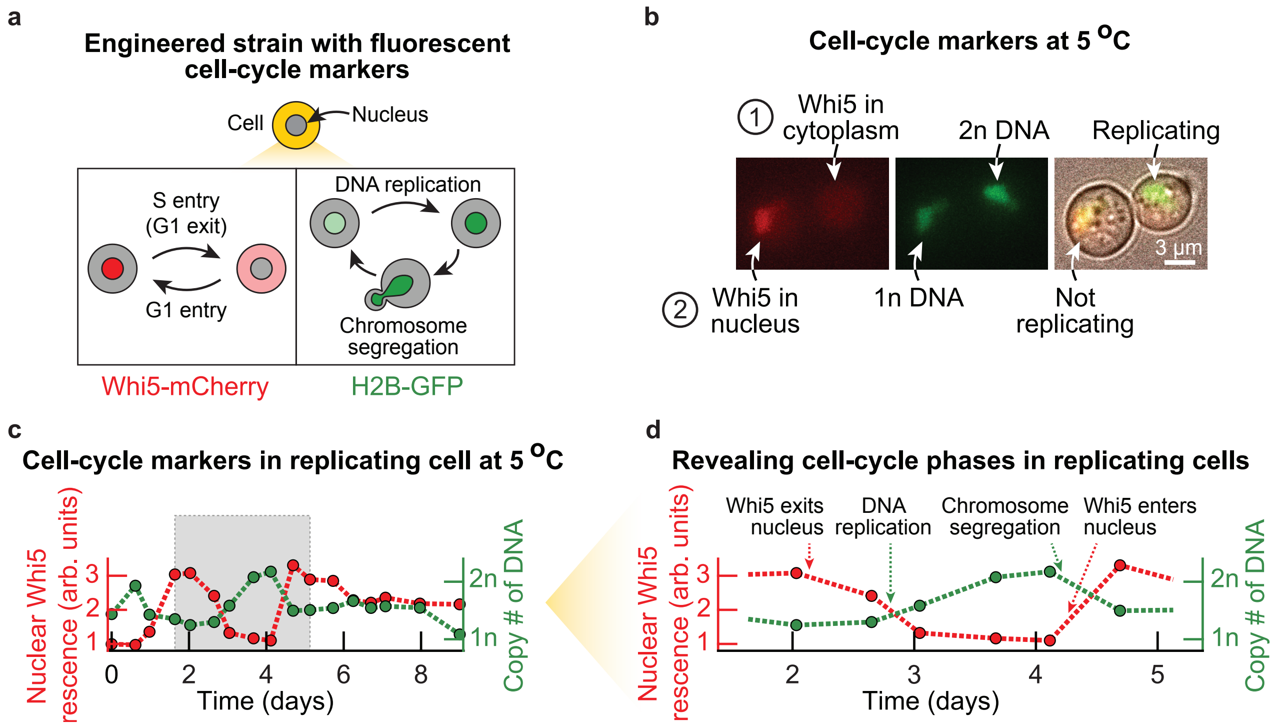

Supplementary Fig. 23: Time-lapse microscopy on engineered cells with fluorescent reporters for DNA replication (H2B histone fused to GFP) and G1-to-S transition (Whi5 protein fused to mCherry) reveals cell-cycle phases at 5 °C (Related to Figure 4). (a) Schematic of the engineered strain used for identifying when key cell-cycle phases occur in single cells at 5.0 °C. We engineered yeast to have the G1 transcriptional repressor Whi5 protein fused to mCherry protein (Whi5-mCherry), and the histone protein, H2B (Htb2), fused to GFP (H2B-GFP) (Methods). Both Whi5 and H2B are well-known cell-cycle markers that indicate G1 phase (Whi5 translocates to the nucleus [9, 10, 11, 12, 13, 14]) and the S-G2-M phases (the amount of H2B is proportional to the amount of DNA [13, 15, 16]) respectively. We used this engineered strain to track the cell cycle at 5.0 °C. **(b)** Microscopy snapshots of the engineered strain. Fluorescence of Whi5-mCherry (red, left image) and H2B-GFP (green, middle) are shown for two representative cells at 5.0 °C. Also shown is the composite image that includes the brightfield image (right). Here, one cell is replicating which we can tell, aside from the brightfield image, by finding almost all of the Whi5-mCherry in the cytoplasm and a bright nucleus with H2B-GFP (contains 2n DNA, labelled ”1” in the top row). In the same picture, we see that the other cell is not dividing, which we can also tell by finding almost all the Whi5-mCherry to be localized in the nucleus and a dim nucleus with H2B-GFP (contains 1n DNA, labelled cell ”2” in the bottom row). The dividing cell has a bud. Scale bar is 3 μm. Images are representative of n = 3 biological replicate populations. The exact numbers of cells that we analyzed for each population and condition are provided in the caption for Supplementary Figures 24 and 25. ***(caption continues)***

Supplementary Fig. 23 *(continued)*: (c) Nuclear Whi5-mCherry and H2B-GFP over time shown for a replicating cell at 5.0 °C. This cell is from a population that was pre-incubated for two weeks at 5.0 °C before starting the time-lapse, after which we tracked cells in these populations with a microscope (initially at $\sim$6, 250 cells / mL). We took aliquots of our liquid cultures that we transferred to an imaging plate. We then kept this plate at 5.0 °C and imaged the cells every $\sim$12 hours (Methods). Finally, we used this microscopy time-lapse to quantify the amount of nuclear Whi5-mCherry (left y-axis, red) and H2B-GFP (righ y-axis, green) over time. The grey area indicates a full cell-cycle, starting in G1 (Whi5-mCherry located in the nucleus). Specifically, we used the fluorescence of H2B-GFP to locate the nucleus and quantify the amount of DNA in the cells. We first located the nucleus by segmenting the GFP fluorescence of each cell using a threshold GFP fluorescence that we kept fixed for all cells and all time points when analysing this engineered strain. The nucleus was then the group of pixels whose fluorescences exceeded this threshold. To determine the copy number of DNA in a cell, we determined the total GFP fluorescence within the cell's nucleus. We subsequently rescaled the nuclear GFP between the average minimum and maximum GFP fluorescence that we observed for replicating cells. Thus, the amount of nuclear H2B was rescaled to a scale between "1n DNA" (average GFP fluorescence in the nucleus of replicating cells in G1) and "2n DNA" (average GFP fluorescence in the nucleus of replicating cells in G2). Finally, we used the fluorescence of Whi5-mCherry to quantify the relative amount of nuclear Whi5. To do so, we determined the average mCherry fluorescence in the nucleus and cytoplasm of each cell. The amount of nuclear Whi5 was then given by the ratio of measured nuclear and cytoplasmic mCherry fluorescence. **(d)** Nuclear Whi5 and H2B mark the cell-cycle phases of replicating cells. Shown is a cell during one cell cycle (from the grey box of the replicating cell in (c)). Dotted arrows indicate the end of G1 (Whi5-mCherry exits the nucleus), the S phase (the amount of H2B-GFP increases during replication of DNA), the M phase (H2B-GFP decreases during chromosome segregation) and the start of G1 (Whi5-mCherry begins to enter the nucleus).

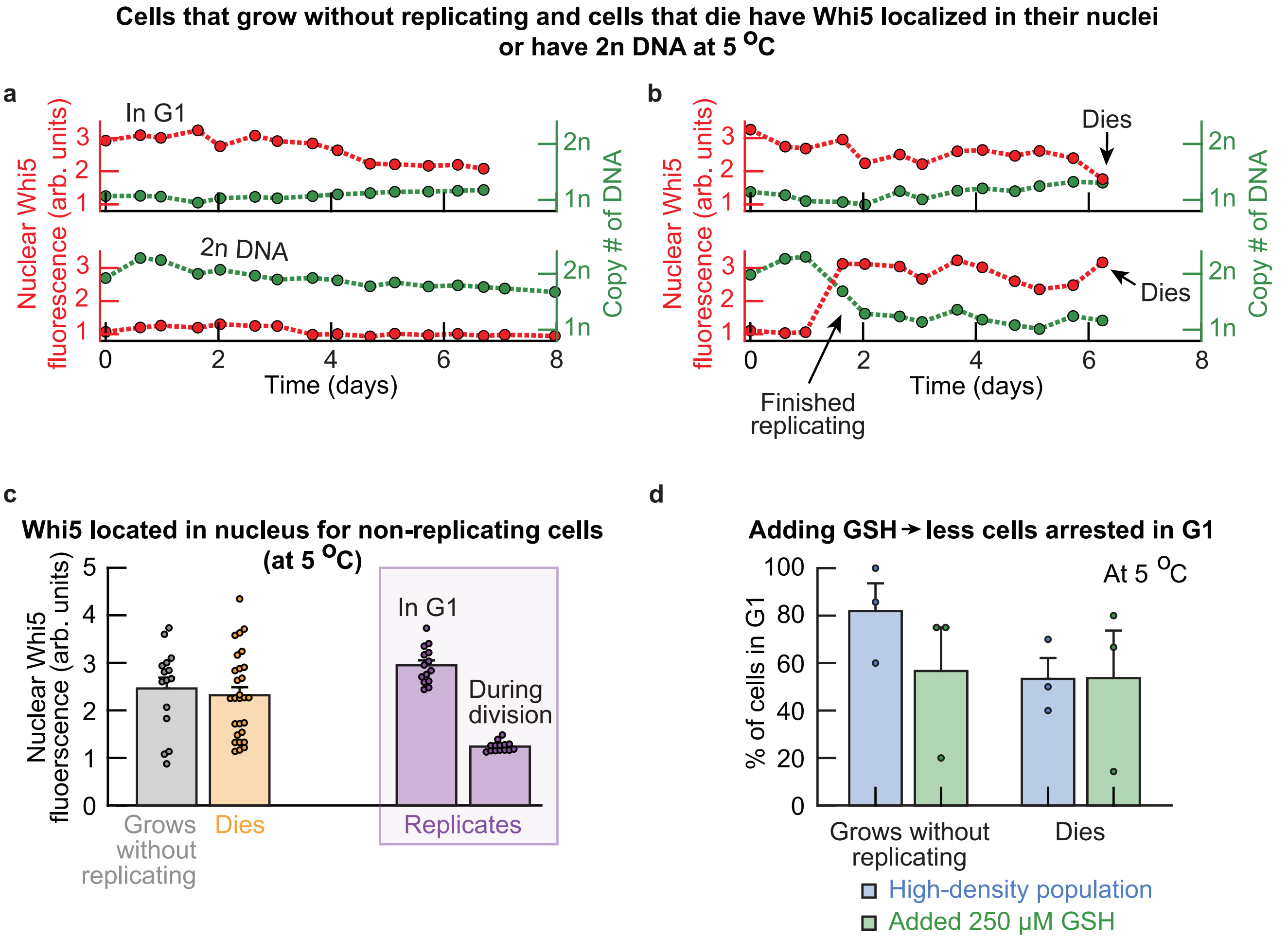

Supplementary Fig. 24: Growing cells that never replicate until dying are arrested in G1 phase at 5 °C (Related to Figure 4c). (a-b) Nuclear Whi5-mCherry and H2B-GFP over time in cells that grow without replicating and in dying cells at 5.0 °C (also see Supplementary Fig. 23). Shown are cells that did not replicate and stayed in G1 or G2 phase **(a)**, and cells just before they die **(b)**. The amount of nuclear Whi5-mCherry and the amount of DNA remained constant during many days of incubation in the cells that did not replicate. In the top graph of **(a)**, the red and green curves terminate after ∼ 7 days not because these cells die but because these cells became obscured by other cells that came into the same field of view in our time-lapse imaging. **(c)** Cells that grow without replicating and cells that die often have Whi5 located in the nucleus. Shown is the average amount of nuclear Whi5-mCherry in cells that grew without replicating (grey bar), in cells that died (orange bar) and for cells that replicated (purple box) when in G1 phase (left purple bar) or during cell division (S-G2-M phases, right purple bar). The data suggests that most cells that grow without replicating stay in G1 (their Whi5-mCherry always stays in the nucleus, in contrast to the cells in G1 that do replicate). ***(caption continues)***

Supplementary Fig. 24 ***(continued)***:

The average amount of nuclear Whi5-mCherry in cells that grew without replicating was determined by averaging the amount of nuclear Whi5-mCherry for the entire duration of the time-lapse. The average amount of nuclear Whi5-mCherry in cells that died represents the amount of nuclear Whi5-mCherry averaged over the last three frames ($\sim$36 hours) before the cell dies. The average amount of nuclear Whi5-mCherry for cells that replicated and were in G1 and for cells that were dividing, were determined as follows: we determined when Whi5-mCherry was located in the cytoplasm or the nucleus. We then averaged the amount of nuclear Whi5-mCherry for each cell while the cell is either in G1 or S-G2-M. Dots show raw data (n = 14 replicating cells, n = 15 growing, non-replicating cells, and n = 28 dying cells). Bars show the mean with s.e.m. of all cells. Note that the two purple bars represent the same population of duplicating cells. The left purple bar shows the average nuclear whi5 for duplicating cells when these cells are in G1. The right purple bar shows the average nuclear whi5 for duplicating cells when these cells are in S/M phase. **(d)** Cells that grow without replicating and cells that die mostly remain in G1. Shown here are the percentages of cells in G1 phase in growing populations of high-density (blue bars) and low-density populations supplemented with 250 μM GSH (green bars). This plot shows that at least 60% of non-replicating cells remained in G1 until they die. Extracellular GSH decreased the percentage of cells that remained in G1. Whether a cell is in G1 or not was determined with the average amount of nuclear Whi5-mCherry (in (c)) and the amount of DNA in the cell (a cell was in G1 when the amount of nuclear Whi5-mCherry was above 1.66 and when the amount of DNA was below 2n). Bars show the mean with s.e.m., having n = 3 biological replicates per condition. Dots show average percentage in each replicate.

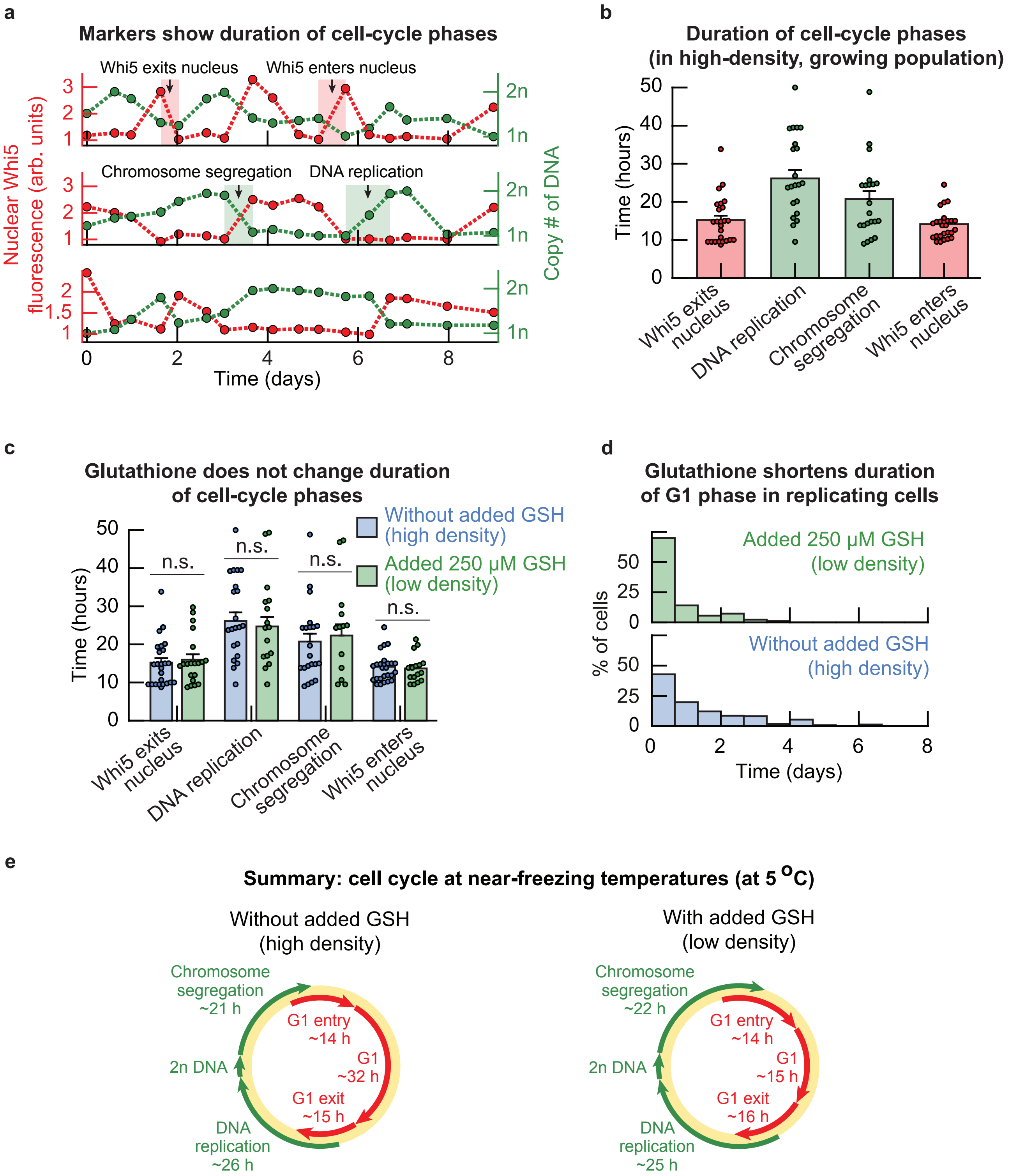

a
Markers show duration of cell-cycle phases
Whi5 exits nucleus
Whi5 enters nucleus
Chromosome segregation
DNA replication
Nuclear Whi5 fluorescence (arb. units)
Copy # of DNA
Time (days)
b
Duration of cell-cycle phases (in high-density, growing population)
Time (hours)
Whi5 exits nucleus
DNA replication
Chromosome segregation
Whi5 enters nucleus
c
Glutathione does not change duration of cell-cycle phases
Without added GSH (high density)
Added 250 μM GSH (low density)
n.s.
Time (hours)
Whi5 exits nucleus
DNA replication
Chromosome segregation
Whi5 enters nucleus
d
Glutathione shortens duration of G1 phase in replicating cells
Added 250 μM GSH (low density)
Without added GSH (high density)
% of cells
Time (days)
e
Summary: cell cycle at near-freezing temperatures (at 5 °C)
Without added GSH (high density)
Chromosome segregation ~21 h
G1 entry ~14 h
G1 ~32 h
G1 exit ~15 h
2n DNA
DNA replication ~26 h
With added GSH (low density)
Chromosome segregation ~22 h
G1 entry ~14 h
G1 ~15 h
G1 exit ~16 h
2n DNA
DNA replication ~25 h

Supplementary Fig. 25: Glutathione shortens duration of G1 phase, but not S-G2-M phases, at 5 °C (Related to Figure 4c-d). **(a)** Nuclear Whi5-mCherry and H2B-GFP over time in replicating cells that were incubated at 5.0 °C (Supplementary Fig. 23). Red shaded areas indicate examples of the duration of Whi5-mCherry exiting or entering the nucleus. Green shaded areas indicate examples of the duration of DNA replication or chromosome segregation. **(b)** Using time-lapse movies that examined the amounts of Whi5-mCherry and H2B-GFP in single cells, we determined the duration of cell-cycle events in replicating cells from high-density, growing populations (following the methodology in (a)). Shown here are the durations of Whi5-mCherry exiting the nucleus (15.2 ± 1.1 hours, red bar), DNA replication (26.1 ± 2.2 hours, green bar), chromosome segregation (20.8 ± 2.1 hours, green bar), and Whi5-mCherry entering the nucleus (14.1 ± 0.8 hours, red bar). Since the time-lapse movies consists of snapshots taken every ∼12 hours, this ∼12 hours is a lower bound on the true duration of each event. Dots show raw data pooled from n = 3 biological replicate populations, having 28 cells for the location of Whi5-mCherry, 23 cells with DNA replication and 24 cells with DNA segregation. Bars show the mean with s.e.m. of all cells. **(c)** Duration of cell-cycle events in cells from low-density populations that were incubated with 250 µM of GSH (green bars). Blue bars show the same data as in (b) as a comparison. Shown here are, all with the added GSH, Whi5-mCherry exiting the nucleus (16.0 ± 1.4 hours), DNA replication (24.7 ± 2.5 hours), chromosome segregation (22.3 ± 2.9 hours), and Whi5-mCherry entering the nucleus (13.7 ± 0.9 hours). There is no reason to assume that the duration of any cell-cycle event changes upon addition of extracellular GSH (p-values are: p = 0.69 for Whi5-mCherry exiting the nucleus; p = 0.69 for DNA replication; p = 0.66 for chromosome segregation; and p = 0.72 for Whi5-mCherry entering the nucleus; 'n.s.' means 'not significant in these bar plots). We determined these p-values with the two-sides Student's t-test with unequal variances and at a 95%-confidence level. Multiple testing/comparison was not applicable here. Green dots show raw data from n = 3 biological replicate populations, having the following sample sizes: 16 cells for Whi5-mCherry entry; 20 cells for Whi5-mCherry exit of the nucleus;17 cells with DNA replication; and 16 cells with DNA segregation. Blue dots are as in (b). Bars represent the mean of all cells and error bars show s.e.m. of all cells. **(d)** Histograms showing the durations of G1 phase in replicating cells from populations without added GSH (blue) and with 250 µM added GSH (green). The average duration of G1 was 32 ± 3.0 hours without added GSH, and 15.0 ± 1.5 hours with added GSH (median ∼24 hours without added GSH and ∼9 hours with added GSH). Thus, GSH shortens the duration of G1 in replicating cells at near-freezing temperatures. With added GSH, the majority of G1 phases in replicating cells take 12 hours or less. In contrast, without added GSH, the G1 phase in replicating cells takes up to 6 days. Each histogram is an average histogram and is representative of n = 3 biological replicate populations. **(e)** Summarizing (b-d). Shown are the durations of the cell-cycle phases in replicating cells from populations without added GSH (left) and with added GSH (right). ***(caption continues)***

Supplementary Fig. 25 ***(continued)*****:** The length of the arrows is proportional to the duration of each phase. Red arrows represent Whi5 (indicating events associated to G1), green arrows represent H2B (indicating events associated to S-G2-M). Together, (b-d) show that adding GSH to the growth medium shortens the duration of G1 in replicating cells, while the duration of other cell-cycle remains unchanged upon addition of GSH.

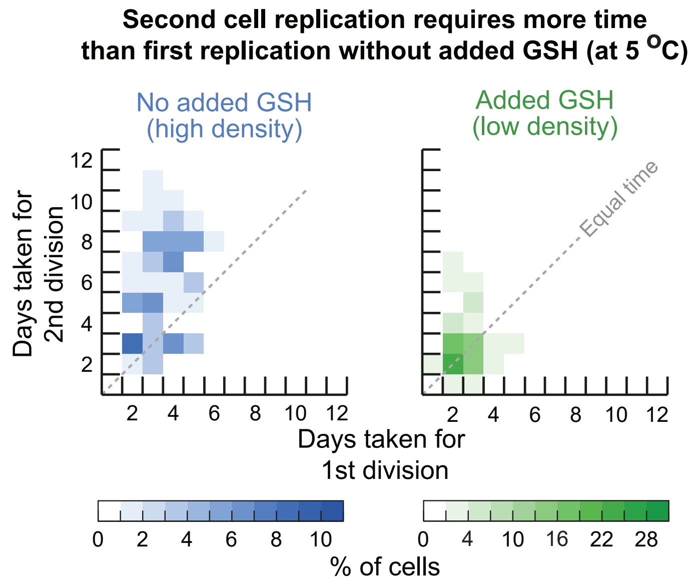

Supplementary Fig. 26: Consecutive divisions in individual cells slow down at 5 °C (Related to Figure 4d). We used single-cell time-lapse movies at 5.0 °C (Supplementary Fig. 12) to measure the doubling time of cells that replicated multiple times. Heatmaps show the durations of consecutive replications of individual cells at 5.0 °C without added GSH (blue heatmap, left) and with 250 μM added GSH (green heatmap, right). Shown is the number of days taken for a cell replication (y-axis) as function of the number of days taken for the preceding cell replication (x-axis) of each cell. Grey dotted diagonal indicates location where consecutive replications in the same cell have equal doubling times. Data is aggregated from n = 3 biological replicates. These measurements show that the doubling time of individual cells increases over time in populations without added GSH: the duration of a cell replication is on average larger (above the diagonal) than the duration of a preceding cell replication. With added GSH, the doubling time remains approximately constant for consecutive replications.

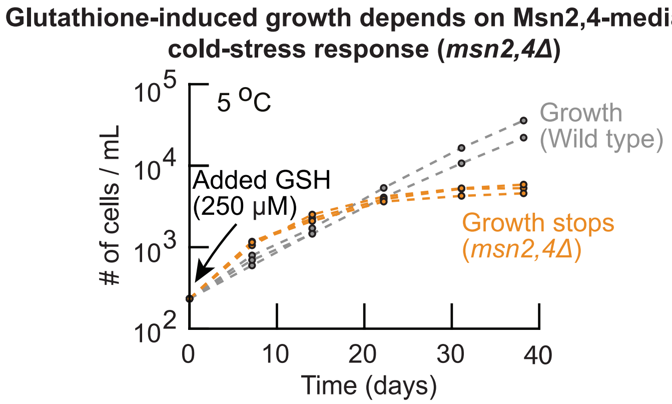

Supplementary Fig. 27: Glutathione-induced growth at near-freezing temperatures depends on Msn2,4-mediate cold-stress response (Related to Figure 4e). We constructed a mutant strain that lacks the major stress-response transcriptional activators, Msn2,4 (*msn2,4*Δ strain) [21, 22, 23]. Shown here is the population density over time for the *msn2,4*Δ strain at 5.0 °C with 250 μM added GSH (orange curves, initially ∼250 cells / mL). Grey curves show the wild-type strain for comparison (initially ∼250 cells / mL with 250 μM added GSH). The *msn2,4*Δ strain stops growing after two weeks of incubation with added GSH at 5.0 °C, whereas the wild-type strain grows exponentially over time. We already established that GSH removes intracellular ROS and thereby induces the wild-type population to grow (Fig. 2c). However, the *msn2,4*Δ population stops growing at 5.0 °C even with the added GSH. This means that yeasts incubated with extracellular GSH still experience a low-temperature stress, despite the added GSH reducing the intracellular ROS that inhibits cell replications. Together, our results suggest that glutathione-induced population growth at 5.0 °C depends on the Msn2,4 cold-stress response. All colors show n = 3 biological replicates.

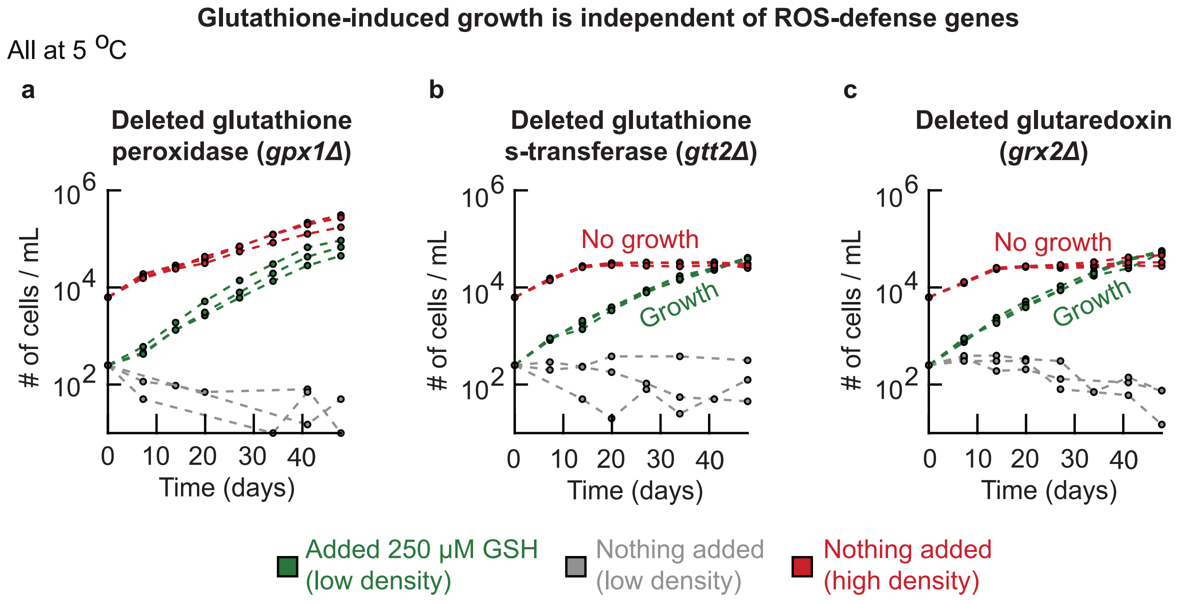

Supplementary Fig. 28: Glutathione induces population growth at 5 °C independent of ROS-defense genes (Related to Figure 4e). All at 5.0 °C. **(a-c)** We constructed mutants that are each lacking a gene ("ROS defense" gene) which protects the cells from oxidative stress, maintains redox state, and work with glutathione [17, 18, 19, 20]. Specifically, one strain lacks *GPX1* which encodes a glutathione peroxidase (*gpx1*Δ strain). Another strain lacks *GTT2* which encodes a glutathione s-transferase (*gtt2*Δ strain). Another strain lacks *GRX2* which encodes a glutaredoxin (*grx2*Δ strain). Shown here are the population densities over time for the *gpx1*Δ strain **(a)**, the *gtt2*Δ strain **(b)**, and the *grx2*Δ strain **(c)**. Populations of each strain started with high density (∼6, 250 cells / mL, red curves), low density (∼250 cells / mL, grey curves), or low density with 250 µM added GSH (∼250 cells / mL, green curves). Every mutant population with the added extracellular GSH grew. Surprisingly, the high-density populations of the *gtt2*Δ and *grx2*Δ strains did not sustain growth at 5.0 °C beyond an initial, transient growth of ∼10 that was due to the cells being transferred from 30 °C. In contrast, at the same high density, populations of the wild type (Fig. 1) and the *gpx1*Δ strain grew to the carrying capacity. Hence, *GTT2* and *GRX2* are necessary for sustaining population growth at 5.0 °C, while *GPX1* is not required. Finally, both *GTT2* and *GRX2* can be substituted with reduced extracellular GSH at 5.0 °C because every population with the added extracellular GSH grew. All colors for each strain show n = 3 biological replicates.

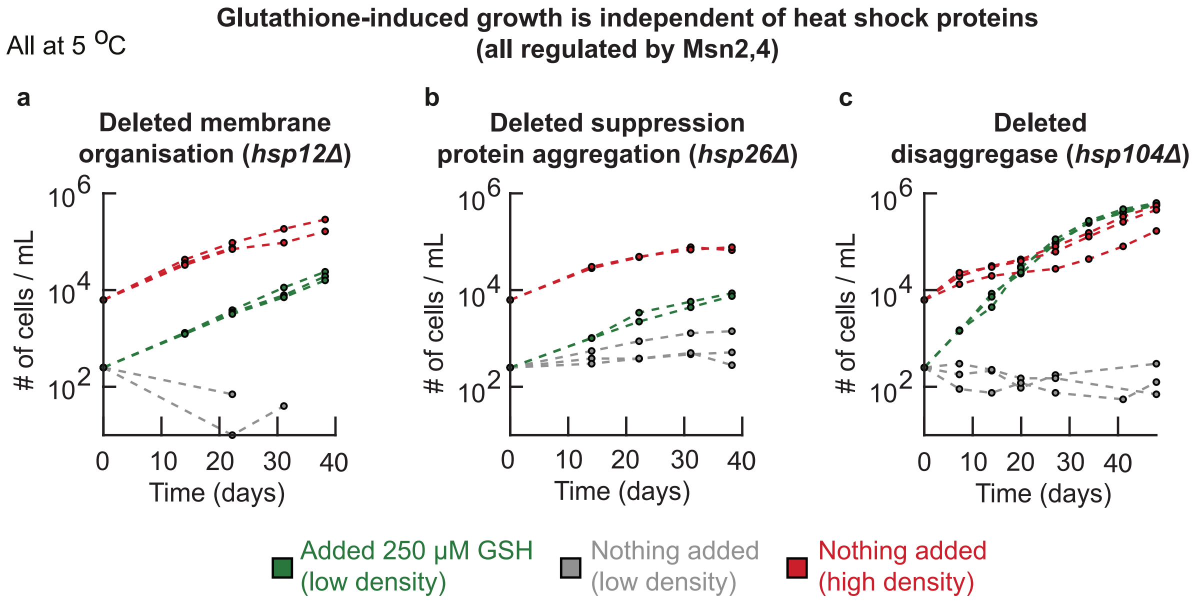

Supplementary Fig. 29: Msn2,4-regulated heat-shock proteins are not necessary for growth at 5 °C (Related to Figure 4e). All at 5.0 °C. **(a-c)** We constructed mutants that were lacking genes ("heat shock" genes) which are regulated by the Msn2,4 stress-response and highly expressed at low temperatures [2, 24, 25, 20, 17]. Specifically, one mutant strain lacks *HSP12* which encodes a membrane protein that helps in maintaining membrane organization (*hsp12*Δ strain). Another strain lacks *HSP26* which encodes a chaperone protein that suppresses aggregation of unfolded proteins (*hsp26*Δ strain). Another strain lacks *HSP104* which encodes a disaggregase (*hsp104*Δ strain). Shown here are the population densities over time for the *hsp12*Δ strain **(a)**, the *hsp26*Δ strain **(b)**, and the *hsp104*Δ strain **(c)**. Populations of each strain started with either a high density (∼6, 250 cells / mL, red curves), low density (∼250 cells / mL, grey curves), or a low density with 250 μM added GSH (green curves). Every high-density population grew for all heat shock mutants. Similarly, all populations with added extracellular GSH grew, with the *hsp104*Δ strain growing fastest and the *hsp26*Δ strain growing slowest. These growth experiments show that not all Msn2,4-regulated genes are necessary for growth, even though the Msn2,4 stress-response is required for cell replications at near-freezing temperatures (Supplementary Fig. 27). Each condition in all panels shows $n = 3$ biological replicates.

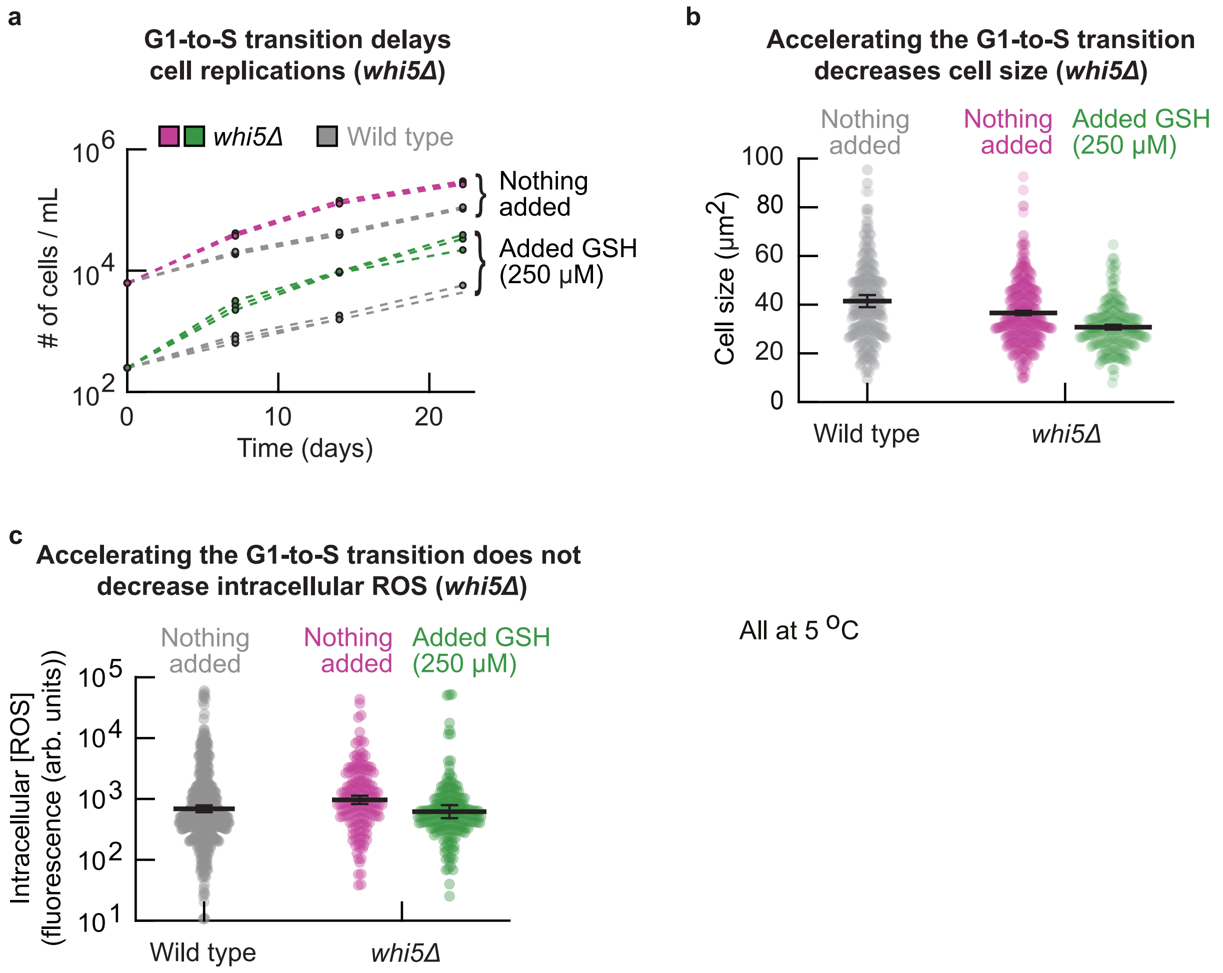

Supplementary Fig. 30: G1-to-S transition delays cell replication at 5 °C (Related to Figure 4e). **(a)** We constructed a mutant strain (*whi5*Δ strain) whose *WHI5* was knocked out and therefore has an accelerated G1-to-S transition at 30 °C [10]. Shown here are the population densities over time for the *whi5*Δ strain that was incubated at 5.0 °C either without added GSH (initially $\sim$6,250 cells / mL, pink curves) or with 250 µM added GSH (initially $\sim$250 cells / mL, green curves). Both conditions are compared with the wild-type strain (grey curves). The *whi5*Δ strain grows faster than the wild type at 5.0 °C (resulting in a 3.4 ± 0.1-fold difference in density after two weeks without added GSH, and a 5.7 ± 0.3-fold difference in density with added GSH after the same period of time). Thus, knocking out *WHI5* accelerates population growth, suggesting that the G1-to-S transition delays cell replications. Each color and initial density show n = 3 biological replicate populations. **(b)** Cell size of the *whi5*Δ strain. Shown here are the cell sizes of the *whi5*Δ strain in populations without added GSH (each pink dot represents one cell) or with 250 added µM GSH (each green dot represents one cell). Wild-type strain without added GSH is shown as a comparison (each grey dot represents one cell). Cell sizes were measured after two weeks of incubation at 5.0 °C. ***(caption continues)***

Supplementary Fig. 30 ***(continued)*****:** The *whi5*Δ strain has a smaller average cell size compared to the wild type, with adding extracellular GSH reducing the cell size further. This suggests that accelerating the G1-to-S transition decreases the cell size at 5.0 °C (cells spend less time in the G1 (growth) phase). For all three conditions, black data point represents mean with error bars showing s.e.m.; n = 3 biological replicate populations for each condition. **(c)** Intracellular ROS concentration in the *whi5*Δ strain after two weeks of incubation at 5.0 °C (Methods). Shown here are populations that were incubated either without added GSH (each pink dot represents one cell) or with 250 μM added GSH (each green dot represents one cell). Each grey dot represents one cell of the wild-type population without any added GSH, as a comparison. The *whi5*Δ strain does not have less intracellular ROS compared to the wild-type strain. For all three conditions, a black data point represents the mean with error bars showing s.e.m.; n = 3 biological replicate populations for each condition. Together, (a-c) show that accelerating the G1-to-S transition at 5.0 °C accelerates cell replication and decreases cell size. Researchers found similar effects at 30.0 °C [10, 26]. Simultaneously, accelerating the G1-to-S transition does not decrease intracellular ROS abundance. This suggests that enabling the G1-to-S transition (G1 exit) with abundant ROS enables cell replication. Moreover, these measurements suggest that cells do not accumulate intracellular ROS during G1, since shortening the G1 duration does not decrease the intracellular ROS concentration.

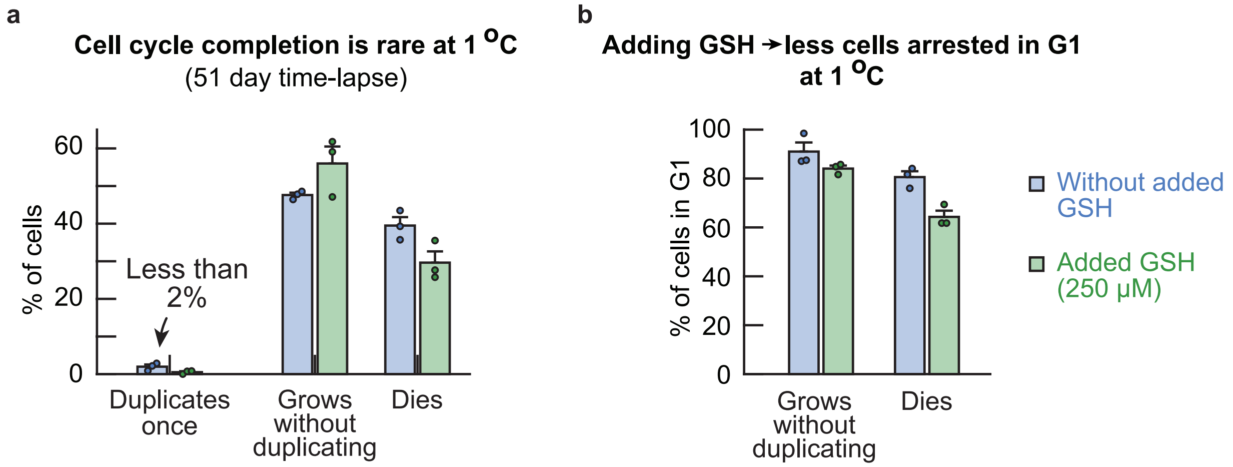

Supplementary Fig. 31: Cells are arrested in G1 and less than 2% of cells proceeds to replicate during a 51-day time-lapse at 1 °C (Related to Figure 5a). We incubated populations of the engineered strain with fluorescent cell-cycle markers (also see Supplementary Fig. 23) at 1.0 °C. After 10 days of incubation at 1.0 °C, we proceeded with tracking single cells with a microscope (initially ∼30, 000 cells per ml). In short, we took aliquots of the liquid cultures and transferred these to an imaging plate. We kept the plate at 1.0 °C and imaged the cells every ∼4 days (Methods). **(a)** Percentage of cells that replicates, grows and dies during the 51 day microscope time-lapse at 1.0 °C. Bars show populations without added GSH (blue bars) and with 250 μM added GSH (green bars). Less than 2% of cells replicated once (no cell replicated more than once). Bars represent mean with error bars showing s.e.m.; *n* = 3 biological replicate populations for each condition. **(b)** Percentages of cells that grow without replicating and cells that die while in G1 for the duration of the time-lapse. Almost all of the non-replicating cells are in G1. Similar to our measurements at 5.0 °C, we found that adding GSH decreases the percentage of cells that are in G1 (Supplementary Fig. 24). Bars represent mean with error bars showing s.e.m.; *n* = 3 biological replicate populations for each condition.

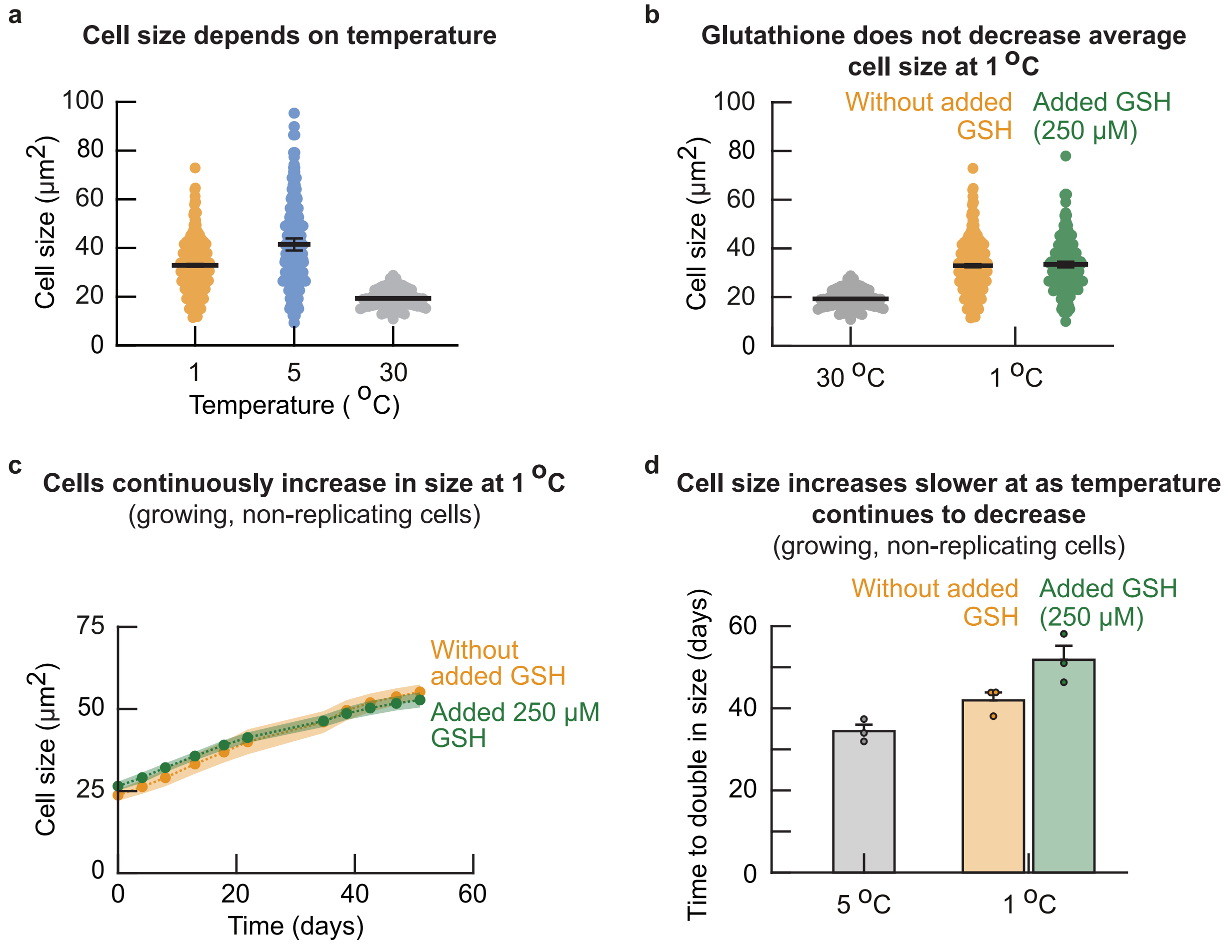

Supplementary Fig. 32: Cell size continuously increases at 1 °C, but slower than at 5 °C (Related to Figure 5a). (a) Cell size as function of temperature. We incubated wild-type cell populations for two weeks at 1.0 °C and then measured the cell sizes with a wide-field microscope. Shown here are the cell sizes (cross-sectional area) of individual cells from high-density populations without any added GSH at 1.0 °C (orange dots, initially ∼30, 000 cells / mL) and at 5.0 °C (blue dots; Supplementary Fig. 18). Grey dots show cells in a log-phase growth at 30.0 °C as a comparison. We find that cells at 1.0 °C are smaller in size compared to cells at 5.0 °C, but still larger in size than cells at 30.0 °C. **(b)** We also measured the cell sizes of individual cells with 250 μM added GSH after two weeks of incubation at 1.0 °C. Shown are the cell sizes of individual cells at 1.0 °C from high-density populations without any added GSH (orange dots, see (a)) and of cells from high-density populations with 250 μM added GSH (green dots). Data from 30 °C is as in (a). Here we find that cells are on average the same size with and without added GSH. This is in contrast with 5.0 °C where adding GSH decreases the average cell size. In (a-b), the error bars represent the mean with s.e.m., having n = 3 biological replicates. Dots show data aggregated from the biological replicates. ***(caption continues)***

Supplementary Fig. 32 ***(continued)*****:** **(c)** We used our time-lapse movies of cells in 1.0 °C to track the cell size over time for individual cells that were present at the start of the time-lapse movies (also see Supplementary Fig. 19). Shown here is the cell size over time for cells that did not replicate for the entire duration of the time-lapse (∼50 days). Cells were from populations that were incubated without added GSH (orange curve) or with 250 μM added GSH (green curve). All populations that we imaged were pre-incubated for two weeks at 1.0 °Cbefore the first frame of the time-lapse movies. The cell size continuously increases during the entire ∼50 day time-lapse. Dots show the mean with shades representing the s.e.m., having n = 3 biological replicate populations per condition. **(d)** From the measurements in (c), we determined the time that cells take to double in size. Shown here are the time taken to double in size for cells that grow without replicating at 1.0 °C, either without added GSH (orange bar) or with 250 μM added GSH (green bar). Grey bar shows the time taken to double in size for cells that grow without replicating at 5.0 °C without added GSH as a comparison (data from Supplementary Fig. 19). At 1.0 °C, cells take more time to double in size with added GSH (51.8 ± 3.4 days) compared to without added GSH (41.9 ± 1.9 days). Together, (a-d) show that cells continuously grow in size at 1 °C, but do so slower compared to 5 °C. Moreover, in contrast to 5 °C, GSH does not decrease the average cell size at 1 °C (also see Supplementary Figs. 18-19). Bars represent means with error bars showing s.e.m. for n = 3 biological replicate populations.

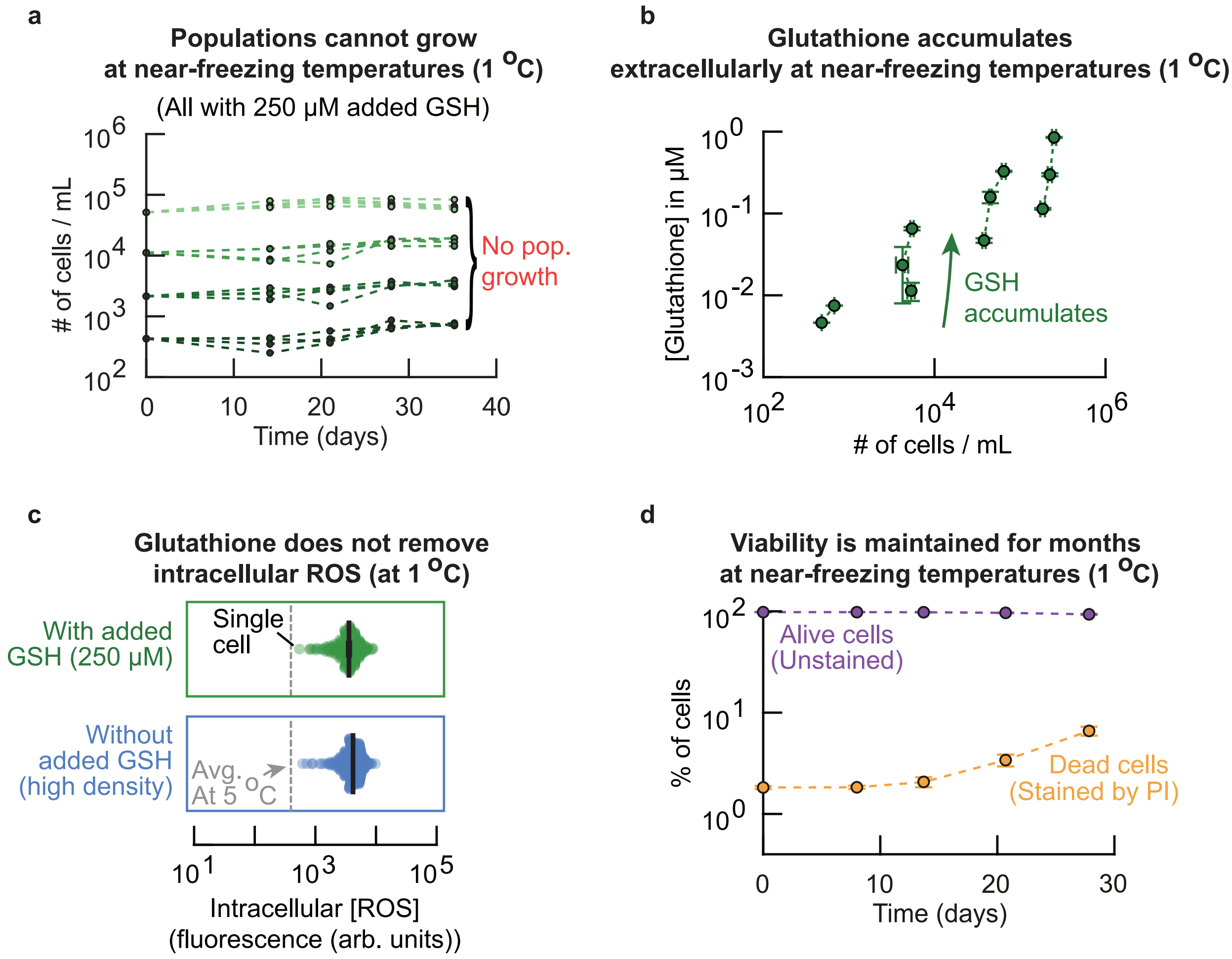

Supplementary Fig. 33: Glutathione still accumulates but does not remove intracellular ROS at 1 °C (Related to Figure 5). (a-c) Measuring population behavior of wild-type yeast at 1 °C. **(a)** Population-density over time for populations that were incubated with 250 μM added GSH at 1.0 °C. Colors show different initial population densities (highest density ∼52, 000 cells / mL, with 5-fold dilutions to lower densities). None of the populations increase appreciable in density (e.g., 10-fold) during the month long incubation. Thus, populations do not grow at 1.0 °C even with GSH added to the growth medium (also compare with Fig. 2c). Each color has n = 4 replicate populations. **(b)** We quantified the extracellular glutathione concentration for wild-type populations with an enzymatic assay kit (Methods). Shown are the concentrations of total extracellular glutathione as function of population-density after 1, 3 and 5 weeks of incubation at 1.0 °C (also see Fig. 2a). No glutathione was added to the growth medium (highest initial density ∼156, 000 cells / mL, with 5-fold serial dilutions for lower density populations). Green arrow indicates direction of time. The extracellular glutathione concentration increases over time for all populations. These measurements show that cells secrete and accumulate extracellular glutathione at 1.0 °C despite cell populations not growing (see (a)). ***(caption continues)***

Supplementary Fig. 33 ***(continued)***: Error bars are mean with s.e.m., having n = 3 biological replicates per data point. **(c)** Intracellular ROS concentration of individual cells as quantified by fluorescence of a oxidation-responsive dye for superoxides in mitochondria (Methods, also see Fig. 2b). Cells were stained after two weeks of incubation at 1.0 °C. Shown are intracellular ROS concentrations for individual cells from high-density populations (blue dots, initially $\sim$30,000 cells / mL) and populations incubated with 250 μM GSH (green dots, initially 2,000 cells / mL). Grey dotted line indicates the average intracellular ROS concentration in growing populations at 5.0 °C (from Fig. 2b). In contrast to 5.0 °C, the intracellular ROS concentrations at 1.0 °C are identical between populations with and without added GSH (also compare with Fig. 2b-d). Thus, adding extracellular GSH does not remove intracellular ROS at 1.0 °C. Error bars show the mean with s.e.m. of the average ROS level of each biological replicate (n = 3). Together, (a-c) show that cells still secrete glutathione to their extracellular environment at near-freezing temperatures. However, populations do not grow at 1.0 °C even with large amounts of GSH added to the growth medium. Moreover, extracellular GSH also does not remove intracellular ROS. These results are in contrast to yeast populations incubated at 5.0 °C, where extracellular GSH does remove intracellular ROS and induces population growth. **(d)** To test whether populations do not grow at 1.0 °C because the cells were dead, we determined the percentage of alive and dead cells over time in yeast populations incubated at 1.0 °C through a PI staining (Supplementary Fig. 4). Shown is the percentage of alive cells (purple points) and percentage of dead cells (orange points) for populations with a high density (initially $\sim$156,000 cells / mL). The percentage of dead cells in the population increases over time, reaching $\sim$10 % after 4 weeks of incubation at 1 °C. These measurements show that populations remain alive during months-long incubation at 1.0 °C. This result suggests that the reason populations do not grow at 1.0 °C is not because the cells were dead. Error bars show mean with s.e.m., with n = 4 replicate populations per data point.

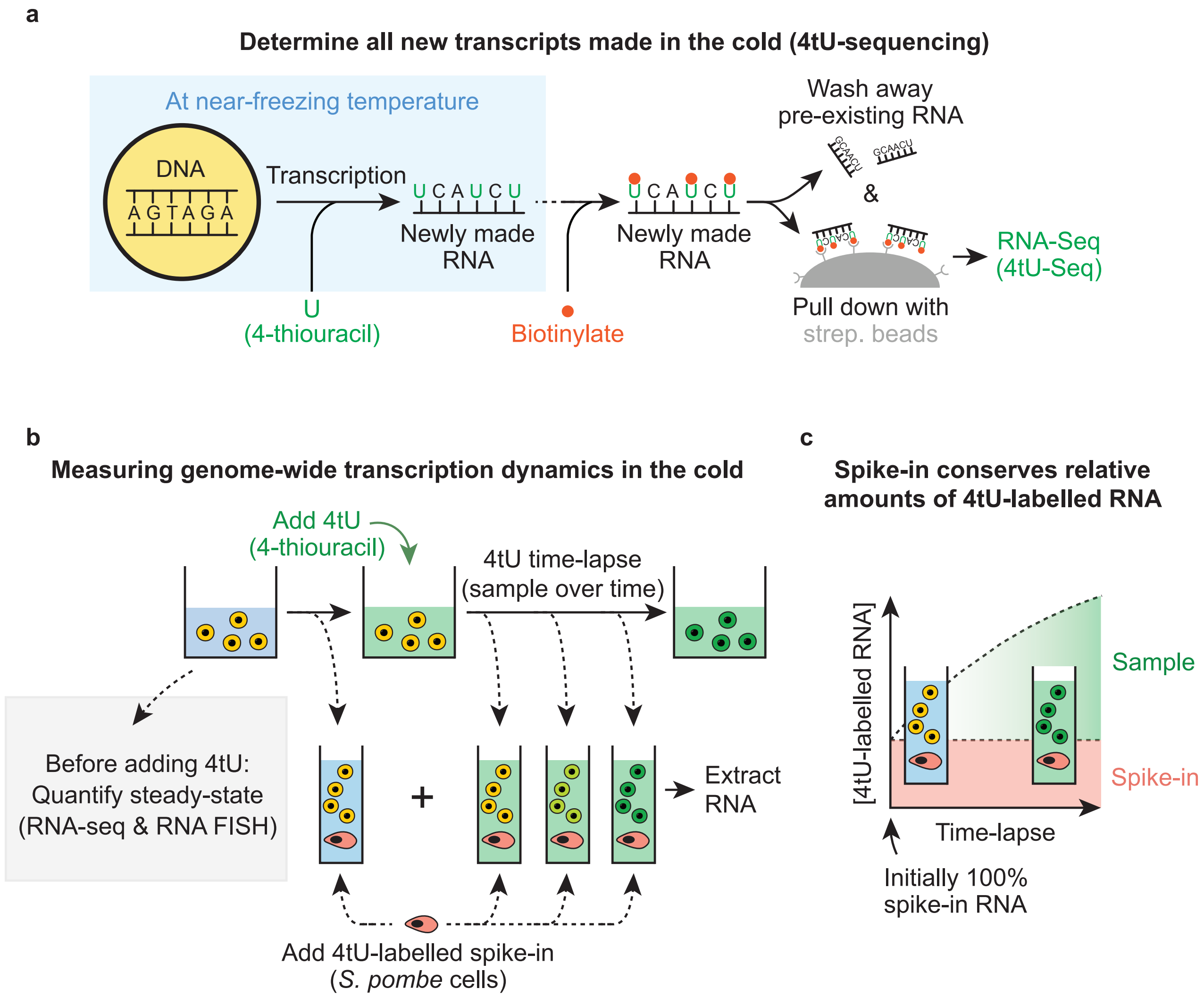

Supplementary Fig. 34: Measuring the genome-wide transcription dynamics at near-freezing temperatures (Related to Figure 5b). (a) Schematic demonstrating the experimental procedure for performing 4tU-sequencing on newly-made transcripts in populations of *S. cerevisiae* [27, 28, 29]. A synthetic uracil analog (4-thiouracil, 4tU) is added to the growth medium of cells. Cells incorporate the 4tU into their newly synthesized RNA. We collect cells after the desired time of incubation with 4tU and extract the total RNA. The newly synthesized RNA is linked to biotin by specifically biotinylating the 4tU ("4tU-labelled" RNA). Simultaneously, the pre-existing RNA that does not contain 4tU is not biotinylated. Finally, the 4tU-labelled RNA is separated from the pre-existing RNA without 4tU through a pull-down with magnetic beads containing streptavidin, after which the purified 4tU-labelled RNA is sequenced (see Methods for experimental details). **(b)** Schematic illustrating the experimental procedure for measuring the genome-wide transcription dynamics at near-freezing temperatures. We incubated liquid cultures with populations of wild-type yeasts at the desired temperature (e.g., 5.0 °C). After two weeks of incubation, we first collected two aliquots of our cultures (as "time 0" hours of the time-lapse). ***(caption continues)***

Supplementary Fig. 34 ***(continued)*****:** Directly after, we supplemented the growth medium of the cultures with 4tU at a final 5 mM concentration, and collected aliquots from our cultures at the desired time-points (see for experimental details). After collecting the time-lapse samples, we added a fixed amount of 4tU-labelled *S. pombe* (*Schizosaccharomyces pombe*) cells to all 4tU time-lapse samples as a spike-in and proceeded with RNA-extraction. Finally, we used the extra ”time 0” hours aliquots to quantify the steady-state transcript levels (grey box, RNA-seq and RNA FISH). **(c)** The *S. pombe* spike-in that was added to all 4tU time-lapse samples enabled us to normalize the amount of 4tU-labelled transcripts from different time-points. Initially, all 4tU-labelled RNA in the sample is from the *S. pombe* spike-in since the cold-incubated *S. cerevisiae* cells have not initially synthesized 4tU-labelled RNA. Over time, the cold-incubated cells synthesize new 4tU-labelled RNA and degrade old (unlabelled) RNA, increasing the percentage of 4tU-labelled RNA that is from the cold-incubated cells. Normalizing with the amount of spike-in transcripts therefore ensures that the relative differences between samples in a time-lapse are conserved (since the amount of 4tU-labelled RNA from *S. pombe* cell was the same in every sample). Moreover, this procedure eliminates experimental differences between samples due processing of RNA and sequencing.

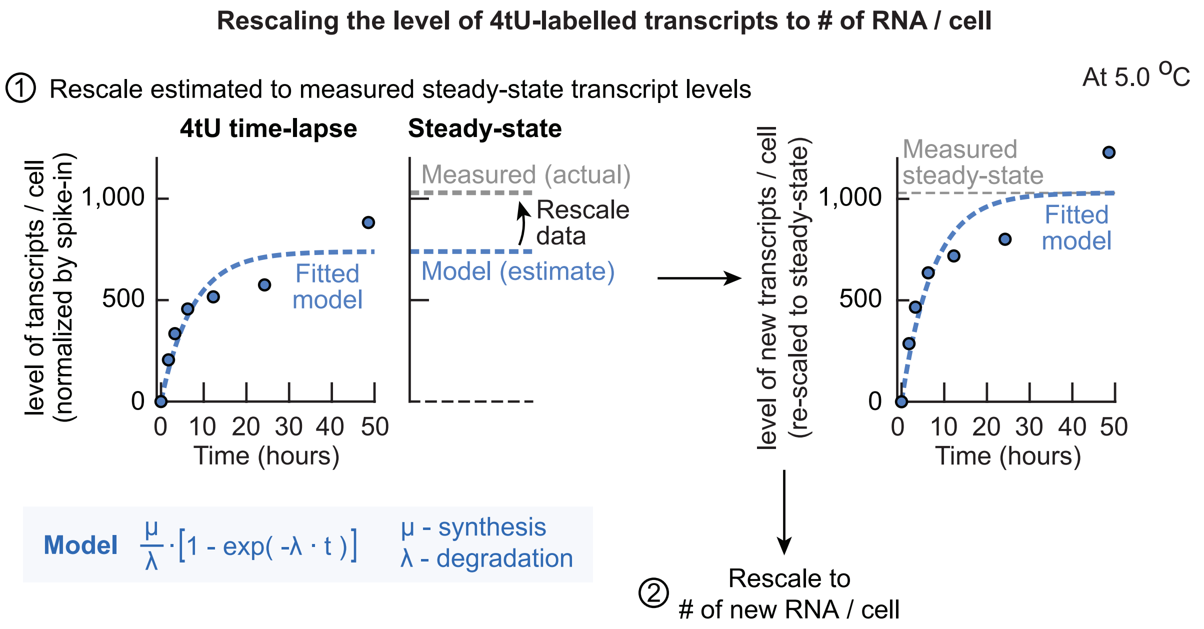

Supplementary Fig. 35: Procedure for rescaling the 4tU transcript levels using the steady-state transcript levels (Related to Figure 5b). First, the 4tU time-lapse samples were normalized by the transcript level of spike-in RNA and the number of alive cells in the population that we had also measured, giving the relative 4tU transcript level per cell over time (the amount of 4tU-labelled RNA normalized by the 4tU spike-in RNA). Next, these relative values are rescaled to obtain the "# of new RNA / cell". To do so, the samples are rescaled such that steady-state transcript level per cell that is estimated by the 4tU time-lapse is equal to the measured steady-state transcript level per cell that we measured through RNA-seq (Supplementary Fig. 34). To obtain the estimated steady-state transcript level per cell, we fitted a mathematical model to the relative 4tU transcript levels per cell over time. The conventional model to describe RNA synthesis assumes that RNA is synthesized at a constant rate and degraded at a rate that is dependent on the concentration [30, 29]. Thus, the kinetics of 4tU-labelled RNA are given by $\frac{dN}{dt} = \frac{\mu}{\lambda}\left(1 - \exp(-\lambda t)\right)$ with a RNA synthesis rate μ (in "# of RNA / cell / hour"), a RNA degradation rate λ (in "per hour") and time t. Over time, the relative 4tU transcript level per cell converges to a steady-state μ/λ (in "RNA / cell") by degradation of old RNA and synthesis of new (4tU-labelled) RNA [30, 29]. To obtain the measured steady-state transcript levels per cell we had also sequenced the steady-state transcript level (total RNA, not 4tU purified RNA) of each time-lapse together with a *S. pombe* spike-in to normalize our time-lapses across temperatures (the steady-state transcript levels can differ per temperature, and the same spike-in RNA was used for all steady-state samples). Thus, we rescaled all 4tU-samples such that the estimated steady-state transcript levels of the model matched the experimentally measured steady-state transcript levels. Shown is a representative example of such a rescaling from a 4tU time-lapse without added extracellular GSH at 5.0 °C. Note that this rescaling conserves the relative differences between all time points within each time-lapse. ***(caption continues)***

Supplementary Fig. 35 ***(continued)*****:** By applying this methodology to all time-lapses from each condition, we obtained the relative level of new transcripts (4tU-labelled RNA) over time between replicates and across temperatures. Moreover, the estimated steady-state of each time-lapse is equal to the experimentally measured steady-state of that time-lapse. As a last step, the relative levels of new transcripts per cell will be rescaled to integer number of RNA per cell using the steady-state samples and RNA FISH (Supplementary Fig. 36). Dots show raw (normalized) data for one replicate. Blue dotted lines shows model fit for that replicate. Grey dotted lines shows measured steady-state values.

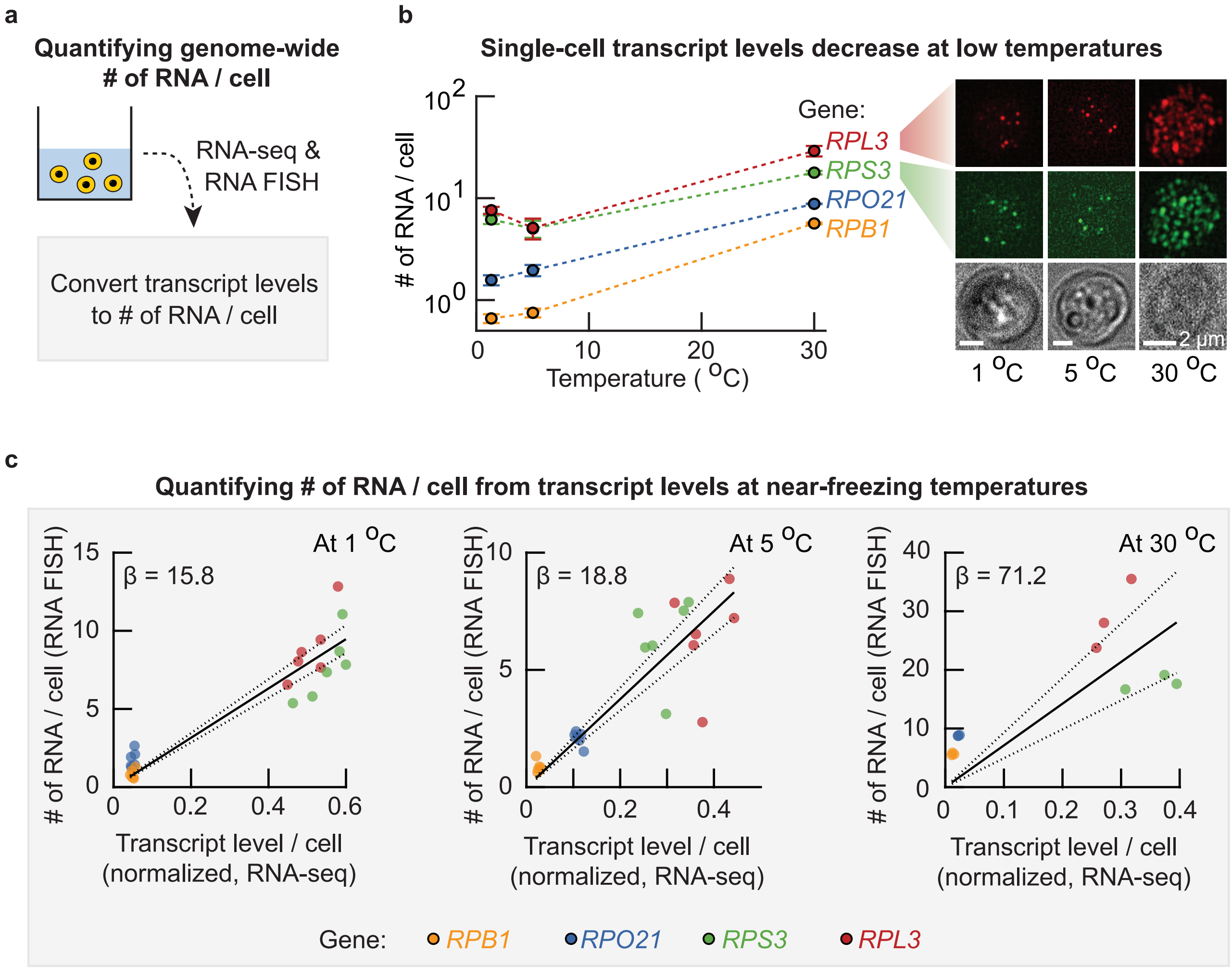

Supplementary Fig. 36: Quantifying the genome-wide number of RNA per cell from the relations between RNA-seq and RNA FISH data in steady-state (Related to Figure 5b). **(a)** Steady-state transcript levels were quantified in two ways. In one way, we performed regular RNA-seq on the steady-state RNA (normalized across temperatures by a spike-in of total RNA from *S. pombe*). In the other way, we quantified the number of RNA per cell for endogenous yeast genes via single-molecule RNA FISH with cells from the same aliquots on which we performed RNA-seq (Methods). These measurements were then combined to re-scale the relative transcript levels per cell (from RNA-seq) to the "# of RNA / cell" as measured by RNA FISH (Supplementary Figs. 34-35). **(b)** The single-cell number of transcripts for several endogenous yeast genes were quantified through RNA FISH at 30 °C, 5.0 °C and 1.0 °C. Shown is the average number of RNA per cell as function of temperature for *RPL3* (red curve), *RPS3* (green curve), *RPO21* (blue curve) and *RPB1* (yellow curve). The average number of mRNA per cell decreases with temperature. ***(caption continues)***

Supplementary Fig. 36 ***(continued)*:** Each data point shows an average of n = 3 biological replicate populations with error bars representing s.e.m. Images show representative examples of labelled RNA in single-cells at 1.0 °C (left), 5.0 °C (middle) and 30 °C (right). Dots are labelled RNA of *RPL3* (red fluorescence, top) or *RPS3* (green fluorescence, bottom). Brightfield images are shown as a comparison. Scale bar is 2 μm. **(c)** The average number of RNA per cell from RNA FISH as a function of the relative steady-state transcript levels per cell from RNA-seq at 1.0 °C (left panel), 5.0 °C (middle panel) and 30 °C (right panel). Shown are the steady-state transcript levels for *RPB1* (yellow dots), *RPO21* (blue dots), *RPS3* (green dots) and *RPL3* (red dots). Black solid line shows a linear fit without intercept, dotted lines show 95% confidence interval of the fit. Since both RNA FISH and RNA-seq measurements were performed on the same cell populations, these linear fits yield scaling factors β that converts the relative transcript levels per cell to the "# of RNA / cell" for each temperature (β = 15.8 at 1.0 °C (Pearson-correlation coefficient ρ = 0.94), β = 18.8 at 5.0 °C (ρ = 0.87) and β = 71.2 at 30 °C (ρ = 0.79)). We used these scaling factors to rescale the relative level of 4tU-labelled transcripts per cell (from 4tU-seq; Supplementary Figs. 34-35) to integer numbers of RNA per cell for all temperatures. All colors show 6 dots, having n = 3 biological replicates each for 250 μM added GSH and without added GSH.

RNA synthesis rates and RNA half-lives for single genes at near-freezing temperatures

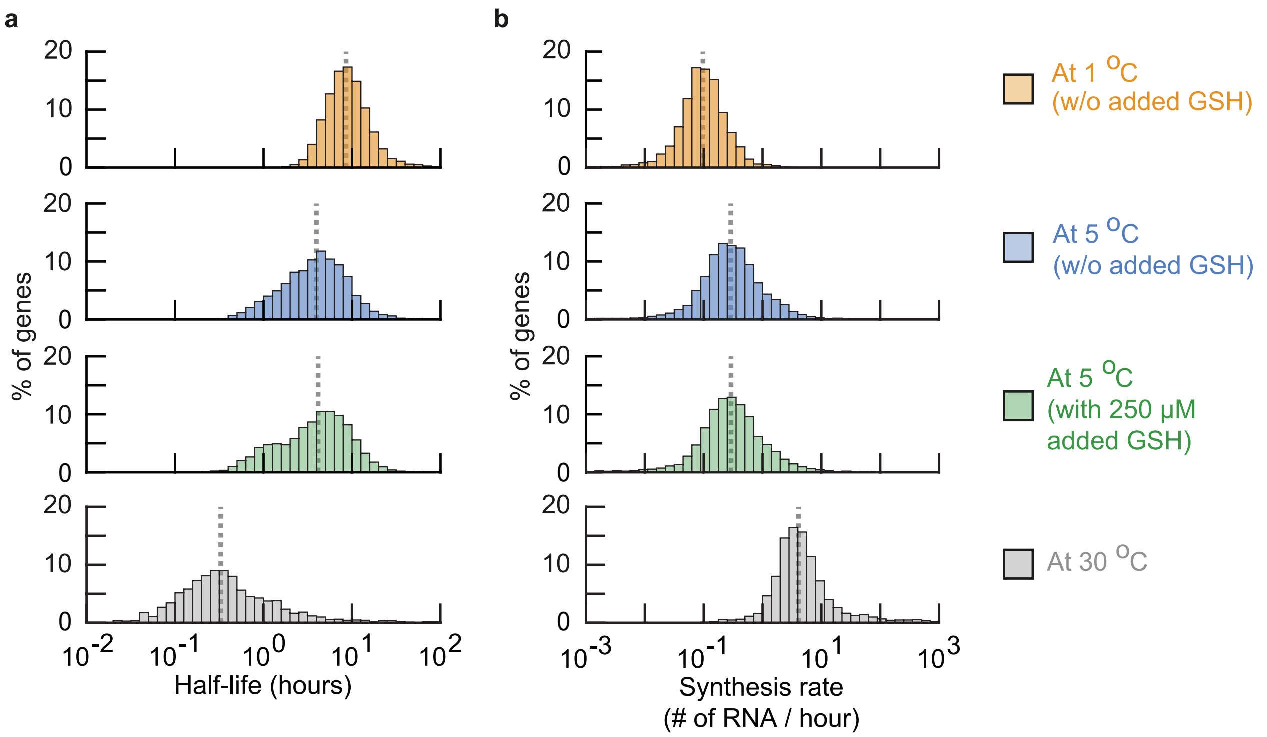

Supplementary Fig. 37: Single-gene RNA synthesis rates and RNA half-lives at near-freezing temperatures (Related to Figure 5b). (a-b) Using the data of freshly synthesized RNA per cell over time for the whole genome (as determined by 4tU-seq; Supplementary Figs. 35-36), we fitted the model for the kinetics of RNA synthesis for every individual yeast gene. Histograms show the mRNA half-lives **(a)** and mRNA synthesis rates **(b)** for 4, 955 genes in the *S. cerevisiae* genome at 1.0 °C without added GSH (yellow bars), at 5.0 °C without added GSH (blue bars) and at 5.0 °C with 250 μM added GSH (green bars). The RNA half-lives and RNA synthesis rates at 30 °C are included as a comparison ($n = 2,135$ genes, grey bars). Grey dotted lines show the median mRNA half-lives (8.57, 3.95 and 4.13 hours) and the median mRNA synthesis rates (0.10, 0.29 and 0.29 RNA / cell / hour) for each condition (at 1.0 °C, and at 5.0 °C without added GSH and with added glutathione respectively). The median RNA half-life at 30 °C is 14.3 minutes and the median RNA synthesis rate is 4.1 RNA / cell / hour. The distribution of RNA synthesis rates and RNA half-lives remains almost unchanged upon addition of extracellular GSH, suggesting that ROS does not change the transcriptional dynamics at near-freezing temperatures.

Transcriptional dynamics depend on the class of genes

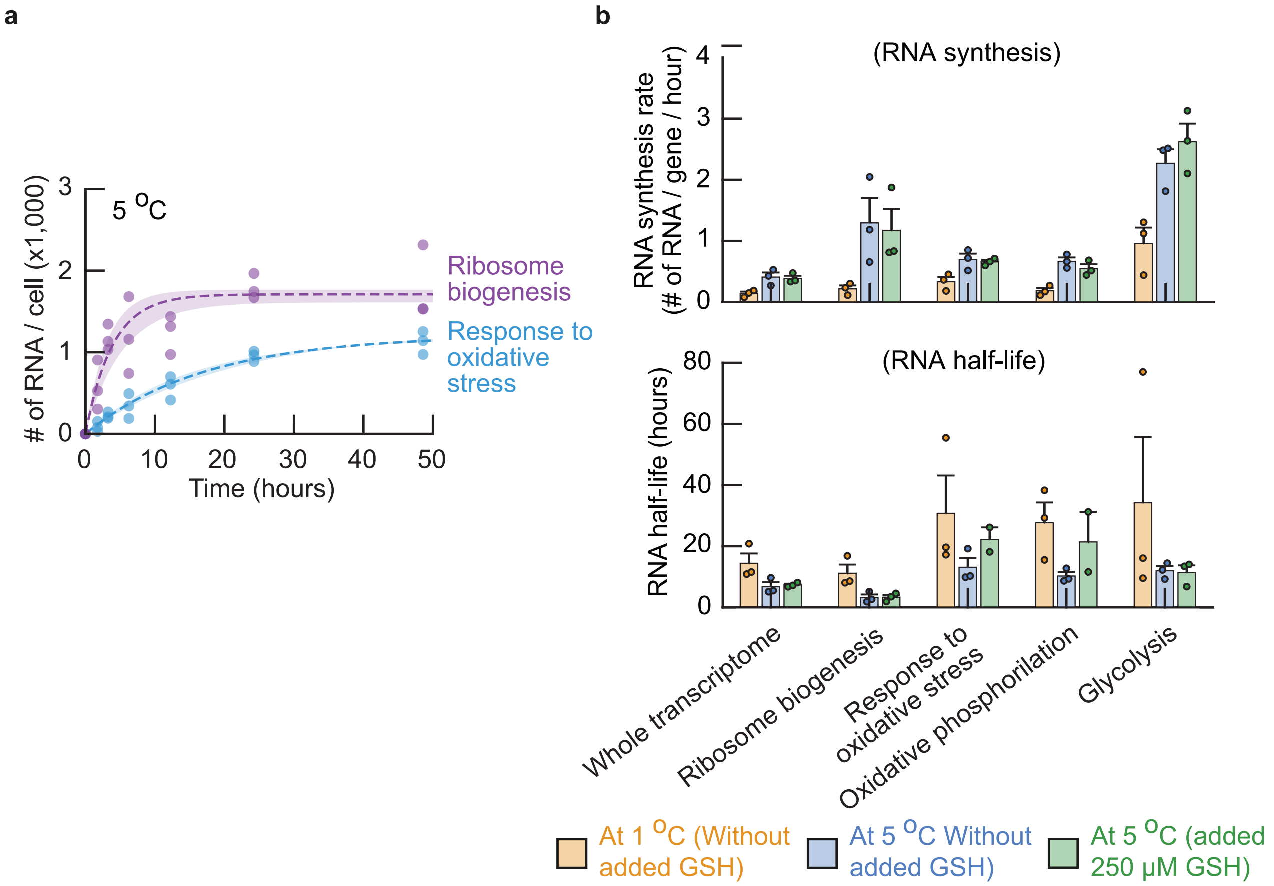

Supplementary Fig. 38: Transcriptional dynamics differ between gene groups at freezing temperatures (Related to Figure 5b. (a-b) Using the data of freshly synthesized RNA per cell over time (as determined by 4tU-seq; Supplementary Figs. 35-36), we also fitted the model for the kinetics of RNA synthesis to the amount of 4tU-labelled RNA in groups of related genes (as defined by Gene Ontology). **(a)** Number of mRNA per cell over time at 5.0 °C for genes belonging to Ribosome biogenesis (GO:0042254, 341 genes) or response to oxidative stress (GO:0006979, 101 genes). Genes associated to ribosome biogenesis have fast transcriptional dynamics compared to genes associated to the response to oxidative stress. Dots show measured data with the lines showing model fitted to the mean from n = 3 biological replicate populations for each condition. Shaded bands represent s.e.m. **(b)** Average mRNA synthesis rate (number of mRNA per gene per hour) and average mRNA half-life for all genes within each group of genes at various temperatures. Shown are each group at 1.0 °C without added GSH (yellow bars), at 5.0 °C without added GSH (blue bars) and at 5.0 °C with 250 μM added GSH (green bars). The included groups are ribosome biogenesis (GO:0042254; 341 genes), response to oxidative stress (GO:0006979; 101 genes), oxidative phosphorylation (GO:0006119; 27 genes) and glycolysis (GO:0006096; 19 genes). ***(caption continues)***

Supplementary Fig. 38 ***(continued)*****:** The whole transcriptome (4955 genes) is included as a comparison. Bars represent mean with error bars showing s.e.m.; n = 3 biological replicate populations for each condition. These rates show that the transcriptional dynamics vary widely between genes that are associated with different processes. Strikingly, at 5 °C, the gene groups associated with oxidative processes have both a slower mRNA synthesis rate and a longer mRNA half-life – more stable RNA – when ROS is less abundant (with added GSH). For example, the RNA of genes associated to the response to oxidative stress have a half-life of 13.1 ± 3 hours without added GSH compared to 22.2 ± 4.0 hours with added GSH. Thus, the turn-over of mRNA associated to oxidative processes seems slower in cells with less abundant ROS at near-freezing temperatures.

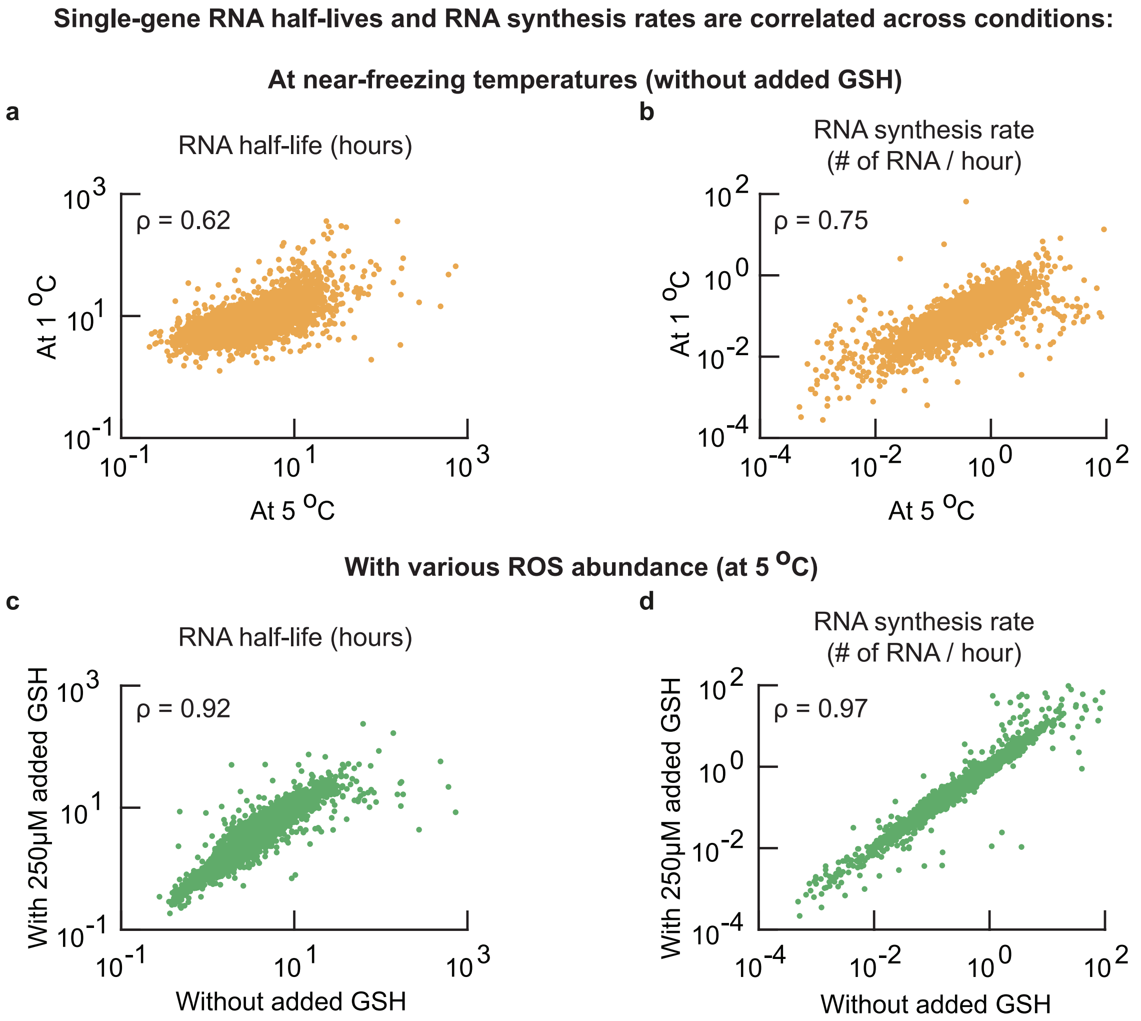

Supplementary Fig. 39: Single-gene mRNA half-lives and mRNA synthesis rates are correlated across temperatures and ROS abundance (Related to Figure 5b). Comparing single-gene transcription dynamics across near-freezing temperatures and with various ROS abundances using the single-gene transcriptional rates determined in Supplementary Fig. 37). **(a-b)** Single-gene transcription rates compared between 5.0 °C (x-axis) and 1.0 °C (y-axis) without added GSH. Shown are the single-gene mRNA half-lives (Pearson-correlation coefficient ρ = 0.62) **(a)** and the RNA synthesis rates (Pearson-correlation coefficient ρ = 0.75) **(b)**. **(c-d)** Single-gene transcriptional rates at 5.0 °C compared without added GSH (x-axis) and with 250 μM added GSH (y-axis). Shown are the single-gene mRNA half-lives (Pearson-correlation coefficient ρ = 0.92) **(c)** and RNA synthesis rates (Pearson-correlation coefficient ρ = 0.97) **(d)**. The half-lives and synthesis rates of RNA remain almost unchanged upon addition of GSH to the growth media, supporting the fact that ROS does not change the transcriptional dynamics at near-freezing temperatures. Each dot in all panels shows the rate for a single gene averaged over n = 3 biological replicates.

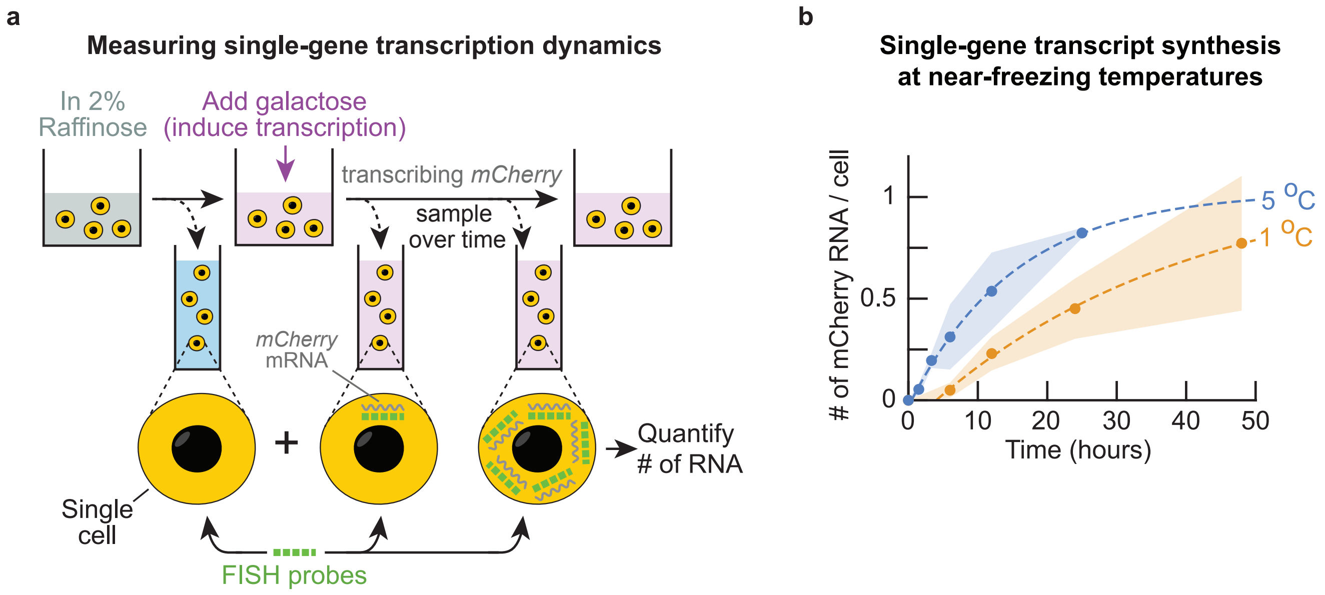

Supplementary Fig. 40: Single-gene transcription dynamics at near-freezing temperatures (Related to Figure 5b). We used a mCherry-inducible strain to measure the transcription dynamics for a single gene at near-freezing temperatures. **(a)** Schematic showing an experiment to measure single-gene transcription (Methods). Liquid cultures with populations of the mCherry-inducible strain were incubated at the desired temperature with 2% raffinose as the carbon source. After two weeks of incubation, we took aliquots of these liquid cultures (as a control without mCherry). Directly after, we supplemented the growth media with 2% galactose to induce the expression of mCherry, and continued to collect aliquots of these cultures at the desired time points. We then performed RNA FISH on the mCherry RNA for each aliquot, and quantified the average number of RNA per cell over time. **(b)** Single-gene transcription dynamics at near-freezing temperatures. Shown is the number of mCherry RNA per cell as function of time at 5.0 °C (blue curve) and at 1.0 °C (orange curve). Dotted line shows a model for the kinetics of RNA synthesis fitted to the average number of RNA per cell (Supplementary Fig. 35). The fitted model yields a RNA synthesis rate of 0.03 RNA / cell / hour and a RNA half-life of 26.0 hours at 1.0 °C, and a RNA synthesis rate of 0.07 RNA / cell / hour and a RNA half-life of 10.6 hours at 5.0 °C. These measurements show that the time-scale of transcription for mCherry (reaching half the steady-state in ∼10 hours) is considerably slower than the time-scale of protein synthesis at near-freezing temperatures (no appreciable increase in fluorescence within the first day of mCherry induction, Fig. 5c). Dots show the average of the mean number of RNA per cell in each replicate, shaded area represents the s.e.m., having $n = 3$ (at 1 °C) biological replicate populations.

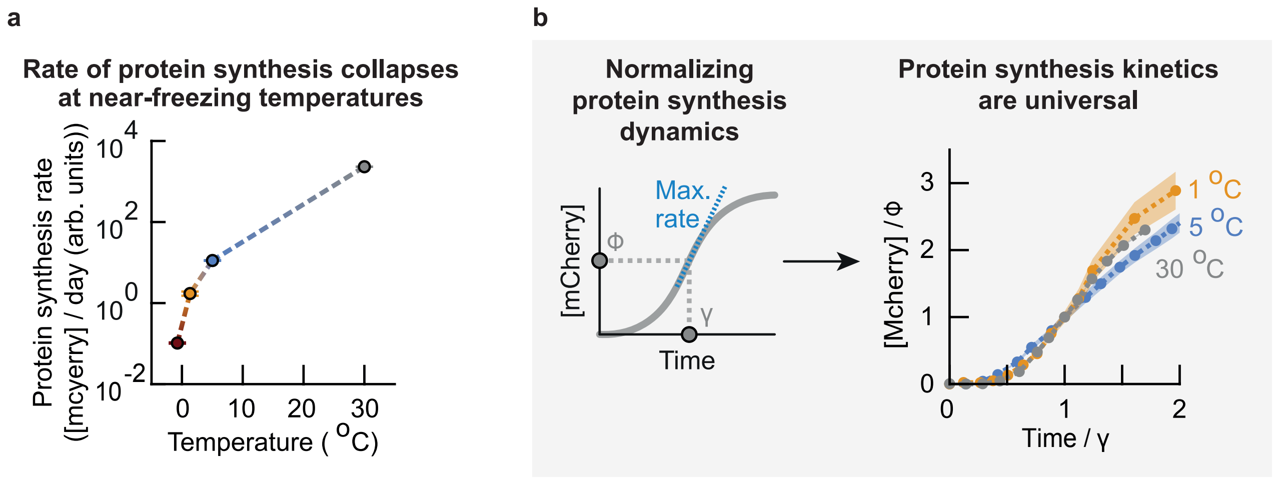

Supplementary Fig. 41: The protein-synthesis rate collapses as water freezes (0 °C) (Related to Figure 5c). Using a mCherry-inducible strain to the measure protein-synthesis rate across temperatures (Supplementary Fig. 44). **(a)** For each curve in Fig. 5c we determined the maximum rate of mCherry synthesis (maximum slope *m* of each fluorescence curve; Supplementary Theory). Shown are the protein-synthesis rates as function of temperature (the synthesis rate of general proteins can be at least as fast as the synthesis rate of mCherry measured here). **(b)** Protein-synthesis dynamics across temperatures. *Left*: Schematics of relevant parameters. At each temperature, we used a characteristic time γ and characteristic fluorescence ϕ at which the population reaches its maximum rate of protein synthesis to rescale protein-synthesis dynamics. *Right*: Normalized mCherry fluorescence over time (Fig. 5c). The fluorescence was normalized with the characteristic fluorescence ϕ and time was rescaled with the characteristic time γ at each temperature. The normalized curves approximately collapse onto a single master curve. These normalized curves suggest that one only needs to know the characteristic fluorescence-scale ϕ and time-scale γ for each temperature to describe expression of mCherry at that temperature. In both panels the error bars and shaded areas show the mean with s.e.m., having n = 3 replicate populations.

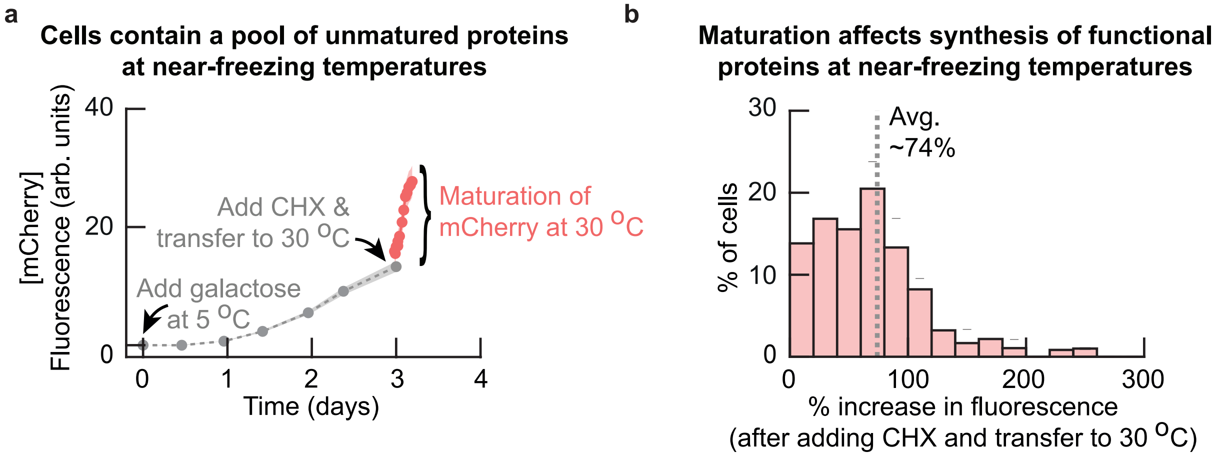

Supplementary Fig. 42: Time to synthesize a functional protein is affected by maturation (e.g., folding) time at 5 °C (Related to Figure 5c). Using a mCherry-inducible strain to quantify the time to synthesize functional proteins at near-freezing temperatures (also see Supplementary Figs. 44 and 41). **(a)** We incubated cells of the inducible mCherry strain for two weeks at 5.0 °C. We then added galactose to the growth media and followed the expression of mcherry at 5 °C in single cells over time with a microscope (Methods). After three days of incubation with galactose, we added cycloheximide (CHX) to the growth medium at a final 100 μg / mL concentration to instantly stop the translation machinery in the cells. We then transferred the cells to 30 °C and continued to measure the mCherry fluorescence of cells to quantify the maturation of unmatured mCherry. Shown is the fluorescence of mCherry over time at 5 °C (grey curve), and the increase in fluorescence at 30 °C after addition of CHX (red curve). The average fluorescence considerably increases after addition of CHX. Cells therefore contain a pool of unmatured mCherry at 5 °C when synthesis is induced with galactose. Dots show average fluorescence, and shaded area shows s.e.m. of the average fluorescence in n = 5 populations. **(b)** Histogram shows the increase in fluorescence of single cells from (a) after addition of CHX and transfer to 30 °C. The average fluorescence increases by 74% after addition of CHX, showing that roughly half of mCherry was not yet mature (fluorescent) at the time of adding CHX. Synthesis of functional proteins is therefore affected by the time required for maturation at near-freezing temperatures (see Supplementary Notes). Histogram contains n = 108 cells. Each histogram is an average histogram and is representative of n = 5 biological replicate populations.

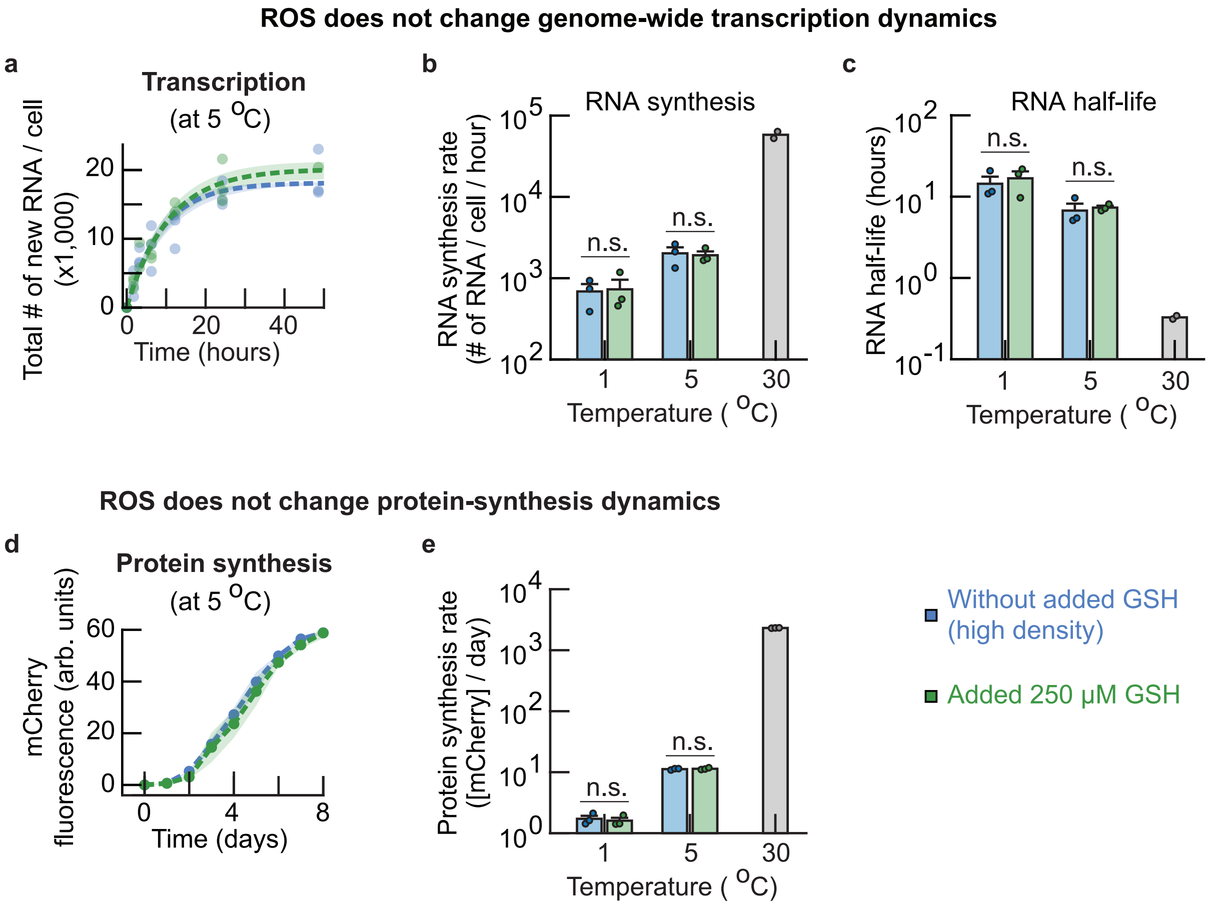

Supplementary Fig. 43: ROS does not change genome-wide transcription dynamics or protein synthesis dynamics at near-freezing temperatures (Related to Figure 5d). (a-c) ROS does not change rates of genome-wide transcription. **(a)** We used the measured number of synthesized RNA over time to fit a model for the kinetics of RNA synthesis in cells (Supplementary Figs. 35-36). Shown are the number of newly synthesized RNA per cell over time at 5.0 °C for populations without added GSH (blue curve) or with 250 μM added GSH (green curve). Dots show measured data. Dotted lines represent fitted kinetics model using average parameter estimates, with n = 3 biological replicates per condition. Shaded area represents the s.e.m. for fitted model parameters. From the fitted model we obtain the RNA synthesis rates **(b)** and RNA half-lives **(c)** at 1.0 °C and 5.0 °C. Included are the rates for populations without added GSH (blue bars) and with 250 μM added GSH (green bars). Rates at 30 °C are shown as a comparison (grey bars). The RNA half-lives and RNA synthesis rates decrease with temperature. Without added GSH, the steady-state transcript levels (in # of RNA / cell) are $12,900 \pm 900$ (1.0 °C), $18,300 \pm 800$ (5.0 °C) and 27,300 (30 °C), the RNA synthesis-rates (in # of RNA per cell per hour) are 690 ± 160 (1.0 °C), $2,020 \pm 370$ (5.0 °C) and 58,100 (30 °C), and the RNA half-lives (in hours) are 14.4 ± 3.9 (1.0 °C), 6.7 ± 1.8 (5.0 °C) and 20 (30 °C, in minutes). ***(caption continues)***

Supplementary Fig. 43 *(continued)*: There is no reason to assume that the RNA synthesis rates are different upon addition of GSH (p-values are as follows: p = 0.82 at 5.0 °C and p = 0.89 at 1.0 °C; 'n.s.' means 'not significant' in the bar plots). We determined these p-values with the two-sides Student's t-test with unequal variances and at a 95%-confidence level. Multiple testing/comparison was not applicable here. These are based on n = 3 biological replicate populations per condition (at 1.0°C and 5.0 °C); bars represent the means with error bars representing s.e.m. For 30 °C, bar represents average and data for each biological replicate is shown (n = 2 biological replicates for each condition at this temperature). **(d-e)** ROS does not change rate of protein synthesis. **(d)** The synthesis of mCherry over time at 5.0 °C compared for populations without added GSH (blue curve) and with 250 μM added GSH (green curve) (also see Fig. 5c and Supplementary Fig. 41). The dots show the average fluorescence, with shaded area representing the s.e.m. of n = 3 biological replicate populations for each condition. **(e)** Protein synthesis rates at 5.0 °C and 1.0 °C for populations that were incubated without added GSH (blue bars) or with 250 μM added GSH (green bars). Grey bar shows synthesis rate of mCherry at 30 °C as a comparison. The protein synthesis rate decreases with temperature. Without added GSH, the protein synthesis rates (in mCherry fluorescence per day (fluorescence in arb. units)) are 1.7 ± 0.2 (1.0 °C), 11.2 ± 0.2 (5.0 °C) and 2314 ± 10 (30 °C). There is no reason to assume that the protein synthesis rates are different upon addition of GSH (p-values are: p = 0.66 at 5.0 °C and p = 0.69 at 1.0 °C; 'n.s.' means 'not significant' in the bar plots). We determined these p-values with the two-sides Student's t-test with unequal variances and at a 95%-confidence level. Multiple testing/comparison was not applicable here. These are based on n = 3 biological replicate populations per condition (at 1.0°C and 5.0 °C); bars represent the means with error bars representing s.e.m.

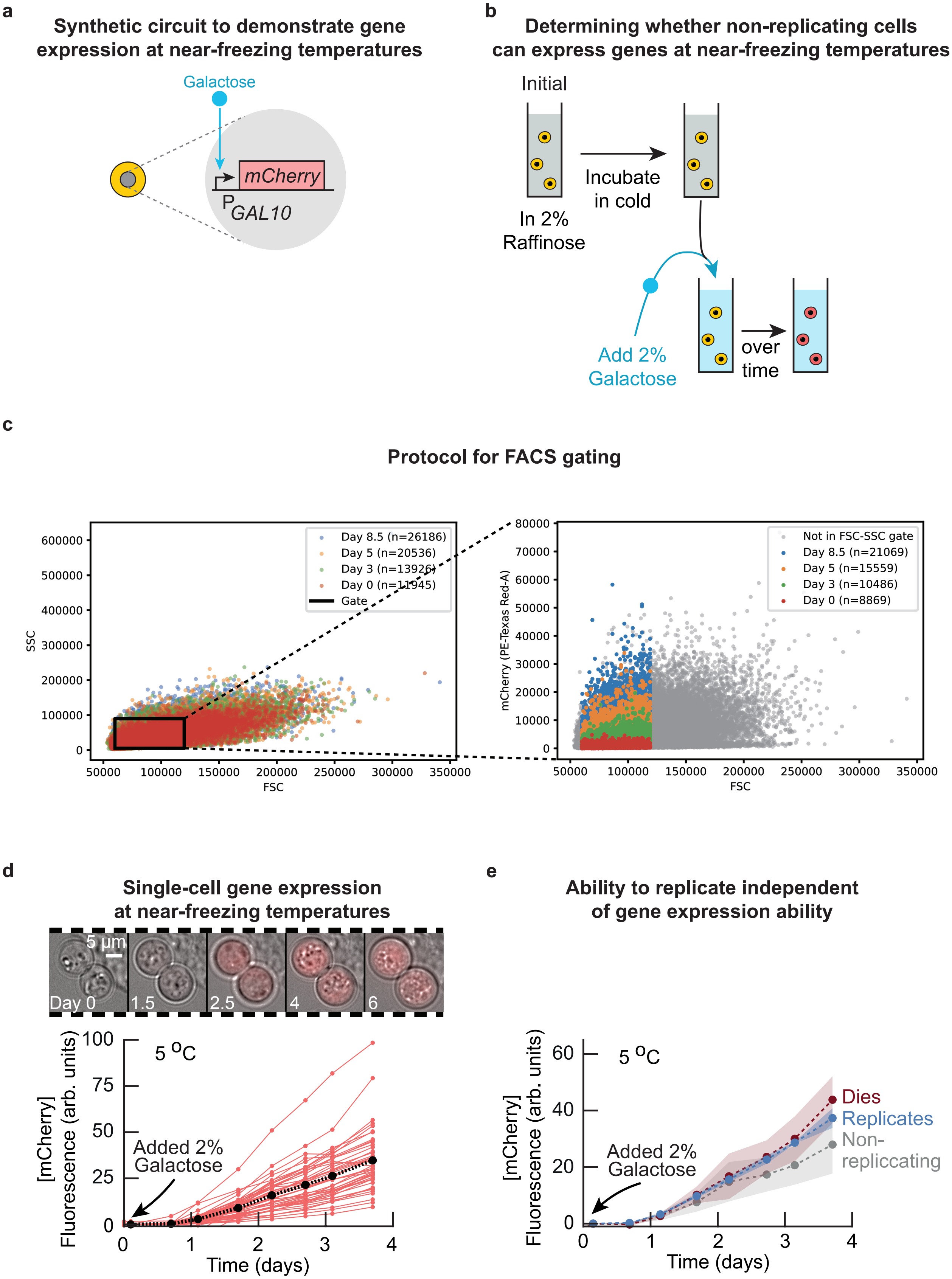

a
Synthetic circuit to demonstrate gene expression at near-freezing temperatures
Galactose
mCherry
PGAL10
b
Determining whether non-replicating cells can express genes at near-freezing temperatures
Initial
In 2% Raffinose
Incubate in cold
Add 2% Galactose
over time
c
Protocol for FACS gating
Day 8.5 (n=26186)
Day 5 (n=20536)
Day 3 (n=13926)
Day 0 (n=11945)
Gate
SSC
FSC
Not in FSC-SSC gate
Day 8.5 (n=21069)
Day 5 (n=15559)
Day 3 (n=10486)
Day 0 (n=8869)
mCherry (PE-Texas Red-A)
FSC
d
Single-cell gene expression at near-freezing temperatures
5 µm
Day 0
1.5
2.5
4
6
5 °C
Added 2% Galactose
[mCherry] Fluorescence (arb. units)
Time (days)
e
Ability to replicate independent of gene expression ability
5 °C
Added 2% Galactose
Dies
Replicates
Non-repliccating
[mCherry] Fluorescence (arb. units)
Time (days)

Supplementary Fig. 44: Non-replicating cells can express genes and the general machineries of gene expression (RNA polymerases and ribosomes) properly function at 5 °C (Related Figure 5d). (a) We build a synthetic gene-circuit in our wild-type yeast that makes the cells produce a red fluorescent protein, mCherry, upon induction by galactose. Expression of mCherry is controlled by an inducible promoter, *pGAL10*, which is activated by galactose. We first grow the yeast in raffinose and then add galactose to the raffinose-medium to activate the expression of mCherry. **(b)** Schematics of an example experiment to test if the engineered cells can express genes (such as mCherry) at near-freezing temperatures. We prepared cultures of the engineered strain in minimal medium that contained 2% raffinose as the carbon source (Methods). These populations were then incubated at 5 °C for two weeks, after which we induced the expression of mCherry by adding 2% galactose to the growth medium and then incubating the cells at 5 °C for the duration of the time-lapse. The expression of mCherry was then measured through either microscopy or by flowing aliquots of the populations through a flow cytometer (Methods). **(c)** Protocol for gating cells in our FACS analyzer for measuring their mCherry protein levels. We selected yeast cells that were inside the black rectangle shown in the FSC-SSC plot (left graph). Only their mCherry levels were measured and recorded (shown as colored dots in the FSC-mCherry plot (right graph)). **(d)** Time-lapse microscopy movie shows mCherry fluorescence measured in a single-cell over time. Each snapshot shown in the filmstrip is a composite of brightfield and mCherry channels. Shown are the cells on day 0 (i.e., directly after adding galactose) and after 1.5, 2.5, 4 and 6 days. Scale bar is 5 μm. Graph shows mCherry fluorescence in single cells over time from a microscopy time-lapse of a population kept at 5 °C (day 0 is the time of addition of 2% galactose). Black dotted line shows population average (n = 44 cells). All cells express mCherry within four days of induction with galactose. **(e)** From our microscopy time-lapses we also determined the average mCherry fluorescence over time for cells that duplicated (blue), cells that died (red) and cells that grew without duplicating (grey). Non-replicating cells and dying cells also express mCherry, at levels that are similar to that of replicating cells. Error bars show the mean with s.e.m. of the average fluorescence of each biological replicate (n = 3). With mCherry as a model gene, this experiment shows that the general machineries of transcription and translation (e.g., RNA polymerases and ribosomes) properly function to yield gene expression at 5.0 °C. Thus, the replicative abilities of cells is not dependent on the ability of cells to express genes at near-freezing temperatures.

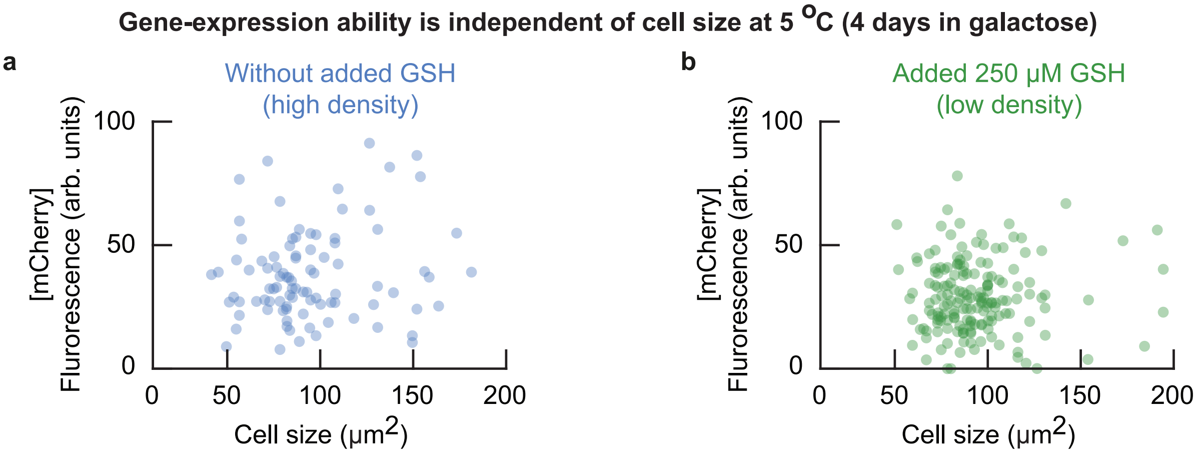

Supplementary Fig. 45: Gene-expressing ability is independent of cell size (Related to Figure 5d). Populations of the mCherry-inducible strain were incubated at 5.0 °C for two weeks with raffinose as the carbon source (i.e., without galactose). After two weeks, we added 2% galactose to their growth medium and started making microscopy time-lapse movies that followed the expression of mCherry in single cells at 5.0 °C. Shown is the expression of mCherry as function of cell size 4 days after adding galactose for high-density populations ($\sim$8,000 cells / mL) **(a)**, and for low-density populations ($\sim$420 cells / mL) that were incubated with 250 μM GSH **(b)**. The mCherry protein level in a cell is uncorrelated with the cell's size for both populations. This result supports the conclusion that gene-expressing ability does not depend on cell size at 5.0 °C.

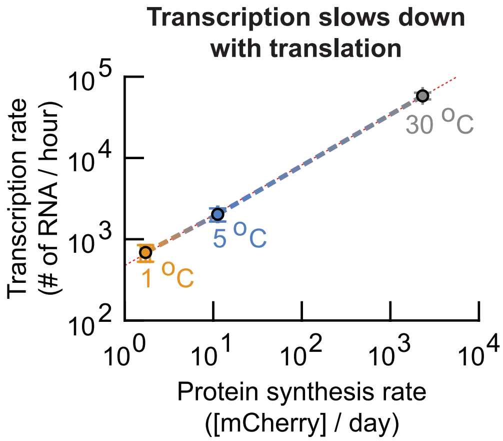

Supplementary Fig. 46: Transcription slows down with the protein synthesis rate (Related to Figure 5d). Using the transcription rate (# of synthesized RNA / cell / hour) and protein synthesis rate (mCherry fluorescence / day, (arb. units)) across temperatures for the populations incubated without added GSH (Supplementary Fig. 43). Log-log plot shows how the transcription rate scales with the protein synthesis rate across temperatures (also see Fig. 5d). The red dotted line shows a power-law fit (exponent 0.62 ± 0.01, Pearson correlation-coefficient $\beta = 0.9998$). This power-law scaling confirmed that protein synthesis slows down faster than transcription as temperature decreases (exponent below 1; Supplementary Theory), such that transcription becomes more limiting for cell replications than protein synthesis as temperature continues to decrease (i.e., the fold-change in the protein synthesis rate is always larger than the fold-change in the transcription rate as temperature decreases, so that protein synthesis becomes more limiting than transcription upon a decrease in temperature). Dots show experimentally measured average values (Supplementary Fig. 43), error bars show s.e.m. of $n = 3$ biological replicates.

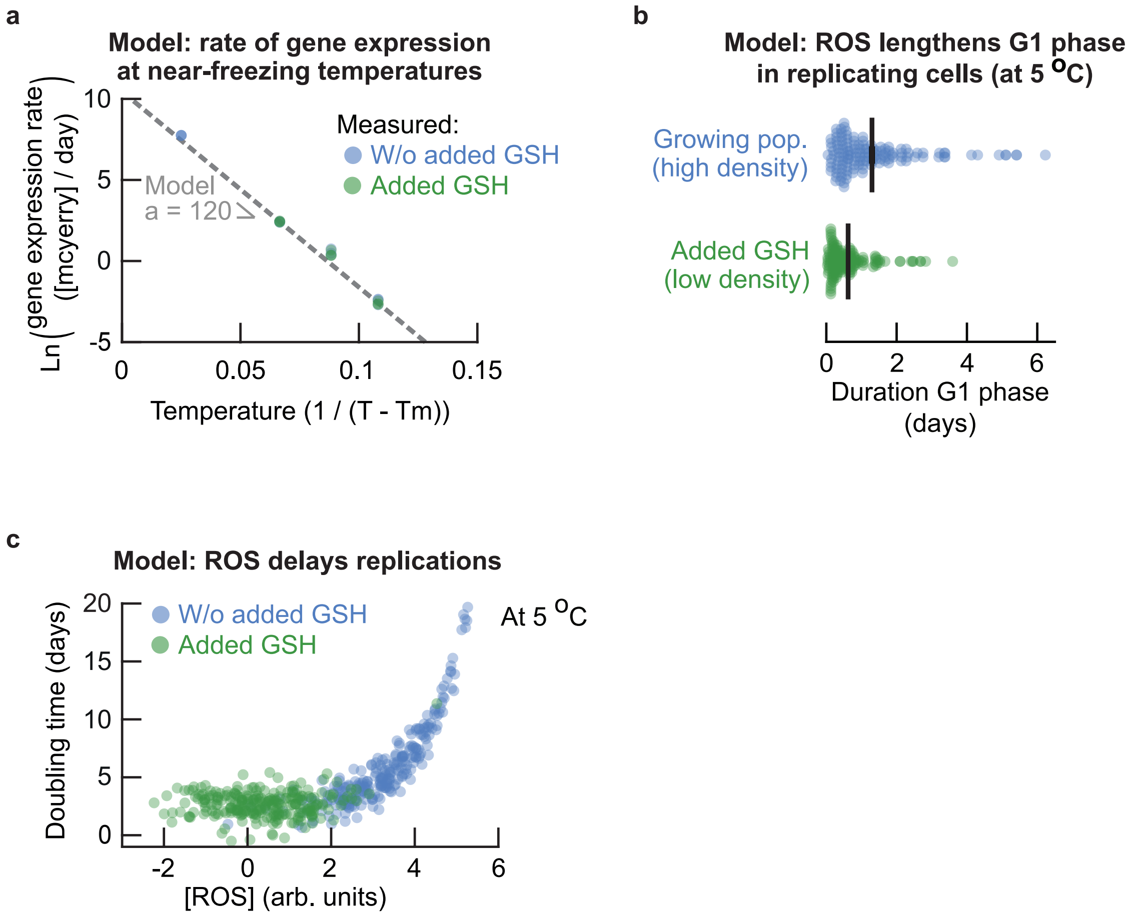

Supplementary Fig. 47: Stochastic model for the duration of single-cell replication at near-freezing temperatures (Related to Figure 6). (a) Estimating the parameter a for the model for single-cell doubling times at near-freezing temperatures (Supplementary Theory). To do so, we fitted a linear model to the protein synthesis rate $r_g(T)$ as function of temperature T using an Arrhenius-type equation ($\ln r_g(T) \propto -a/(T - T_m)$, see equation S13 and Supplementary Theory for derivation). This model assumes a minimum temperature $T_m = -10$ °C below which protein synthesis does not occur. Fitting this linear model to the experimental data (Supplementary Fig. 43) yields $a \approx 120.72$. **(b-c)** Using the model to simulate the doubling time of individual cells and G1 phase durations (Supplementary Theory). **(b)** Simulation of the ROS-dependent G1 duration of the cell cycle at 5.0 °C (equal to the additional time Δt in the model, compare with Fig. 4c). The only free parameter (i.e., the average intracellular ROS concentration R of cells in the population) was chosen such that the average additional time Δt equals the average measured G1 duration for each condition respectively (Fig. 4c, the parameter R in equation S20 of Supplementary Theory). This yielded $R = 2.4$ for high-density (growing) populations (blue dots) and $R = 1.55$ for low-density populations with added GSH (green dots). Error bars show the mean with s.e.m. of $n = 3$ replicate simulations that each have $n = 50$ cells. Dots show the simulated additional time Δt for individual cells. ***(caption continues)***

Supplementary Fig. 47 ***(continued)*****: (c)** Simulation of the ROS-dependent doubling times of individual cells at 5.0 °C without added GSH (blue dots) and with added GSH (green dots). Initially, for relatively low ROS concentrations, the ROS-dependent additional time Δt is negligible compared to the shortest doubling time t that is set by the protein synthesis rate. Thus, at low ROS concentrations (green dots), the doubling time τ is approximated by the shortest doubling time t with noise (Supplementary Theory, equation S18). For relatively high ROS concentrations, the ROS-dependent additional time Δt becomes of the same size as the shortest doubling time t, such that the doubling time τ becomes dictated by ROS abundance. Each condition shows a simulation of n = 250 cells. The free parameter of the model was chosen to be equal to the values used to simulate the single-cell doubling times (see Fig. 6c; R = 3.2 without added GSH, R = 0.35 with added GSH).

Supplementary Notes

Density effect emerges from the percentage of replicating and dying cells (related to Fig. 1). Here we establish that the density-dependent growth at 5.0 °C does not emerge from the density-independent single-cell doubling times at 5.0 °C but instead from the percentage of cells in the population that dies and the percentage of cells that replicates (Supplementary Fig. 12). This is illustrated through only considering the initial population of cells in the time-lapse. For example, in high-density (growing) populations, 29% of the population at the start of the time-lapse replicates with a doubling time of ∼6.5 days. These 29% of cells can therefore replicate at least 3x during the ∼20 day time-lapse, thereby expanding the initial population by 88% with newborn cells. During the same ∼20 days, 36% of the population at the start of the time-lapse dies. Thus, the cells within the initial high-density population that die are more than replenished by the newborn cells during the time-lapse. This leads to the high-density populations growing over time. In contrast, in low-density (non-growing) populations, 17% of the population at the start of the time-lapse replicates with a doubling time of ∼7.1 days. The replicating cells in non-growing populations can therefore replicate less than 3x during the ∼20 day time-lapse. The replicating cells then expand the initial population with at most 52% newborn cells, while during the same time 58% of the initial population dies. Then, the number of cells within the initial low-density population that die exceeds the number of newborn cells from this population during the time-lapse. As a consequence, more cells die than there are newborns per unit time, leading to the low-density population to go extinct. In summary, the density effect at 5.0 °C emerges from the percentage of cells that die and the percentage of cells that replicate that depend on the initial population density.

Growth model for expected lifespan and doubling time (related to Fig. 1). Here we derive a simple growth model that describes the expected lifespan and doubling time of cells at near-freezing temperatures. Assume that the number of cells in the population is given by $N(t)$ at time t. Of these $N(t)$ cells, $A(t)$ are alive and $D(t)$ are dead, such that $N(t) = A(t) + D(t)$. The number of alive cells changes over time through cell replications and cell deaths. The rate at which cells replicate is given by μ and the rate at which cells die is given by λ. Then, the number of alive cells changes over time according to,

$$\frac{dA}{dt} = A(t) \cdot (\mu - \lambda). \quad \text{(S1)}$$

Similarly, the total number of cells changes according to,

$$\frac{dN}{dt} = \mu A(t), \quad \text{(S2)}$$

since we add freshly born cells to the total number of cells and dead cells are not removed from the total number of cells in the population. Solving the differential equation S1 yields,

$$A(t + dt) = A(t) \cdot \exp\Big((\mu - \lambda) \cdot dt\Big). \quad \text{(S3)}$$

Using equation S3 to solve equation S2 we obtain,

$$
\begin{aligned}
N(t+dt) &= N(t) + \int_0^{dt} \mu A(\tau) d\tau \\
&= N(t) + \mu A(t) \int_0^{dt} \exp\Big((\mu-\lambda)\tau\Big) \\
&= N(t) + \frac{\mu}{\mu-\lambda} A(t) \Big[\exp\Big((\mu-\lambda)\tau\Big)\Big]_0^{dt} \\
&= N(t) + \frac{\mu}{\mu-\lambda}\Big(A(t+dt) - A(t)\Big) \qquad \text{(S4)}
\end{aligned}
$$

In our experiments we can measure the number of alive cells and the number of dead cells and the total number of cells over time. We therefore have $A(t)$ and $N(t)$ available to fit the growth rate μ and death rate λ with our model. Taking the logarithm of equation S3 describing the number of alive cells in the population and rewriting yields,

$$r_1 := \frac{1}{dt}\Big(\ln A(t+dt) - \ln A(t)\Big) = \mu - \lambda \qquad \text{(S5)}$$

Similarly, we can rewrite equation S4 describing the total number of cells in the population as,

$$r_2 := \frac{N(t+dt) - N(t)}{A(t+dt) - A(t)} = \frac{\mu}{\mu-\lambda}. \qquad \text{(S6)}$$

Finally, we use the numbers r_1 (from equation S5) and r_2 (from equation S6) to find the rates μ and λ,

$$
\begin{aligned}
\mu &= r_1 \cdot r_2, \qquad \text{(S7)} \\
\lambda &= r_1 \cdot (r_2 - 1).
\end{aligned}
$$

We used equations S5-S7 to estimate the growth rate μ and death rate λ from the total number of cells and the number of alive cells in a population. The expected lifespan of cells in the population is then given by $1/\lambda$ (cells die at a rate λ).

Model for protein synthesis rate (Related to Fig. 5). Here we describe a simple kinetics model for the synthesis of mCherry that we measured experimentally by following the mCherry fluorescence in cells over time. Assume that mCherry is synthesized by first being translated into an unfolded polypeptide chain, followed by folding into a fluorescent protein (Supplementary Fig. 42). We let u denote the amount of unfolded mCherry and f denote the amount of folded mCherry in a cell. We further assume that unfolded mCherry is synthesized at a constant rate m (arb. units per day), and that the unfolded mCherry is folded at a rate γ (day^{-1}). Then the amounts of unfolded and folded mCherry over time t are described by,

$$\frac{du}{dt} = m - \gamma \cdot u, \qquad \text{(S8)}$$

$$\frac{df}{dt} = \gamma \cdot u. \qquad \text{(S9)}$$

Solving equation S8 yields $u(t) = \frac{m}{\gamma} \cdot \Big(1 - \exp(-\gamma \cdot t)\Big)$, and substitution of $u(t)$ into equation S9 yields,

$$f(t) = \frac{m}{\gamma} \cdot \Big(\exp(-\gamma \cdot t) - 1\Big) + m \cdot t. \tag{S10}$$

Equation S10 describes the amount of folded mCherry over time and provides a model for the experimentally measured mCherry fluorescence in cells. Initially, synthesis of mCherry has not occurred, such that a cell does not have unfolded mCherry and no folded mCherry is being produced. At time $t = 0$ the cell starts synthesizing unfolded mCherry, whose concentration is described by $u(t) = \frac{m}{\gamma} \cdot (1 - \exp(-\gamma \cdot t))$. Thus, the amount of unfolded mCherry initially increases and eventually reaches a steady-state that is determined by an inflow from synthesis and an outflow to folded mCherry ($u(t) \rightarrow \frac{m}{\gamma}$ as $t \rightarrow \infty$). At this steady-state, the equation S8 for unfolded mCherry satisfies $\frac{du}{dt} = 0$, such that $\gamma \cdot u = m$ and $\frac{df}{dt} = m$ (from equation S9). Thus, this derivation shows that the slope of mCherry fluorescence over time converges to the synthesis rate m ($\frac{df}{dt} \approx m$ for sufficiently large t). In practice, we measured the synthesis rate $m(T)$ for each temperature T by taking the maximum slope of the increase of mCherry fluorescence over time, and then used these $m(T)$ to further fit the model in equations S8-S9 to the data.

0.1 Stochastic model for speed limits of cellular life in frigid environments

Here we derive a stochastic model with one free parameter for single-cell doubling times at near-freezing temperatures. The model is derived using our experimental observations. First, we motivate a description of the doubling time in terms of a ROS-independent lower limit. This lower limit is set by the protein-synthesis rate, while the doubling time increases above this minimum through an additional time that is ROS-dependent. Using our measurements, we show a power-law type scaling between the shortest doubling time and the protein-synthesis rate. Next, we describe the protein-synthesis rate as function of temperature, such that also the shortest doubling time is a function of temperature. We further use our experimental data to derive the stochastic, ROS-dependent time in addition to the shortest doubling time. Combining the ROS-independent shortest time and the ROS-dependent additional time then yields the complete stochastic model. Finally, we fit all parts of the model to our experimental data to find the value of all model parameters.

Doubling time is dictated by the protein-synthesis rate and intracellular ROS abundance. Experimentally we have found that removing ROS with GSH shortens the doubling time at a given temperature (Supplementary Fig. 12), and that the durations of the S-G2-M phases of the cell cycle are independent of ROS (durations of S-G2-M do not change upon addition of GSH, Supplementary Fig. 25). These observations suggest that removing ROS by adding GSH decreases the doubling time to a minimum value for a given temperature (compare Fig. 2e with Fig. 4c-d). We therefore assume that there is a ROS-independent, shortest doubling time t for each temperature, and that cells having minimal ROS replicate almost as fast as that shortest doubling time. Additionally, we have found that removing ROS by adding GSH shortens G1 duration (Fig. 4c). Furthermore, cells with high intracellular ROS concentrations are unlikely to replicate (Fig. 3b) and are often arrested in G1 (Supplementary Fig. 24). These results suggest that G1 duration is ROS-dependent, which yields an additional time Δt that increases the doubling time above the ROS-independent minimum. Together, these experimental observations suggest that the single-cell doubling time τ is the sum of a ROS-independent shortest time t and a ROS-dependent additional time Δt at each temperature T, such that,

$$\tau(T, R) = t(T) + \Delta t(R, T). \tag{S11}$$

where R is some environmental parameter that depends on, for example, ROS and GSH. Here, we assume that $\Delta t > 0$ such that the doubling time is at least the shortest time for a given temperature ($\tau(T, R) > t(T)$).

Modeling the temperature dependence of the protein-synthesis rate. Recall that the protein-synthesis rate decreases as function of temperature (Supplementary Fig. 41). To describe protein

synthesis as function of temperature with a model, we assume that there exists a lower temperature limit T_m below which the yeast protein machinery fails. Then we can describe the rate as function of temperature with a common Arrhenius-type model $r_g(T) = c \cdot \exp(-a/(T - T_m))$ for some parameters a and c, such that $r_g(T) \to 0$ as the temperature decreases to T_m. Note that the Arrhenius equation would have $T_m = -273$ °C. We can rewrite this model using the experimentally measured protein-synthesis rate at a known temperature T_0,

$$r_g(T) = r_g(T_0) \cdot \exp\Big[- a\Big(\frac{1}{T - T_m} - \frac{1}{T_0 - T_m}\Big)\Big], \tag{S12}$$

providing a simple equation that describes the protein-synthesis rate as function of temperature. Taking the logarithm of equation S12 we find that,

$$\ln r_g(T) \propto -a \cdot \frac{1}{T - T_m}, \tag{S13}$$

which is a model that is linear in $1/(T - T_m)$ and that we fit to the experimentally measured protein-synthesis rates at various temperatures to find the parameter a (47).

Model for the protein-synthesis rate setting the shortest possible doubling time. We have experimentally measured the shortest doubling time $t(T)$ by taking away ROS with GSH (Supplementary Fig. 12). We have also experimentally measured the ROS-independent protein-synthesis rate $r_g(T)$ (Fig. 5d, Supplementary Fig. 43). Then both the shortest doubling time and protein-synthesis rate are independent of ROS, and we assume without loss of generality that the shortest doubling time $t(T)$ is limited by the protein-synthesis rate $r_g(T)$ at each temperature T (i.e., a cell requires a certain time to build a daughter at each temperature, regardless of ROS abundance). Here, we seek to describe the relation between the shortest doubling time and the protein-synthesis rate.

Experimentally, we have found that the observed doubling time slows down with the protein-synthesis rate via a power-law type scaling (Fig. 6b). One reason why the relation may be of power-law type is the following. We assume that the rate of each process depends on temperature according to an Arrhenius-type equation $r_p(T) = A_p \exp\Big(- \frac{E_p}{RT}\Big)$, where $r_p(T)$ is the rate of each process (i.e., protein synthesis or cell division) and constants E_p, R and A_p. Isolating the reciprocal temperature rewrites the Arrhenius equation to $\frac{1}{T} = -\frac{R}{E_p}\Big(\ln(r_p) - \ln(A_p)\Big)$. Denoting the rate of cell division as $r_d(T)$ and the rate of protein synthesis with $r_g(T)$, we can express the rate of cell division in terms of the rate of protein synthesis (omitting the temperature dependence of each rate for readability),

$$\begin{aligned} \ln(r_d) &= -\frac{E_d}{R}\Big(\frac{1}{T}\Big) + \ln(A_d) \\ &= -\frac{E_d}{R}\Big(- \frac{R}{E_g}(\ln(r_g) - \ln(A_g))\Big) + \ln(A_d) \\ &= \frac{E_d}{E_g}\ln(r_g) + c_1, \end{aligned} \tag{S14}$$

where c_1 is a constant of aggregated rest terms. Let $k = E_d/E_g$ be some factor such that by taking the exponent of equation S14 we obtain, $r_d = c_2 \cdot r_g^k$. Then, by substituting the shortest doubling time $t(T)$ for the growth rate $r_d(T)$ via $t(T) = \ln(2)/r_d(T)$ we obtain,

$$t(T) = c \cdot r_g(T)^{-k}, \tag{S15}$$

for some constant c and exponent k. Note that the above derivation is not restricted to any choice of a temperature scale. Taking the logarithm of equation S15 we find that,

$$\ln t(T) \propto -k \cdot \ln r_g(T), \tag{S16}$$

giving a linear model that we fit to the experimentally measured shortest doubling times and protein-synthesis rates at various temperatures to find the value of the exponent k. The power law with exponent k in equation S15 illustrates how the doubling time changes with protein synthesis. For example, if the protein-synthesis rate changes by two-fold, then the doubling time changes by 2^{-k}-fold, such that fold-change in the doubling time is smaller than the fold-change in protein synthesis for exponents $k < 1$. Of note, with such power law, it is only the fold-change of the protein-synthesis rate that sets how the doubling time changes, irrespective of the actual values (i.e., the doubling time and protein-synthesis rate change proportionally).

In summary, with an Arrhenius-type equation describing rates as function of temperature, we can derive that cell division and protein synthesis may follow a power-law relation. We have therefore now experimentally measured and theoretically motivated a power-law type scaling of the shortest doubling time and the protein-synthesis rate. Finally, using equation S15, we rewrite the shortest doubling time $t(T)$ using some observed shortest doubling time $t(T_0)$ at a known temperature T_0,

$$t(T) = t(T_0) \cdot \left(\frac{r_g(T)}{r_g(T_0)}\right)^{-k}. \tag{S17}$$

We know from our single-cell data that the experimentally measured shortest doubling time is stochastic and approximately normally distributed (e.g., the single-cell doubling times of the wild type at 5.0 °C with added GSH; Supplementary Fig. 12). We therefore assume that $t(T_0)$ is stochastic and equal to the experimentally measured shortest doubling time with normally distributed noise $\epsilon_m \sim N(0, 1)$, such that,

$$t(T) \sim \Big(t(T_0) + \epsilon_m\Big) \cdot \left(\frac{r_g(T)}{r_g(T_0)}\right)^{-k}, \qquad \epsilon_m \sim N(0, 1). \tag{S18}$$

We use $t(T)$ with $\epsilon_m = 0$ as the deterministic shortest doubling time $t_{\min}(T)$ from the model. The shortest doubling time at a given temeperature thus changes proportionally to the protein-synthesis rate as temperature changes. Cell-to-cell variability due to other factors is simulated with the normally distributed noise.

Stochastic model for the ROS-dependent duration of the doubling time. Having derived the ROS-independent shortest doubling time (equation S18), we here seek to describe the ROS-dependent additional time $\Delta t(R, T)$ that increases the doubling time above this shortest time. To describe this ROS-dependence, we assume that $\Delta t(R, T)$ is described by the time it takes a cell to remove sufficient intracellular ROS and repair enough damages to divide. For example, we have experimentally measured that cells with low intracellular ROS concentrations are likely to replicate whereas cells with high intracellular ROS concentrations are unlikely to replicate (Fig. 3b). We therefore assume that a cell has some amount of doubling time delaying tasks to perform before it can replicate and that this amount of time is proportional to the abundance of intracellular ROS. We further assume that these tasks can be performed in a time that is proportional to how fast a cell can express genes, for example to synthesize ROS-reducing enzymes and to replace damaged components. Thus, we assume that $\Delta t(R, T) \propto [\mathrm{ROS}]/r_g(T)$ (a cell synthesizes proteins at a rate $r_g(T)$ (arb. units per time) and has an ROS abundance of [ROS] (arb. units)). Moreover, we have experimentally measured the intracellular ROS concentrations in cells, which is approximately normally distributed on a log-scale (Supplementary Fig. 9). Finally, our measurements suggest that there is some threshold concentration of ROS above which replications become very unlikely (Fig. 3b). We therefore assume that we can describe the distribution of intracellular ROS concentrations according to $\ln[\mathrm{ROS}] \sim N(R, 1)$, where R is some parameter describing the average ROS abundance in cells (see Fig. 2a,c). Thus, $\Delta t(R, T)$ is stochastic and described by,

$$\Delta t(R, T) \sim \frac{1}{r_g(T)} \cdot \exp\Big(\epsilon_d(R)\Big), \qquad \epsilon_d(R) \sim N(R, 1). \tag{S19}$$

The additional time that increases the doubling time above its minimum thus varies between cells because of some ROS-dependent parameter that is log-normally distributed.

Stochastic model for ROS and temperature setting the doubling time. Using the ROS-independent shortest doubling time (equation S18) and the ROS-dependent addition to the doubling time (equation S19), we obtain the single-cell doubling time τ as a function of the temperature T and average ROS abundance R,

$$\begin{aligned}\tau(T, R) &= t_{\min}(T) + \Delta t(R, T)\\ &= \Big(t(T_0) + \epsilon_m\Big) \cdot \left(\frac{r_g(T)}{r_g(T_0)}\right)^{-k} + \frac{1}{r_g(T)} \cdot \exp\Big(\epsilon_d(R)\Big).\end{aligned} \tag{S20}$$

Here, $t(T_0)$ and $r_g(T_0)$ are the experimentally observed shortest doubling time and protein-synthesis rate at a temperature T_0 respectively, k is the exponent that describes the scaling between the shortest

doubling time and the protein-synthesis rate, and (from equation S12),

$$
\begin{aligned}
r_g(T) &= r_g(T_0) \cdot \exp\Big[-a \cdot \Big(\frac{1}{T - T_m} - \frac{1}{T_0 - T_m}\Big)\Big], \\
\epsilon_m &\sim N(0, 1), \\
\epsilon_d(R) &\sim N(R, 1).
\end{aligned}
\tag{S21}
$$

The parameter a describes the protein-synthesis rate as function of temperature, and T_m represents the temperature below which protein synthesis stops for yeast. With log-normally distributed ROS, we find that the single-cell doubling times are a trade-off between the speed at which a cell replicates and the likelihood of such a cell occurring. In practice, there exists a longest possible doubling time τ_{max} due to an experimentally observed threshold ROS concentration beyond which cells are unable to duplicate and die. The parameter R is the only free variable in the model, and all other parameters will be constrained next.

Model parameters. We choose the known temperature T_0 to be 5.0 °C, where we have experimentally measured $r_g(T_0) \approx 11.23$ arb. units per day (Supplementary Fig. 41) and the experimentally measured shortest doubling time $\tau(T_0) \approx 2.5$ days (with added GSH: ∼25 hours DNA replication, ∼22 hours DNA segregation, ∼4 hours in G2, and a median G1 duration of ∼9 hours; Supplementary Fig. 25). Here, we used the median G1 duration due to the shape of the distribution to more accurately estimate the shortest time spent in G1 with added GSH. We fitted the power-law type scaling (equation S15) of the shortest doubling time as function of the protein-synthesis rate to obtain an exponent $k \approx 0.77$ (Fig. 6b, Pearson correlation-coefficient $\beta = 0.998$). Only single-cell data was used as the experimental minimum doubling times (the average single-cell doubling time at 30 °C, the average single-cell doubling time with added GSH at 5.0 °C, and the minimum single-cell doubling times at 1.0 °C that exclude the duration of G1). As a lower temperature limit T_m for protein synthesis in yeast we took $T_m = -10$ °C, motivated by the fact that the yeast growth medium freezes at -2 °C (and thus limits protein synthesis by for example restricting access to nutrients, leading to deprivation of oxygen and limiting movement of (say) ribosomes), because important enzymes such as Catalase stop functioning at -6 °C [31], and other essential enzymes inactivate at 0 °C [32, 33]. It is therefore safe to assume that the temperature below which yeast's protein synthesis stops lies above -10 °C. Having fixed T_m, we fitted the temperature dependence of the protein-synthesis rate using equation S13 from which we find that $a \approx 120.72$ (Fig. 41, Pearson correlation-coefficient $\beta = 0.995$). Substituting the values of all known parameters into

equations S20-S21 yields the model that was used for all simulations,

$$\begin{aligned}\tau(T,R) &\sim (2.5+\epsilon_m)\cdot\exp\left(\frac{93}{T+10}-6.2\right)+0.09\cdot\exp\left(\frac{121}{T+10}-8+\epsilon_d(R)\right), \\ \epsilon_m &\sim N(0,1), \\ \epsilon(R) &\sim N(R,1).\end{aligned} \tag{S22}$$

This model describes the doubling time for single cells as a function of temperature T, with R representing the average abundance of ROS in cells, the only free parameter in the model. Knowing the measured (average) doubling time at a given temperature thereby constrains R through the model's predicted average doubling time. We thus constrained R as follows. We measured the average single-cell doubling time with and without added GSH at 5.0 °C (Supplementary Fig. 12). We then chose R such that the predicted doubling time of each population was equal to its measured, experimental doubling time. At 5.0 °C, this yielded R = 3.2 for populations without added GSH, and R = 0.35 for populations with added GSH (yielding Fig. 6c). We simulated R for different temperatures by taking R = 3.2 (without added GSH) and by decreasing R with 1.5% for every 0.1 °C increase in temperature (yielding Fig. 6d). Finally, we note that cells are extremely unlikely to replicate for sufficiently abundant ROS (Fig. 3b). We therefore assume that simulated cells beyond the 99-th percentile of ROS concentrations ($[ROS]_{\text{threshold}}$) are unable to replicate, yielding the slowest possible doubling times of the model.

Supplementary Discussion

Distinctions between yeast's behavior at frigid temperatures and at high temperatures. In an earlier work (Laman Trip and Youk, *Nature Microbiology* (2020)), we reported that yeast secretes glutathione at high temperatures (temperatures above $\sim$36 ^{o}C). Although GSH secretion - and cooperative growth - is only a minor focus of our current work, our discovery of yeast secreting glutathione at temperatures below $\sim$8 ^{o}C raises the question of how yeast grows differently at frigid temperatures compared to at high temperatures. Below we list the main differences between the two:

1. GSH is sufficient but not necessary to induce population growth at frigid temperatures whereas GSH alone is both sufficient and necessary to induce population growth at high temperatures. We established this difference by, for example, using a masking agent to block extracellular GSH (Supplementary Fig. 11). This observation is further supported by the fact that removing non-sugar nutrients from the growth medium also induces population growth at frigid temperatures (Fig. 2g), but not at high temperatures. Hence, extracellular GSH is not necessary - it is only sufficient - for the cooperative growth of yeast at 5 ^{o}C.

2. At high temperatures, the doubling time of a low-density population that grows with supplemented GSH is very similar to that of a high-density population that grows without a supplemented GSH. In contrast, at frigid temperatures, adding GSH not only enables low-density populations to grow, increasing its concentration can shorten the population's doubling time by several folds (Fig. 2e).

3. At high temperatures, GSH's growth-promoting actions are solely extracellular since GSH export is essential for populations to grow while GSH import is not necessary for population growths. In contrast, at frigid temperatures, GSH seems to have at least some special intracellular roles for growth given that mutants that lack certain ROS-reducing enzymes - enzymes that use GSH as a co-factor - have severely limited growth (Fig. 4e).

4. As GSH is not necessary at frigid temperatures for population growth, there are other mechanisms that, by affecting cell's ROS level, induce population growth at frigid temperatures. For example, we found that removing non-sugar nutrients also induces population growth, and we found that adding scavengers of extracellular ROS can also accelerate population growth (Supplementary Fig. 13). Import of GSH is thus not relevant as we have other means of enabling cell duplications at frigid temperatures (i.e., glutathione import is irrelevant).

5. At high temperatures, high-density populations accumulate extracellular GSH up to the amounts that we must add to the medium of a low-density population to induce growth of that low-density population. In contrast, at frigid temperatures, high-density populations cannot accumulate extracellular GSH to the amounts that we must add to a low-density population to induce growth of that low-density population. This is because, besides GSH, other factors such as non-sugar nutrients (Figs. 2f-2i) and intracellular processes play a role in determining whether a population grows at frigid temperatures

6. Knocking out some of the major heat-shock proteins - known to be important for combatting heat-induced damages at high temperatures - does not severely affect population growth in frigid temperatures (Supplementary Fig. 29).

7. At 1 ^{o}C, adding a saturating level (250 μM) of GSH cannot induce population growths and does not decrease intracellular ROS levels whereas doing so can achieve both at 5 ^{o}C. At high temperatures, however, we found that adding sufficiently high amounts of GSH is sufficient to cause population growths and extends the range of habitable temperatures to higher values.

ROS and gene-expression speed as the two primary determinants of cell's ability to duplicate and cell's doubling time at frigid temperatures. Note that "ROS is the primary determinant of X" does not mean that "ROS is the only determinant of X". We have shown that intracellular factors - such as the levels of ROS-reducing enzymes that we knocked out (Fig. 4e) - and extracellular factors - such as the amounts of ROS-generating nutrients (Figs. 2f-2i) - affect the intracellular ROS concentration. In fact, many processes that we have not considered in this work may affect the intracellular ROS concentration. Regardless of which processes and how many processes affect the ROS level in a cell, we found that the ROS concentration - determined by multiple processes - is the only information that we need to predict the cell's ability to duplicate, ability to increase in size, and probability of dying (Fig. 3). This is what we mean by "ROS is the primary determinant of X". Furthermore, this work has established that a yeast cell's doubling time and the speed limits for replicative life of yeast at each frigid temperature is determined by two factors: intracellular ROS concentration and the cell's gene-expression speed (protein-synthesis rate, in particular).

References

[1] C. Riccardi and I. Nicoletti, “Analysis of apoptosis by propidium iodide staining and flow cytometry,” *Nat. Protoc.*, vol. 1, pp. 1458–1461, 2006.

[2] O. Kandror, N. Bretschneider, E. Kreydin, D. Cavalieri, and A. L. Goldberg, “Yeast adapt to near-freezing temperatures by STRE/Msn2,4-dependent induction of trehalose synthesis and certain molecular chaperones,” *Mol. Cell*, vol. 13, pp. 771–781, mar 2004.

[3] C. Kumar, A. Igbaria, B. D’Autreaux, A.-G. Plansons, C. Junot, E. Godat, A. K. Bachhawat, A. Delaunay-Moisan, and M. B. Toledano, “Glutathione revisited: a vital function in iron metabolism and ancillary role in thiol-redox control,” *EMBO J.*, vol. 30, pp. 2044–2056, may 2011.

[4] C. M. Grant, F. H. MacIver, and I. W. Dawes, “Glutathione is an essential metabolite required for resistance to oxidative stress in the yeast Saccharomyces cerevisiae,” *Curr. Genet.*, vol. 29, pp. 511–515, 1996.

[5] D. S. Laman Trip and H. Youk, “Yeasts collectively extend the limits of habitable temperatures by secreting glutathione,” *Nat. Microbiol.*, vol. 5, pp. 943–954, apr 2020.

[6] D. J. Jamieson, “Saccharomyces cerevisiae has distinct adaptive responses to both hydrogen peroxide and menadione,” *J. Bacteriol. Res.*, vol. 174, no. 20, pp. 6678–6681, 1992.

[7] D. Giustarini, D. Tsikas, G. Colombo, A. Milzani, I. Dalle-Donne, P. Fanti, and R. Rossi, “Pitfalls in the analysis of the physiological antioxidant glutathione (GSH) and its disulphide (GSSG) in biological samples: an elephant in the room,” *J. Chromatogr. B Analyt. Technol. Biomed. Life Sci.*, vol. 1019, pp. 21–28, apr 2016.

[8] A. R. T. S. Araujo, L. M. F. S. Saraiva, and J. L. F. C. Lima, “Determination of total and oxidized glutathione in human whole blood with a sequential injection analysis system,” *Talanta*, vol. 74, pp. 1511–1519, feb 2008.

[9] G. Charvin, C. Oikonomou, E. D. Siggia, and F. R. Cross, “Origin of irreversibility of cell cycle start in budding yeast,” *PLoS Biol.*, vol. 8, no. 1, p. e1000284, 2010.

[10] P. Jorgensen, J. L. Nishikawa, B.-J. Breitkreutz, and M. Tyers, “Systematic identification of pathways that couple cell growth and division in yeast,” *Science*, vol. 297, pp. 395–400, jul 2002.

[11] M. Costanzo, J. L. Nishikawa, X. Tang, J. S. Millman, O. Schub, K. Breitkreuz, D. Dewar, I. Rupes, B. Andrews, and M. Tyers, “CDK activity antagonizes Whi5, an inhibitor of G1/S transcription in yeast,” *Cell*, vol. 117, pp. 899–913, jun 2004.

[12] P. Palumbo, M. Vanoni, V. Cusimano, S. Busti, F. Marano, C. Manes, and L. Alberghina, "Whi5 phosphorylation embedded in the G1/S network dynamically controls critical cell size and cell fate," *Nat. Commun.*, vol. 7, pp. 1–14, apr 2016.

[13] C. Garmendia-Torres, O. Tassy, A. Matifas, N. Molina, and G. Charvin, "Multiple inputs ensure yeast cell size homeostasis during cell cycle progression," *eLife*, vol. 7, p. e34025, 2018.

[14] K. M. Schmoller, J. J. Turner, M. Kõivomägi, and J. M. Skotheim, "Dilution of the cell cycle inhibitor Whi5 controls budding-yeast cell size," *Nature*, vol. 526, pp. 268–272, oct 2015.

[15] S. Morlot, J. Song, I. Léger-Silvestre, A. Matifas, O. Gadal, and G. Charvin, "Excessive rDNA transcription drives the disruption in nuclear homeostasis during entry into senescence in budding yeast," *Cell Rep.*, vol. 28, pp. 408–422.e4, jul 2019.

[16] G. Perrino, S. Napolitano, F. Galdi, A. La Regina, D. Fiore, T. Giuliano, M. di Bernardo, and D. di Bernardo, "Automatic synchronisation of the cell cycle in budding yeast through closed-loop feedback control," *Nat. Commun.*, vol. 12, pp. 1–12, apr 2021.

[17] A. P. Gasch, P. T. Spellman, C. M. Kao, O. Carmel-Harel, M. B. Eisen, G. Storz, D. Botstein, and P. O. Brown, "Genomic expression programs in the response of yeast cells to environmental changes," *Mol. Biol. Cell*, vol. 11, no. 12, pp. 4241–4257, 2000.

[18] J. Aguilera, F. Randez-Gil, and J. A. Prieto, "Cold response in Saccharomyces cerevisiae: New functions for old mechanisms," *FEMS Microbiol. Rev.*, vol. 31, pp. 327–341, apr 2007.

[19] Y. Murata, T. Homma, E. Kitagawa, Y. Momose, M. S. Sato, M. Odani, H. Shimizu, M. Hasegawa-Mizusawa, R. Matsumoto, S. Mizukami, K. Fujita, M. Parveen, Y. Komatsu, and H. Iwahashi, "Genome-wide expression analysis of yeast response during exposure to 4℃," *Extremophiles*, vol. 10, pp. 117–128, oct 2005.

[20] B. Schade, G. Jansen, M. Whiteway, K. D. Entian, and D. Y. Thomas, "Cold adaptation in budding yeast," *Mol. Biol. Cell*, vol. 15, pp. 5492–5502, dec 2004.

[21] F. Estruch and M. Carlson, "Two homologous zinc finger genes identified by multicopy suppression in a SNF1 protein kinase mutant of Saccharomyces cerevisiae," *Mol. Cell. Biol.*, vol. 13, pp. 3872–3881, jul 1993.

[22] M. T. Martínez-Pastor, G. Marchler, C. Schüller, A. Marchler-Bauer, H. Ruis, and F. Estruch, "The Saccharomyces cerevisiae zinc finger proteins Msn2p and Msn4p are required for transcriptional induction through the stress response element (STRE).," *EMBO J.*, vol. 15, pp. 2227–2235, may 1996.

[23] F. Estruch, “Stress-controlled transcription factors, stress-induced genes and stress tolerance in budding yeast,” *FEMS Microbiol. Rev.*, vol. 24, pp. 469–486, oct 2000.

[24] M. Amorós and F. Estruch, “Hsf1p and Msn2/4p cooperate in the expression of Saccharomyces cerevisiae genes HSP26 and HSP104 in a gene- and stress type-dependent manner,” *Mol. Microbiol.*, vol. 39, pp. 1523–1532, mar 2001.

[25] M. R. Grably, A. Stanhill, O. Tell, and D. Engelberg, “HSF and Msn2/4p can exclusively or cooperatively activate the yeast HSP104 gene,” *Mol. Microbiol.*, vol. 44, pp. 21–35, apr 2002.

[26] J. Zhang, C. Schneider, L. Ottmers, R. Rodriguez, A. Day, J. Markwardt, and B. L. Schneider, “Genomic scale mutant hunt identifies cell size homeostasis genes in S. cerevisiae,” *Curr. Biol.*, vol. 12, pp. 1992–2001, dec 2002.

[27] J. D. Barrass, J. E. A. Reid, Y. Huang, R. D. Hector, G. Sanguinetti, J. D. Beggs, and S. Granneman, “Transcriptome-wide RNA processing kinetics revealed using extremely short 4tU labeling,” *Genome Res.*, vol. 16, pp. 1–17, dec 2015.

[28] T. Baptista and D. Devys, “Saccharomyces cerevisiae metabolic labeling with 4-thiouracil and the quantification of newly synthesized mRNA as a proxy for RNA polymerase II activity,” *J. Vis. Exp.*, p. e57982, oct 2018.

[29] B. Neymotin, R. Athanasiadou, and D. Gresham, “Determination of in vivo RNA kinetics using RATE-seq,” *RNA*, vol. 20, pp. 1645–1652, oct 2014.

[30] K. Kawata, H. Wakida, T. Yamada, K. Taniue, H. Han, M. Seki, Y. Suzuki, and N. Akimitsu, “Metabolic labeling of RNA using multiple ribonucleoside analogs enables the simultaneous evaluation of RNA synthesis and degradation rates,” *Genome Res.*, vol. 30, pp. 1481–1491, oct 2020.

[31] K. Shikama and T. Yamazaki, “Denaturation of catalase by freezing and thawing,” *Nature*, vol. 190, pp. 83–84, 1961.

[32] P. L. Privalov, “Cold denaturation of protein,” *Crit. Rev. Biochem. Mol. Biol.*, vol. 25, no. 4, pp. 281–306, 1990.

[33] K. Gast, G. Damaschun, H. Damaschun, R. Misselwitz, and D. Zirwer, “Cold denaturation of yeast phosphoglycerate kinase: Kinetics of changes in secondary structure and compactness on unfolding and refolding,” *Biochemistry*, vol. 32, no. 30, pp. 7747–7752, 2002.
