# Supplementary material for: Slowest possible replicative life at frigid temperatures for yeast
Source: Nat Commun. 2022 Dec 6;13:7518. doi: 10.1038/s41467-022-35151-2 (PMC9726825; doi:10.1038/s41467-022-35151-2)
Supplement: Supplementary file 3 — Description of Additional Supplementary Files [file 41467_2022_35151_MOESM3_ESM.pdf]

Description of Additional Supplementary Files

Supplementary Data 1 | Pseudocodes (related to fig. 6c-d).

Together, these codes implement stochastic simulations of single cells at frigid temperatures: how each cell randomly replicates, dies, or remains alive without replicating at each time step.

Supplementary Movie 1 | Movie of a cell dying at 5 ^{O}C (related to fig 3a).

This cell dies by bursting and is representative of the typical way that a cell dies at frigid temperatures (5 ^{O}C and below). For this movie, before beginning the time-lapse imaging, we incubated the cells of a high-density population (initially ~6,250 cells per ml) at 5 ^{O}C for 14 days. Then, every day for several weeks, we imaged multiple fields of view, including one that contains the cell shown in this movie. Shown here is a cell whose size continuously increases for three days before bursting, on day 17. Movie shows brightfield images that were drift corrected and contrast enhanced with a stack histogram.

Supplementary Movie 2 | Movie of a cell increasing in size without duplicating at 5 ^{O}C (related to Fig. 3a).

This cell continuously increases in size without duplicating throughout the duration of the movie and is representative of a typical cell that grows without duplicating at frigid temperatures (5 ^{O}C and below). For this movie, before beginning the time-lapse imaging, we incubated cells of a high-density population (initially ~6,250 cells per ml) at 5 ^{O}C for 14 days. Then, every day for several weeks, we imaged multiple fields of view, including one that contains the cell shown in this movie. Movie shows brightfield images that were drift corrected and contrast enhanced with a stack histogram.

Supplementary Movie 3 | Movie of a cell duplicating at 5 ^{O}C (related to Fig. 4a).

We incubated cells of a high-density population (initially ~6,250 cells per ml) at 5 ^{O}C for 14 days prior to starting the time-lapse movie. This movie resulted from taking a snapshot twice per day for ten days. Shown here is the composite image formed merging a brightfield image with fluorescence of Whi5-mCherry (red) and H2B-GFP (green). This cell exits the G1 phase between ‘day 15.5’ and 'day 16’ (Whi5 (red) exits the nucleus and forms a bud). This cell finishes duplicating itself and enters a G1 phase (Whi5 enters the nucleus), with the bud neck snapping between ‘day 17’ and ‘day 17.5’. This cell then forms a new bud (‘day 18’) and finishes a second duplication (‘day 20’). The cell finally exits G1 (‘day 21.5’) for the remainder of the movie. Fluorescence channels show a max-z-projection. All frames were drift corrected and channels were contrast enhanced with a stack histogram.

Supplementary Movie 4 | Movie of a cell growing without duplicating at 5 ^{O}C (related to Fig. 4b).

We incubated cells of a high-density population (initially ~6,250 cells per ml) at 5 ^{O}C for 14 days prior to starting the time-lapse movie. This movie resulted from taking a snapshot twice per day for ten days. Shown here is the composite image formed merging a brightfield image with fluorescence of Whi5-mCherry (red) and H2B-GFP (green). This cell remains in G1 and continuously increases in size for at least nine days. Fluorescence channels show a max-z-projection. All frames were drift corrected, and channels were contrast enhanced with a stack histogram.

Supplementary Movie 5 | Movie of a single cell duplicating at 1 ^{O}C (related to Fig. 5a).

This cell was at 1 ^{O}C for 12 days prior to starting the time-lapse imaging. We took a snapshot of the cell every four days for several months. Shown here is the composite image formed by

merging the brightfield image with the fluorescence of Whi5-mCherry (red) and H2B-GFP (green). This cell exits G1 (Whi5 (red) exits nucleus) and forms a bud after 'day 16', as shown for 'day 20'. The cell's chromosomes segregate (H2B (green) distributes between cells) and the cell finishes duplicating with its Whi5 entering its nucleus after 'day 25', as shown for 'day 30'. Both cells remain in G1 for the remainder of the following month. Fluorescence channels show a max-z-projection. All frames were drift corrected, and channels were contrast enhanced with a stack histogram.

Supplementary Movie 6 | Movie of mCherry expression in individual cells at 5 ^{O}C (related to Fig. 5c).

Cells were incubated at 5 ^{O}C for 15 days prior to giving them galactose to induce their mCherry expression (red). Cells were then imaged twice per day for 7 days. Both cells in this movie increase in their size without duplicating for the duration of the movie. Movie shows a max-z-projection of mCherry intensity. All channels were drift corrected, and channels were contrast enhanced with a stack histogram.
