# Supplementary material for: Slowest possible replicative life at frigid temperatures for yeast
Source: Nat Commun. 2022 Dec 6;13:7518. doi: 10.1038/s41467-022-35151-2 (PMC9726825; doi:10.1038/s41467-022-35151-2)
Supplement: Supplementary file 11 — Reporting Summary [file 41467_2022_35151_MOESM11_ESM.pdf]

nature portfolio

Corresponding author(s): Hyun Youk

Last updated by author(s): Nov 15, 2022

Reporting Summary

Nature Portfolio wishes to improve the reproducibility of the work that we publish. This form provides structure for consistency and transparency in reporting. For further information on Nature Portfolio policies, see our Editorial Policies and the Editorial Policy Checklist.

Statistics

For all statistical analyses, confirm that the following items are present in the figure legend, table legend, main text, or Methods section.

n/a	Confirmed	
☐	☒	The exact sample size (*n*) for each experimental group/condition, given as a discrete number and unit of measurement
☐	☒	A statement on whether measurements were taken from distinct samples or whether the same sample was measured repeatedly
☐	☒	The statistical test(s) used AND whether they are one- or two-sided<br>*Only common tests should be described solely by name; describe more complex techniques in the Methods section.*
☒	☐	A description of all covariates tested
☒	☐	A description of any assumptions or corrections, such as tests of normality and adjustment for multiple comparisons
☐	☒	A full description of the statistical parameters including central tendency (e.g. means) or other basic estimates (e.g. regression coefficient) AND variation (e.g. standard deviation) or associated estimates of uncertainty (e.g. confidence intervals)
☐	☒	For null hypothesis testing, the test statistic (e.g. *F*, *t*, *r*) with confidence intervals, effect sizes, degrees of freedom and *P* value noted<br>*Give P values as exact values whenever suitable.*
☒	☐	For Bayesian analysis, information on the choice of priors and Markov chain Monte Carlo settings
☒	☐	For hierarchical and complex designs, identification of the appropriate level for tests and full reporting of outcomes
☐	☒	Estimates of effect sizes (e.g. Cohen's *d*, Pearson's *r*), indicating how they were calculated

Our web collection on statistics for biologists contains articles on many of the points above.

Software and code

Policy information about availability of computer code

Data collection	BD FACSDiVa 8.0<br>BioTek Synergy HTX (2018)<br>Andor IQ3 (v3.2)
Data analysis	MATLAB (R2020)<br>R Studio 3.5.1<br>ImageJ 1.53c;<br>Salmon-1.5.1<br>tximport v3.16<br><br>MATLAB codes used for modeling are publicly available at GitHub:<br>https://github.com/youklab/LamanTrip-coldTemp-2022/tree/main/Codes

For manuscripts utilizing custom algorithms or software that are central to the research but not yet described in published literature, software must be made available to editors and reviewers. We strongly encourage code deposition in a community repository (e.g. GitHub). See the Nature Portfolio guidelines for submitting code & software for further information.

Data

Policy information about availability of data

All manuscripts must include a data availability statement. This statement should provide the following information, where applicable:

- Accession codes, unique identifiers, or web links for publicly available datasets
- A description of any restrictions on data availability
- For clinical datasets or third party data, please ensure that the statement adheres to our policy

Source data are provided with this paper. Data generated in this study are available in the Source Data file and at: https://github.com/youklab/LamanTrip-coldTemp-2022 . The RNA-Seq data generated in this study are available at NCBI GEO database under accession code GSE211918 [https://www.ncbi.nlm.nih.gov/geo/query/acc.cgi?acc=GSE211918]. The Gene Ontology data for Saccharomyces cerevisiae used in this study are available at the YeastPathways data base [https://pathway.yeastgenome.org/YEAST/NEW-IMAGE?object=Gene-Ontology-Terms].

Field-specific reporting

Please select the one below that is the best fit for your research. If you are not sure, read the appropriate sections before making your selection.

☒ Life sciences ☐ Behavioural & social sciences ☐ Ecological, evolutionary & environmental sciences

For a reference copy of the document with all sections, see nature.com/documents/nr-reporting-summary-flat.pdf

Life sciences study design

All studies must disclose on these points even when the disclosure is negative.

Sample size	No sample size calculation was performed to predetermine sample size before any experiment (because our study does not use any human/clinical test subjects). Our experiments are on cell cultures. Therefore, as is the standard for experiments with cell cultures, in every experiment, we performed measurements on n biological replicates as indicated for each figure (n is at least 3 for every experiment; specific value is given in every figure). Error bars report the mean with s.e.m. for every experiment. As is the standard for cell-culture experiments like ours, n being larger than or equal to 3 is considered sufficient.
Data exclusions	We did not exclude data.
Replication	Every experiments was performed at least three independent times (precise value is reported for every data in our paper as "n = # biologically independent replicates" (# is at least 3 for every experiment). Specifically reproducibility of the experimental findings was verified by performing experiments with at least three different colonies on different days (i.e., at least n=3 biological replicates). All attempts at replication were successful and are reported as replicates.
Randomization	This is not relevant for our study because our study does not use any statistical hypothesis testing on human/clinical subjects and did not use any subjective testing. We reported all data from biological replicates. In any of our experiments which used only cells, the concept of "randomizing" cells does not make sense.
Blinding	Blinding was not necessary for our experiments because our study does not use any statistical testing on human/clinical subjects and no conclusions involved any biases (which is what blinding is used to prevent). There are no human/clinical subjects or subjective testing of any sort. Our experiments used cultures of yeast cells and we reported all the results from biological replicates. Mathematical modeling reproduced these features, further solidifying our conclusions.

Reporting for specific materials, systems and methods

We require information from authors about some types of materials, experimental systems and methods used in many studies. Here, indicate whether each material, system or method listed is relevant to your study. If you are not sure if a list item applies to your research, read the appropriate section before selecting a response.

Materials & experimental systems

n/a	Involved in the study	
☒	☐	Antibodies
☐	☒	Eukaryotic cell lines
☒	☐	Palaeontology and archaeology
☒	☐	Animals and other organisms
☒	☐	Human research participants
☒	☐	Clinical data
☒	☐	Dual use research of concern

Methods

n/a	Involved in the study	
☒	☐	ChIP-seq
☐	☒	Flow cytometry
☒	☐	MRI-based neuroimaging

Eukaryotic cell lines

Policy information about cell lines

Cell line source(s)	EUROSCARF for the wild-type strain. We built all other strains starting from the wild-type strain as explained in "Methods".
Authentication	We checked that the strains used in this study contained all the right selection markers and the genes that we inserted at the correct locus by PCR.
Mycoplasma contamination	We used yeast cells and thus we did not test for mycoplasma contamination. We did, however, check that our cell cultures genuinely involved only yeast cells with a flow cytometer and colony assays.
Commonly misidentified lines (See ICLAC register)	No commonly misidentified cell lines were used.

Flow Cytometry

Plots

Confirm that:

☒ The axis labels state the marker and fluorochrome used (e.g. CD4-FITC).

☒ The axis scales are clearly visible. Include numbers along axes only for bottom left plot of group (a 'group' is an analysis of identical markers).

☒ All plots are contour plots with outliers or pseudocolor plots.

☒ A numerical value for number of cells or percentage (with statistics) is provided.

Methodology

Sample preparation	Yeast cells were taken from single colonies on agar pads. They were then incubated in clear, transparent minimal media (described in the methods) for the growth experiments. We then took aliquots (usually 200 uL) from the liquid culture and transferred each aliquot into a well in a 96-well plate. The High-Throughput Sampler of BD FACSCelesta injected these samples into the flow cytometer.
Instrument	BD FACSCelesta with High-Throughput Sampler and three lasers: 405 nm (violet), 488 nm (blue), 561 nm (yellow/green)
Software	MATLAB R2020
Cell population abundance	We reported all yeast cells that the flow cytometer detected; we did not exclude any yeast cells. As a control, flowing PBS without any cells yielded no detected events in the gate settings that we used
Gating strategy	Supplementary Fig. 44c shows the gating strategy.<br><br>Moreover, the Methods section describes the settings of our flow cytometer. We repeat this information below:<br><br>For GFP: PMT: 485V, Filter: 530/30, Mirror: 505LP, Channel: FITC, Blue laser (488nm)<br>For mCherry: PMT=498V, Filter 640/40, Mirror: 595LP, Channel: mCherry, Yellow-green laser - (561nm)<br>The choice for mCherry gates for PI staining did not change the results that we reported, as populations of stained cells and unstained cells were separated by several orders of magnitude.

☒ Tick this box to confirm that a figure exemplifying the gating strategy is provided in the Supplementary Information.
